# Recent Progress in Exosome-Derived Nanocarriers for Breast Cancer Therapy: Advances, Translational Barriers, and Scale-Up Considerations

**DOI:** 10.1007/s11095-026-04095-3

**Published:** 2026-04-21

**Authors:** Julie R. Youssef, Esraa M. Elgammal, Dina M. Mahdy, Noha F. Ghazi, George Bebawy

**Affiliations:** 1 Pharmaceutics Department, College of Pharmacy, Arab Academy for Science, Technology and Maritime Transport (AASTMT), Alamein, Egypt; 2 College of Pharmacy, Arab Academy for Science, Technology and Maritime Transport (AASTMT), Alamein, Egypt; 3 Pharmaceutics Department, Faculty of Pharmacy, Mansoura University, Dakahlyia, Egypt; 4 Pharmaceutics Department, Faculty of Pharmacy, Alexandria University, 1 Khartoum Square, Alexandria, Azarita Egypt

**Keywords:** breast cancer, drug delivery, exosomes, extracellular vesicles, phytochemical therapeutics, targeted therapy, translational nanomedicine

## Abstract

Breast cancer remains the most prevalent malignancy among women worldwide and a major cause of cancer-related mortality. Despite remarkable progress in molecularly targeted and immune-based therapies, therapeutic resistance, dose-limiting systemic toxicity, and inefficient drug delivery continue to hinder clinical outcomes, particularly in aggressive subtypes such as triple-negative breast cancer. Exosomes, naturally secreted nanosized vesicles, have emerged as a transformative platform owing to their biocompatibility, intrinsic targeting capability, and ability to transport diverse therapeutic cargos across biological barriers. Recent advances in exosome biology, engineering, and isolation technologies have reignited interest in their clinical exploitation as drug delivery systems. However, translation into clinical oncology remains in its early stages. This review provides a comprehensive overview of exosome-based drug delivery systems specifically within the context of breast cancer therapy, critically evaluating their sources, isolation techniques, cargo loading strategies, targeting mechanisms, and formulation considerations. It also examines preclinical findings demonstrating enhanced therapeutic efficacy and reduced off-target toxicity, alongside the limited yet growing number of clinical trials investigating exosome therapeutics in solid tumours. Importantly, the review identifies major gaps, including lack of standardized manufacturing protocols, incomplete pharmacokinetic understanding, and unresolved safety concerns, that currently impede clinical translation. By bridging molecular insights with translational perspectives, this work underscores the untapped potential of exosomes as next-generation drug carriers in breast cancer. It highlights the urgent need for harmonized methodologies, scalable production systems, and regulatory frameworks to enable their safe and effective integration into future cancer therapeutics.

**Graphical Abstract:**

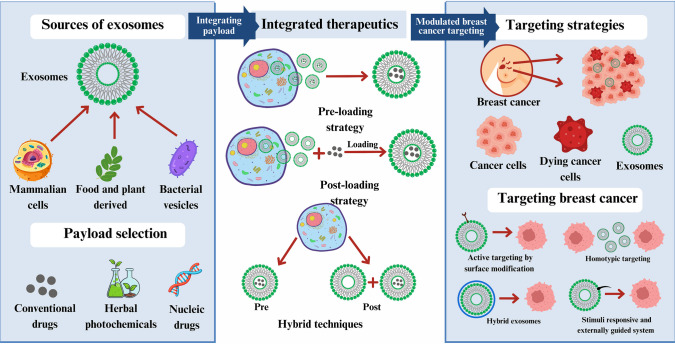

## Introduction

Breast cancer remains the most frequently diagnosed malignancy among women globally and continues to be a leading cause of cancer-related mortality. Recent global estimates indicate that approximately 2.3 million new cases and 670,000 deaths occurred in 2022, underscoring its substantial public health impact. If current incidence trends persist, global projections estimate that annual cases could reach around 3.2 million, with over one million breast cancer-related deaths per year by 2050, with approximately one in twenty women expected to develop breast cancer during their lifetime, disproportionately affecting countries with low Human Development Index (HDI) levels [1, 2].

Among its subtypes, triple-negative breast cancer (TNBC), which lacks expression of oestrogen receptor (ER), progesterone receptor (PR), and Human Epidermal Growth Factor Receptor 2 (HER2), accounts for approximately 15–20% of all breast cancers, yet contributes disproportionately to mortality due to its aggressive behaviour, early metastasis, and lack of targeted therapies [3]. Despite progress in hormonal and HER2-targeted therapies, as well as emerging immunotherapies, major unmet clinical needs persist. such as resistance to treatment, substantial systemic toxicity, and limited efficacy in aggressive subtypes such as TNBC [4].

Conventional drug-delivery approaches, including systemic chemotherapy, small molecule inhibitors, and antibody–drug conjugates (ADCs), although effective in many settings, often suffer from poor tumour selectivity, off-target adverse effects (e.g. cardiotoxicity, neurotoxicity), rapid drug clearance, low therapeutic index, and insufficient penetration into tumour microenvironments or metastatic sites such as brain or lung [5]. Moreover, in metastatic breast cancer, especially in TNBC, these limitations contribute to high relapse rates and poor long-term survival [6].

In this context, exosomes have emerged as promising natural nanocarriers for drug delivery, offering several unique advantages relative to synthetic nanoparticle systems. Exosomes are small (approximately 30–150 nm) extracellular vesicles derived from the endosomal pathway; their membranes are enriched in lipids (cholesterol, sphingomyelin, ceramide), transmembrane proteins including tetraspanins, integrins, and adhesion molecules, which confer both structural stability and tissue tropism [7]. Their capacity to encapsulate both hydrophilic and hydrophobic molecules, evade immune detection, cross biological barriers (such as the blood–brain barrier), and facilitate internalization via endocytosis or membrane fusion make them an especially attractive platform to deliver diverse payloads to breast tumours [8]. These payloads can range from conventional chemotherapeutics to nucleic acids, repurposed drugs, and herbal bioactives.

Exosome-based drug delivery systems represent a promising strategy in cancer therapy by enhancing therapeutic efficacy, enabling personalized treatment, and addressing limitations of conventional approaches [9]. Their high biocompatibility, intrinsic targeting capability, and capacity to transport diverse cargos, including chemotherapeutic agents, nucleic acids, and immunomodulators enable selective delivery to cancer cells while reducing off-target toxicity. Extensive preclinical and early clinical studies highlight their potential; however, challenges related to large-scale production, standardization, quality control, and regulatory safety remain a big hurdle [9]. Advances in exosome biogenesis, cargo loading, targeting strategies, and engineered or hybrid platforms are expected to further support clinical translation in precision oncology.

Bioengineering approaches have substantially improved exosome tumour specificity and therapeutic performance. Surface functionalization with tumour-targeting ligands, such as peptides, antibodies, or aptamers, enhances binding to overexpressed receptors including HER2, EGFR, and RGD-binding integrins through chemical conjugation or genetic engineering strategies [10]. Hybrid and externally guided systems, such as magnetic nanoparticle-coupled exosomes, further increase tumour accumulation under external guidance [11]. Engineered exosomes loaded with chemotherapeutics or nucleic acids demonstrate improved pharmacokinetics, tissue penetration, and antitumor efficacy compared with native vesicles [12]. Scalable manufacturing platforms, including three-dimensional microcarrier culture combined with tangential flow filtration, significantly enhance exosome yield and clinical feasibility [13]. Targeted formulations, such as HER2-directed exosomes delivering miR-34a [14] or siRNA via fusion proteins (e.g., Laml2b-DARPin G3) [15], have shown enhanced tumour accumulation and gene silencing in breast cancer models.

This review highlights recent advances in exosome engineering and formulation strategies for breast cancer, encompassing targeted, hybrid, and externally guided delivery systems, as well as preclinical and early clinical findings and scalable production approaches. By integrating these developments, we provide a comprehensive perspective on translating exosome therapeutics from bench to bedside, outlining both the opportunities for precision oncology and the challenges that must be addressed to achieve clinical implementation.

## Exosome Sources and Production Challenges

Exosomes are secreted by virtually all cell types and are found in a broad spectrum of biological fluids, including serum, plasma, urine, saliva, cerebrospinal fluid, breast milk, and in the conditioned media of cultured cells [16]. Successful clinical translation of exosome-based therapeutics critically depends on the ability to obtain sufficient yields with consistent quality, safety, and functional integrity from these diverse sources. Among mammalian sources, mesenchymal stem cells (MSCs) are widely used due to their high secretory capacity, ease of expansion, low immunogenicity, and retention of beneficial paracrine effects while avoiding risks associated with cell transplantation, such as immune rejection or uncontrolled growth [17–19].

In contrast, immune cell-derived exosomes (e.g., from dendritic cells or macrophages) can actively modulate immune responses and have been explored as cell-free vaccines or immune-stimulating platforms, but their production yields are generally lower and standardization remains a challenge [20–22]. Tumour-derived exosomes inherently carry tumour-associated antigens and homotypic targeting signals, which may be useful in disease modelling or biomarker discovery; however, their potential to carry pro-tumorigenic cargo and the difficulty of ensuring safety limit their direct use as therapeutic carriers without rigorous modification and safety controls [22].

Exosome secretion is sensitive to cellular stressors, oxygen tension, nutrient composition, serum supplementation, and other culture parameters. For example, hypoxia often enhances exosome release [23]. Strategies to enhance exosome production include biochemical induction (e.g., small-molecule modulators of the ceramide pathway), genetic engineering of Rab GTPase or Endosomal Sorting Complex Required for Transport (ESCRT) pathway components, and mechanical stimulation (e.g., shear stress or microfluidic agitation), forming a three-sided optimization scheme to standardize and amplify yields for therapeutic deployment [24].

In addition to mammalian sources, non-mammalian or host-agnostic vesicles, such as those isolated from bovine milk or plants, provide highly scalable, cost-effective sources with low immunogenicity. These have been explored for delivering bioactive compounds in preclinical models, although translational data in humans are still limited [21, 25–27].

Among mammalian and non-mammalian sources, mesenchymal stem cell (MSC)-derived exosomes currently represent the most practical option for clinical translation due to their high scalability, reproducible production, low immunogenicity, and established safety profile [17, 21, 25, 28]. In contrast, immune-cell or tumour-cell-derived exosomes may be more suitable for immunomodulatory or diagnostic applications, while non-mammalian sources, such as milk- or plant-derived vesicles, provide an emerging, scalable alternative with low immunogenicity [29–31].

Several preclinical studies have demonstrated the feasibility of using exosomes derived from different biological sources as therapeutic delivery vehicles for breast cancer treatment. For instance, Sheykhhasan *et al*. demonstrated that exosomes derived from adipose-derived mesenchymal stem cells (MSCs) can be utilized as carriers for the delivery of miR-145 to breast cancer cells. Exosome-mediated transfer of miR-145 resulted in the downregulation of target genes associated with tumour invasion and metastasis, including Rho-Associated Coiled-Coil Containing Protein Kinase 1 (ROCK1), Erb-B2 receptor tyrosine kinase 2 (ERBB2), and matrix metalloproteinase 9 (MMP9), indicating anti-metastatic effects and highlighting the potential of MSC-derived exosomes as promising vehicles for nucleic acid-based therapeutic delivery in breast cancer [32]. Similarly, Hosseini *et al*. demonstrated that exosomes derived from Wharton’s jelly mesenchymal stem cells (WJ-MSCs) can act as nanocarriers for the signal transducer and activator of transcription 3 (STAT3) inhibitor S3I-201, enhancing its antitumor efficacy and reducing tumour growth in breast cancer-bearing mouse models [33]. Alternative scalable vesicle sources have also been explored in breast cancer models. For example, Ramezani *et al*. showed that bovine milk-derived exosomes loaded with lactoferrin (lactoferrin-loaded exosomes; exoLF) exert significant antiproliferative effects against the MDA-MB-231 breast cancer cell line, highlighting the potential of food-derived vesicles as cost-effective and biocompatible drug delivery systems [34].

Further advances in engineering strategies have demonstrated the potential of exosome-based systems for targeted chemotherapy delivery in breast cancer models. For example, Tian *et al*. developed engineered dendritic-cell-derived exosomes loaded with doxorubicin (DOX) and modified with the tumour-homing internalizing RGD (iRGD) peptide, enabling selective delivery to αv-integrin-positive breast cancer cells and significant tumour growth inhibition *in vivo* while reducing systemic toxicity [35]. In addition, biomimetic hybrid systems have been explored to further enhance targeting and therapeutic efficiency. Yong *et al*. developed exosome-sheathed porous silicon nanoparticles loaded with DOX, which exhibited enhanced tumour accumulation, deeper penetration into tumour tissue, and increased cytotoxicity against both bulk tumour cells and cancer stem cells in preclinical tumour models [36].

Collectively, these studies highlight the potential of exosomes from diverse biological sources as therapeutic delivery platforms for breast cancer, while underscoring the need for scalable and standardized production for clinical translation.

## Exosome Isolation Techniques

A central hurdle in exosome research is achieving high-purity isolation [37]. Commonly employed isolation strategies offer distinct advantages and drawbacks. The ideal method would combine high yield, purity, structural integrity, scalability, and reproducibility.

### Differential Method (Ultracentrifugation and Density Gradient)

Differential ultracentrifugation (UC) remains a benchmark in the field. While UC offers relatively high yield and broad accessibility, drawbacks include co-pelleting of protein aggregates or lipoproteins, potential vesicle damage due to high shear [38]. Density gradient centrifugation can refine purity by separating vesicles by buoyant density; indeed, density gradient methods often yield the cleanest exosome populations compared to basic UC or precipitation methods [39]. Nevertheless, for large-volume or clinical-scale applications, pure density gradient centrifugation is time-intensive and resource-demanding, motivating the use of hybrid downstream methods.

Differential ultracentrifugation remains one of the most widely used methods for isolating exosomes in cancer research. For example, Hoshino *et al*. isolated tumour-derived exosomes from breast cancer cell lines and patient plasma using sequential ultracentrifugation combined with density-gradient purification to investigate exosomal integrin profiles associated with organ-specific metastasis. The findings showed that distinct exosomal integrins contribute to pre-metastatic niche formation, demonstrating the suitability of ultracentrifugation-based isolation for functional studies of tumour-derived exosomes [40].

### Membrane Filtration and Ultrafiltration Method

Membrane-based filtration uses size-exclusion through membranes with defined molecular weight cutoffs (MWCO) to separate exosomes from larger debris [41]. Tangential flow filtration (TFF) is a scaled-up version, allowing continuous flow and reducing clogging [42]. Advantages include faster processing and moderate scalability; disadvantages include potential shear stress, adsorption losses, and partial retention of soluble proteins. Filtration may also act as a pre-concentration step preceding more selective purification [42].

Recent studies have highlighted the advantages of TFF as a scalable strategy for isolating extracellular vesicles. For example, Visan *et al*. performed a comparative analysis of TFF and ultracentrifugation, both followed by size-exclusion chromatography, for the isolation of small extracellular vesicles from several cancer cell lines, including the human breast cancer cell line MDA-MB-231 [43]. The results showed that TFF produced significantly higher vesicle yields while maintaining comparable size distribution and characteristic exosomal markers such as CD9, TSG101, and HSP70. These findings highlight the potential of TFF-based workflows to improve reproducibility, processing efficiency, and scalability compared with traditional ultracentrifugation approaches.

### Size-Exclusion Chromatography (SEC)

Size-Exclusion Chromatography separates vesicles by size using porous matrices (e.g., Sepharose). It is gentle and preserves vesicle integrity, avoiding harsh forces [44]. In most cases, SEC yields exosome preparations with lower protein contamination and better functional retention than UC or precipitation methods [45, 46]. However, its resolution is limited, and throughput is limited without scale-up adaptation.

This technique has emerged as an effective approach for isolating circulating extracellular vesicles from plasma samples due to its ability to preserve vesicle integrity while reducing protein contamination. For example, Baranyai *et al*. demonstrated that SEC enables efficient purification of exosomes from human plasma with significantly lower co-isolated protein levels compared with conventional ultracentrifugation methods, highlighting its suitability for downstream extracellular vesicle characterization and biomarker studies [47]. This strategy has also been explored in cancer liquid biopsy studies, including breast cancer, to isolate circulating tumour-derived exosomes carrying diagnostic cargo such as microRNAs and oncogenic proteins associated with tumour progression and metastasis [48].

### Immunoaffinity Capture

Antibodies or aptamers against exosomal surface markers (e.g., CD9, CD63, CD81, epithelial cell adhesion molecule EpCAM) can selectively bind exosomes from complex mixtures. This yields high specificity but typically lower recovery and may bias toward subpopulations that express the targeted marker. For therapeutic exosomes, loss of functional subtypes is a concern; additionally, scale-up is challenging and costly [49].

Immunoaffinity-based isolation methods have been widely used to selectively enrich tumour-derived exosomes expressing cancer-associated surface markers. For example, Fang *et al*. developed a microfluidic immunocapture platform using antibodies against exosomal markers such as CD63 and Epithelial cell adhesion molecule (EpCAM) to selectively isolate and quantify circulating exosomes from the plasma of breast cancer patients [50]. This approach enabled efficient capture of tumour-associated exosomes and demonstrated potential utility for breast cancer diagnosis and molecular classification. Although immunoaffinity-based methods provide high specificity and purity, they often yield lower vesicle quantities and involve higher costs compared with bulk isolation techniques, which may limit their scalability for large-volume exosome production.

### Precipitation Methods

Precipitation using polymers like polyethylene glycol (PEG) or PROSPR (pre-oxidation and reversal separation) offers ease of use and high yield, especially for analytical work. However, these methods co-precipitate proteins, lipoproteins, and other contaminants that degrade downstream performance, particularly in therapeutic settings [51].

These polymer-based precipitation approaches are widely used for isolating exosomes from serum samples due to their simplicity and high recovery efficiency. For example, Jung *et al*. compared ultracentrifugation (UC) with polymer-based precipitation using ExoQuick and membrane-affinity column methods for isolating serum-derived exosomes from patients with triple-negative breast cancer (TNBC) [52]. Their results showed that precipitation methods yielded a higher number of vesicles, whereas UC produced preparations with higher purity. Importantly, exosomes isolated using these different approaches enabled cytokine profiling, revealing distinct exosomal cytokine patterns depending on the isolation method.

### Emerging Microfluidic, Acoustic, Magnetic and Nanotechnology-Assisted Methods

In recent years, advanced microfluidics and nanotechnology-based isolation platforms have emerged, offering label-free, high-resolution separation from small volumes. These include microfluidic mixing, nanoporous membranes, acoustic separation, dielectrophoresis, immunomagnetic enrichment, and field-flow fractionation [53]. For example, field-flow isolation, label-free magnetic sorting, and micro-nanostructure platforms have shown high efficiency in isolating exosomes with minimal contamination [54]. While promising, many of these remain in research-phase with challenges in throughput, scale, and regulatory compatibility.

Advanced microfluidic and nanotechnology-based platforms have been developed to isolate tumour-derived exosomes from small clinical samples in breast cancer diagnostics. For example, Wu *et al*. reported an acousto-fluidic microfluidic device capable of isolating exosomes directly from whole blood with high purity and efficiency through integrated acoustic separation modules [55]. This system enables label-free isolation of extracellular vesicles while removing blood cells and larger particles, providing a promising approach for rapid exosome isolation suitable for downstream cancer biomarker analysis and liquid biopsy applications.

#### Microfluidic Mixing

Emerging microfluidic approaches exploit engineered microchannel structures and controlled fluid dynamics to enhance passive isolation of exosomes based on size or affinity interactions while minimizing the need for bulky instrumentation. Microfluidic devices can employ either passive hydrodynamic forces or active capture mechanisms to separate extracellular vesicles with improved efficiency and reduced sample processing time compared with conventional techniques [56].

In breast cancer research, several integrated microfluidic platforms have been developed that combine microstructured capture surfaces with controlled flow dynamics to enhance interactions between extracellular vesicles and capture interfaces. For example, a nickel-doped microfluidic chip employing immunomagnetic separation achieved exosome recovery rates exceeding 70% with high purity, demonstrating its potential for rapid and efficient exosome isolation from complex samples [57]. Other microfluidic systems integrate micromixing architectures and antibody-functionalized magnetic beads to selectively enrich tumour-associated extracellular vesicles expressing epithelial markers such as EpCAM from patient plasma, thereby improving detection sensitivity in breast cancer diagnostics [58]. Additionally, integrated microfluidic devices capable of on-chip immunomagnetic separation and detection have been developed to isolate circulating exosomes directly from blood samples, enabling rapid analysis within approximately 1.5 h and achieving diagnostic sensitivity around 90% and specificity exceeding 95% for distinguishing breast cancer patients from healthy controls [59].

These microfluidic platforms have also been reviewed as compact systems capable of label-free or affinity-based exosome isolation with improved throughput, reduced reagent consumption, and potential suitability for point-of-care diagnostic applications compared with traditional isolation techniques [60, 61].

#### Nanoporous Membrane Based Isolation

Nanoporous membrane–based strategies isolate exosomes using membranes with precisely defined pore sizes (typically ~ 30–200 nm), enabling size-selective transport of extracellular vesicles while retaining larger particles and cellular debris. Diffusion-based nanoporous membrane separation relies on concentration-driven transport of nanoscale vesicles across nanopores without applying strong external forces, which helps preserve vesicle integrity during isolation. For example, diffusion-based nano-sieve platforms employing engineered nanoporous membranes have demonstrated efficient extracellular vesicle isolation from biological fluids with high recovery and reduced mechanical stress compared with conventional ultracentrifugation methods [62]. An alternative approach is bidirectional flow filtration (BFF), in which sequential nanoporous membranes are combined with alternating flow directions to reduce membrane fouling and improve separation efficiency. This strategy enables continuous processing and size-selective enrichment of extracellular vesicles while minimizing pore blockage, thereby improving purity and operational stability compared with conventional filtration approaches [63]. Overall, nanoporous membrane platforms offer several advantages, including gentle processing conditions, scalability, and compatibility with continuous-flow systems. These characteristics make them promising technologies for high-purity exosome isolation in both research applications and therapeutic manufacturing settings [64].

#### Acoustic Separation

Acoustic separation (acoustofluidics) exploits acoustic radiation forces generated by surface acoustic waves or bulk acoustic waves within microfluidic channels to separate particles based on differences in size, density, and compressibility. When acoustic fields are applied, larger cellular components experience stronger acoustic forces and are displaced differently from nanoscale extracellular vesicles, allowing selective enrichment of exosomes without physical contact or chemical labels [65]. Several acoustofluidic platforms have demonstrated efficient extracellular vesicle isolation from complex biofluids. For example, integrated acoustic microfluidic devices have been used to isolate exosomes directly from whole blood by sequentially removing larger particles such as cells and microvesicles before enriching nanoscale vesicles, enabling rapid and high-purity exosome separation suitable for downstream molecular analysis [66, 67]. Acoustic isolation strategies offer several advantages compared with conventional centrifugation-based methods, including label-free operation, minimal sample preparation, continuous processing, and high biocompatibility, making them promising tools for rapid extracellular vesicle isolation in diagnostic and therapeutic applications [68].

#### Dielectrophoresis (DEP)

Dielectrophoresis (DEP) is a label-free electrokinetic separation technique that manipulates particles using forces generated in non-uniform electric fields, enabling discrimination based on differences in dielectric properties, size, and membrane composition [69].

Recent studies have demonstrated that integrating DEP with microfluidic platforms enables selective enrichment of nanoscale vesicles by tuning the applied electric field and electrode configuration, allowing effective isolation of exosomes from mixed particle populations without the need for biochemical labelling [70].Such DEP-enabled devices can operate with small sample volumes and enable rapid, controllable separation while maintaining vesicle integrity [71].

Despite these advantages, challenges remain in optimizing electrode design, electric field parameters, and throughput to support routine clinical use and large-scale extracellular vesicle processing [69].

Collectively, these emerging technologies provide complementary strategies for exosome isolation, offering advantages such as label-free separation, reduced processing time, high recovery efficiency, and compatibility with microfluidic platforms. Continued development and optimization of these systems may further enable scalable and clinically applicable exosome isolation for diagnostic and therapeutic applications.

## Isolation Techniques Considerations

Although various techniques exist, the optimal exosome isolation workflow is highly source-dependent and must balance yield, purity, preservation of functional cargo, and scalability. A comparative overview of the most commonly used isolation methods, together with their key advantages and limitations, is provided in Table I.

**Table I:** Summary of Major Exosome Isolation Methods, With Their Principal Advantages and Limitations Relevant to Yield, Purity, Scalability, and Clinical/Translational Applicability

Isolation Method	Advantages	Disadvantages	Ref
Differential Ultracentrifugation	• Widely used “gold standard”, cost-effective, ultracentrifuges widely available in many labs • Handles large sample volumes (few mL to > 100 mL) • Allows parallel processing of multiple samples	• Time-consuming; Often requires additional steps (e.g., ultrafiltration) to improve purity • Co-purifies proteins and aggregates • High g-forces may damage EVs • Limited scalability • Requires expensive specialized equipment	[72–77]
Density Gradient Ultracentrifugation	• Yields purer EVs • Separates by density • Enriches target proteins	• Time-intensive • Requires high starting EV concentration • Contaminants of similar density may co-purify • Complex workflow • Highly sensitive to centrifugation time and protocol variations	[73, 78–81]
Immunoaffinity Isolation	• Highly specific for EV subpopulations expressing chosen surface markers • Can isolate EVs directly from small-volume samples • Compatible with downstream analyses (e.g., mass spectrometry, RNA sequencing) • Immunoaffinity separates EVs based on surface markers • Can isolate EVs directly from biological fluids without prior extensive purification	• Not suitable for large volume samples; low EV content requires prior concentration • Isolates only a subset of EVs; may miss other populations • Elution from beads may slightly alter EV size or surface structure; functional studies can be challenging • Additional steps make the process lengthy • Prone to non-specific protein binding if bead surfaces are not properly blocked	[81–88]
Microfluidics	• Highly specific for chosen EV subpopulations • Works with small-volume samples • Compatible with downstream analyses • Enables simultaneous EV isolation and detection	• Not suitable for large volumes; isolates only subpopulations • Elution may slightly alter EVs; functional studies challenging • Often requires additional steps • Low throughput due to single-channel designs • Microfluidic chips may clog, limiting reuse and lifespan	[76, 83–86, 89]
Size Exclusion Chromatography (SEC)	• Rapid (10–20 min) and cost-effective • Produces pure, intact, and biologically active EVs; reduces aggregation • Efficiently separates EVs from soluble proteins and HDL; allows buffer exchange with minimal effects on EVs	• Dilutes EV samples, often requiring re-concentration • Slightly lower yield than some precipitation methods • Residual apolipoproteins may remain associated with EV fractions	[73, 90–93]
Ultrafiltration (UF)	• Simple, cost-effective, and can handle variable sample volumes • Fast and efficient; preserves EV morphology and marker expression • High recovery (up to 80%) and concentration (up to 240-fold); suitable for large volumes	• Potential non-specific binding to membranes, causing some EV loss • EV yield may be lower for small initial volumes; initial fractions may lose some EVs	[77, 83, 94]

## Exosome Characterization Techniques

Characterizing exosomes is essential for understanding their size, concentration, composition, and functionality, which is critical for both basic research and translational applications. Various analytical techniques are available, ranging from conventional immunoassays to advanced single-particle detection platforms. Table II summarizes the main exosome characterization techniques, highlighting their key advantages and limitations.

**Table II:** Summary of Common Exosome Characterization Techniques, Including Main Advantages and Disadvantages of Each Technique

Technique	Advantages	Disadvantages	Ref
ELISA	• Sensitive and highly specific for exosomal markers • Simple, quick, and widely accessible • Low-cost reagents	• Requires monoclonal antibodies • Potential for nonspecific binding or cross-reactivity • Possible false positives/negatives • Time-consuming	[120]
Flow Cytometry	• Uses standard laboratory instruments • Allows fluorescent labelling of EVs for analysis	• Limited detection for smaller vesicles (< 150 nm)	[121]
Nanoparticle Tracking Analysis (NTA)	• Provides absolute particle concentration, measures exact size • High-resolution analysis Fluorescent NTA instruments are readily available	• Aggregated vesicles are difficult to resolve	[95]
Tuneable Resistive Pulse Sensing (TRPS/qNano)	• Measures absolute EV concentration and size distribution • Can detect particle surface charge • Suitable for exosome-sized particles	• Requires different pore sizes for various sample types	[120]
Electrochemical Detection	• Highly sensitive and specific • Low-cost detection • Requires only small sample volumes	• Complex fabrication; can be difficult to handle	[117]
Dynamic Light Scattering (DLS)	• Fast and convenient • Commonly used for colloidal systems	• Cannot resolve mixtures with widely different particle diameters	[122]
Raman Spectroscopy (SERS)	• Can distinguish EVs from different sources • Allows detection of individual vesicles • Applicable for clinical diagnostics	• Inefficient for conventional Raman • Requires high sample concentration, strong laser power, and long acquisition time	[123]
Plasmonic Detection (nPLEX)	• Rapid and highly sensitive label-free detection of small EVs	• Requires specialized and costly instrumentation	[59]

### ELISA

ELISA is widely used to detect and quantify exosomes by capturing them on antibody-coated plates targeting surface markers such as CD9 or CD63, followed by enzymatic signal amplification [95–97]. Sandwich ELISA allows detection in culture media or plasma, including tumour-associated markers like CD63, Rab-5b, and caveolin-1 [98]. Advanced platforms like ExoView use single-particle interferometric reflectance imaging sensing (SP-IRIS) to analyse individual EVs as small as 50 nm, providing precise sizing and quantification with higher sensitivity than Western blotting [99]. In breast cancer research, exosome characterization is commonly performed on vesicles isolated from conditioned media of breast cancer cell lines or from patient plasma following ultracentrifugation-based isolation. For instance, Hannafon *et al*. isolated exosomes from MCF-7 and MDA-MB-231 culture media using differential ultracentrifugation and characterized them using nanoparticle tracking analysis (NTA), electron microscopy, immunogold labeling, and Western blot detection of the exosomal marker CD63. These analyses supported the identification of exosome-associated microRNA profiles with potential relevance for breast cancer diagnostics [100].

### Flow Cytometry

Flow cytometry enables high-throughput quantification and multiparameter analysis of EVs by measuring light scattering and fluorescence [101]. EV size is inferred by correlating scattered light intensity with particle diameter, often calibrated with standard beads [72, 98]. Magnetic beads coated with antibodies (e.g., MHCII molecules) capture exosomes, which are then fluorescently labelled for sorting [72, 102, 103]. Advanced instruments like Amnis Cell Stream with TDI imaging or NanoFCM allow analysis of single EVs down to ~ 40 nm, facilitating precise profiling and early disease biomarker studies [104, 105]. Flow cytometry in breast cancer exosome studies is often combined with selective capture approaches to enrich tumour-derived extracellular vesicles. For example, studies combining immunomagnetic separation with flow cytometric analysis have profiled circulating exosomes from breast cancer patients by capturing vesicles with antibodies against tumour-associated markers such as CD24, CD44, and epithelial cell adhesion molecule (EpCAM), followed by flow cytometric analysis of surface markers to distinguish tumour-derived from non-tumour exosomes [106, 107].

### Nanoparticle Tracking Analysis (NTA)

NTA tracks Brownian motion of nanoparticles in liquid to determine size distribution and concentration [99]. Light scattering is captured via laser and camera, and the Stokes–Einstein equation converts particle motion into size [95, 108]. Fluorescent labelling of tetraspanins (CD63, CD81) enables phenotyping [109]. NTA detects vesicles as small as ~ 50 nm and handles large populations, though analysis is time-consuming and requires well-purified samples, with potential photobleaching during long acquisitions [95]. NTA is widely used to characterize exosome size distribution and particle concentration following isolation from breast cancer sources. For example, studies have isolated exosomes from breast cell lines using differential ultracentrifugation or combined ultrafiltration–ultracentrifugation approaches and subsequently characterized vesicle size and concentration using NTA [110]. These analyses support downstream proteomic and molecular profiling of exosomal cargo, enabling the identification of tumour-associated proteins and microRNAs relevant to breast cancer progression.

### Tuneable Resistive Pulse Sensing (TRPS)

TRPS measures individual vesicles by detecting transient ionic current changes as particles pass through tuneable nanopores [95, 111, 112]. Each pulse is proportional to particle volume, allowing accurate size and concentration measurements from 40 nm to 10 μm, with concurrent zeta potential determination. TRPS can discriminate subpopulations, assess polydispersity, and study nano-bio interactions [113]. In breast cancer exosome research, TRPS is frequently applied following enrichment of extracellular vesicles through techniques such as ultracentrifugation or size-exclusion chromatography to determine vesicle concentration and size heterogeneity. TRPS provides single-particle analysis of extracellular vesicles, enabling accurate measurement of particle size distribution and concentration. Isolation approaches that preserve vesicle integrity, such as size-exclusion chromatography, generate purified exosome preparations compatible with TRPS analysis for sensitive profiling of extracellular vesicle subpopulations in cancer biomarker studies [113–115].

### Electrochemical Detection

Electrochemical methods detect EVs through antibody-mediated recognition of surface markers (CD9, CD63, CD81), generating measurable signals [95, 116]. Integrated magneto-electrochemical sensors (iMEX) allow rapid detection from 10 μL samples within one hour [112, 117]. Sandwich immunosensors and quantum dot-based assays improve sensitivity, enabling detection of 100–200 EVs/μL [95, 118]. Although fast and cost-effective, these platforms require complex fabrication and benefit from microfluidic integration [95]. Electrochemical detection platforms are frequently integrated with immunoaffinity or microfluidic capture strategies to enhance the sensitivity of breast cancer exosome analysis. For example, immunomagnetic capture approaches using antibodies targeting tumour-associated markers such as epithelial cell adhesion molecule (EpCAM) have been used to selectively isolate tumour-derived exosomes from plasma, followed by electrochemical sensing for the detection of breast cancer-related exosomal biomarkers [119].

In addition, recent electrochemical biosensing platforms have demonstrated highly sensitive detection of breast cancer-derived exosomes in liquid biopsy applications, highlighting their potential for non-invasive cancer diagnostics [124].

### Dynamic Light Scattering (DLS)

DLS, or photon correlation spectroscopy, is widely used to measure particle size in colloidal suspensions by analysing vesicle Brownian motion [125]. Measurements are performed using laser-based instruments and analysed with software such as Zetasizer. While rapid, DLS mainly estimates hydrodynamic diameter and is affected by aggregation and polydispersity [126].

DLS has been demonstrated as a rapid and non-destructive method for assessing exosome size distribution and sample homogeneity following isolation from cell culture or biological fluids. For example, Lyu Qinyu *et al*. used DLS to characterize exosomes isolated from a human breast cancer cell line (MDA-MB-231) and a fibrosarcoma cell line, reporting mean particle radii within the expected exosome size range (~ 90–110 nm) and relatively homogeneous vesicle populations. These results support the utility of DLS for preliminary characterization of extracellular vesicles prior to more detailed structural and molecular analyses [127].

### Raman Spectroscopy

Raman spectroscopy is a label-free technique that characterizes EVs by analysing vibrational modes via inelastic laser scattering, providing molecular composition information [128]. However, conventional Raman spectroscopy has low sensitivity, requiring high sample concentrations, strong laser power, and long acquisition times, limiting its application to EV analysis [129]. These limitations can be addressed using Surface-Enhanced Raman Spectroscopy (SERS), where metal nanoparticles amplify Raman signals for improved detection. Surface-enhanced Raman spectroscopy (SERS) has been applied to the molecular characterization of breast cancer–derived exosomes, enabling detection of disease-related biochemical signatures [130]. For example, SERS analysis of exosomes isolated from breast cancer cell lines such as MCF-7 and MDA-MB-231 revealed distinct Raman spectral peaks associated with variations in protein and lipid composition, demonstrating the ability of SERS to capture molecular fingerprints of tumour-derived vesicles [131]. Furthermore, integration of SERS spectral datasets with machine-learning algorithms has enabled accurate classification of breast cancer subtypes based on exosomal signatures, supporting the potential of this technique for non-invasive cancer diagnostics [132].

### Plasmonic Exosome Detection (nPLEX)

The nPLEX sensor enables label-free detection of small EVs using nanohole arrays in a gold film that generate surface plasmons sensitive to exosome binding [133]. Individual channels can be functionalized with antibodies (e.g., EpCAM, CD24, CD63) for multiplexed protein analysis. This system allows rapid, sensitive detection and extraction of exosomes for downstream genomic and proteomic analyses, supporting diagnostic and therapy-monitoring applications [83]. Surface plasmon resonance (SPR)–based plasmonic biosensing has been applied for sensitive, label-free detection of tumour-derived exosomes. For example, Sina *et al*. developed an SPR biosensor capable of real-time detection of exosomes secreted by the BT-474 breast cancer cell line by monitoring refractive index changes upon vesicle binding to the sensor surface. The platform demonstrated high sensitivity and specificity for detecting exosomes in complex biological samples, highlighting the potential of SPR-based biosensors for breast cancer exosome analysis and liquid biopsy applications [134].

Oveall, these techniques provide complementary insights into exosome size, composition, and functionality, and their combined use is essential for reliable characterization in breast cancer research and clinical applications.

## Exosomes Sources

**Fig. 1:**
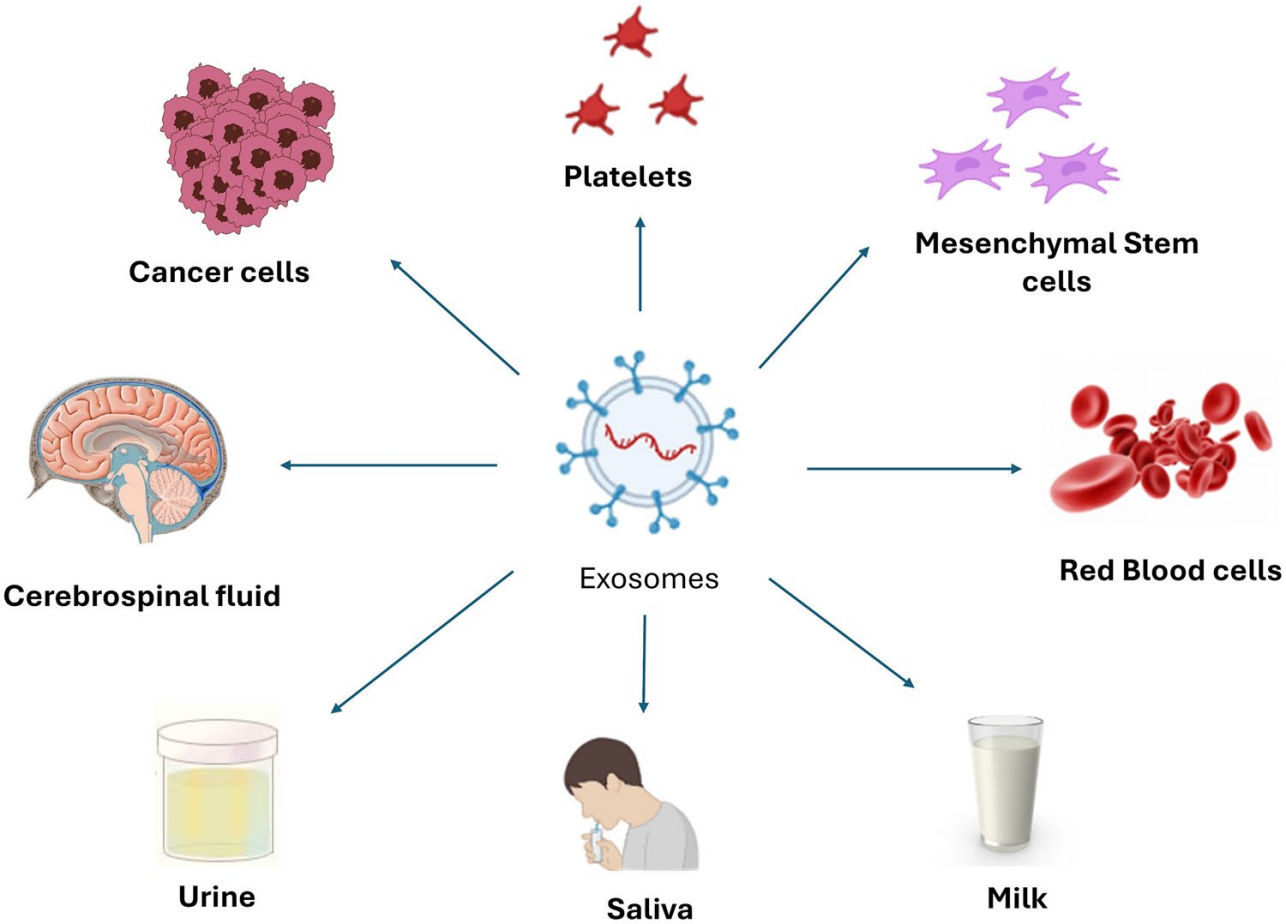
Different biological sources of exosomes that are commonly utilized for diagnostic and therapeutic applications.

As previously mentioned, Exosomes and small extracellular vesicles (sEVs) are now produced and repurposed from a very wide range of biological materials for drug-delivery applications (described in Table III). In practice, production strategies fall into two broad categories: (i) isolation of naturally secreted vesicles from biological origin (for example blood, serum, urine, saliva, milk) and (ii) collection of vesicles from conditioned media of cultured producer cells (mesenchymal stem cells, immune cells, HEK293, tumour cell lines and others) [135] as shown in Fig. 1.

**Table III:** Diverse Cellular Sources of Exosomes Used for Breast Cancer Regulation and Therapeutic Modulation

Source	Drug	Outcomes	Ref
Mammalian cell-derived exosomes			
Mesenchymal stem cells	Paclitaxel	An obvious dose-dependent reduction in the viability of aggressive MDA-MB-231 cells The superior efficacy of PTX-MSC-EMs in enhancing therapeutic outcomes over control groups underscores the role of the MSC-EM carrier	[136]
Mesenchymal stem cell	7SK noncoding RNA	Lowered proliferation, aggressiveness and tumour forming capacity in triple negative breast cancer models	[137]
Human adipocyte-derived mesenchymal stem cells	Thymoquinone	Rapid and efficient cellular uptake of FITC-labelled exosomes Tq@EXOs reduced the viability of MCF-7 cancer cell in the MTT assays	[138]
MCF7, Caco2, PC3, and HepG2 cells	Black bean phytochemicals	Enhanced bioactivity, confirming the potential use of these exosomes	[139]
4T1 cells	Epigallocatechin gallate	Exosomes reprogram their function, upon uptake by tumour-associated macrophages (TAMs) leading to a decrease in M2 polarization and overall, TAM infiltration	[140]
Autologous breast cancer cells	siRNA against S100A4	Complexation and exosome coating can improve both biodistribution and functional silencing *in vivo*	[141]
HEK293T exosome	let-7c-5p miRNA	Efficient delivery into MDA-MB-231 cells pronounced reproducible suppression of proliferation and migration	[142]
Natural killer cell	Sorafenib	Consolidation of exosomes and SFB enhanced the targeting efficacy, deducing the side effects to the normal cells and allowing continuous release of the drug	[143]
OX40L-overexpressed M1-like macrophage	No drug loaded	Immunotherapy and Microenvironment Modulation. Effective synergistic effect of innate and adaptive immunity achieving a robust anti-tumour response *in vivo* mouse	[144]
HELA derived-Exosomes DC vaccine	No drug loaded	Demonstrated potent tumour suppression in pre-clinical models of triple-negative breast cancer (TNBC)	[145]
Adipocytes and plasma	No drug loaded	Reprogram the behaviour of TNBC cells, inducing pro-metastatic processes such as epithelial-mesenchymal transition (EMT) and enhancing their overall metastatic potential, in contrast to exosomes derived from low-fat diet controls	[146]
Bacterial vesicles and other non-mammalian sources			
*H. pylori* OMVs	No drug loaded	Alterations in systemic IgA and IgG antibody levels pinpoints potential immunomodulatory effects of OMVs	[147]
E. coli (DH5 α) OMV	Chlorin e6 (Ce6) and doxorubicin	Beneficial M2-to-M1 polarization of macrophages start pyroptosis to enhance antitumor immunity	[148]
Staphylococcus aureus (Sa-EVs)	Tamoxifen	Boosted efficacy in oestrogen receptor-positive (ER⁺) breast cancer cells amplified tamoxifen-induced apoptosis	[149]
Food and plant derived exosomes			
Milk	Curcumin and resveratrol	Milk exosomes significantly improved bioavailability and potentiated the anticancer effects Exosomes bypass multidrug resistance mechanisms evading recognition and efflux by cancer cell ATP-binding cassette transporters	[150]
Milk	Raloxifene and genistein	A synergistic sensitization strategy; Genistein primed the oestrogen receptors increasing their receptivity to raloxifene. Followed by a significant amplification of the therapeutic effect and a reduction in the off-target toxicities of raloxifene	[151]
Ginger	No drug loaded	Induced apoptosis, cell cycle arrest, retarded cell migration and colony formation in triple negative breast cancer cells	[152]
Avocado	Doxorubicin	Pronounced anticancer activity MDA-MB-231 breast cancer cells	[153]
Tea flower	No drug loaded	Amplifies reactive oxygen species (ROS) inducing mitochondrial dysfunction and provoking cell cycle arrest, collectively mediating the observed anti-proliferative, anti-migratory, and anti-invasive effects against breast cancer cells *in vitro*	[154]
Tea leaves	No drug loaded	A 2.5-fold surge in intracellular reactive oxygen species Promising oral therapeutic platform for the treatment of breast cancer	[155]
Ginseng	No drug loaded	Promotes the secretion of CCL5 and CXCL9 from tumour-associated macrophages	[156]
Citrus limon	No drug loaded	Efficient cellular uptake confirmed via confocal microscopy Migration and evasion capacities of triple-negative breast cancer were evaluated via wound healing and 3D Matrigel drop evasion assays	[157]

### Mammalian Cell-derived Exosomes

Mammalian cell-derived exosomes have emerged as one of the most promising classes of naturally occurring nanocarriers for therapeutic delivery. In the context of breast cancer, mammalian cell-derived exosomes offer an especially compelling platform due to their ability to recapitulate native tumour microenvironment interactions, deliver therapeutic molecules with precision, and modulate immune and metastatic pathways [54, 158].

#### Mesenchymal Stem Cell (MSC) Exosomes

Exosomes harvested from MSCs (bone marrow, adipose tissue, umbilical cord) are among the most widely studied for therapeutic delivery because MSCs-EVs show inherent immunomodulatory properties that may be advantageous [159]. However, MSC-Exos are not biologically inert: multiple studies report context-dependent effects on tumour biology [160]. For breast cancer applications these dual actions demand rigorous potency and identity assays and, when MSCs are used as a source, validated removal or control of any pro-tumorigenic signals prior to clinical use [161]. Manufacturing recommendations emphasize standardizing MSC expansion condition, harvesting schedules and implementing closed-system microcarrier bioreactors coupled to tangential flow filtration (TFF) to support GMP-compatible scale-up [162]. A study by Kalimuthu *et al*. on exosomes from bone marrow derived mesenchymal stem cells loaded with paclitaxel (PTX) exhibited promising outcomes against breast cancer (MDA-MB-231) cells [136].

#### Immune Cell-derived Exosomes (NK, Dendritic Cells, Macrophages)

Exosomes derived from immune effector cells are promising for breast cancer therapy due to their immunomodulatory and cytotoxic properties. Natural killer (NK) cell-derived exosomes (NK-Exos) and cytotoxic T-cell EVs contain cytolytic proteins (perforin, granzymes), death-receptor ligands such as FasL, and immunomodulatory cytokines. These features enable NK-Exos to induce tumour cell apoptosis, modulate the tumour microenvironment, and deliver therapeutic cargos, including chemotherapeutics, siRNA, or ferroptosis inducers, providing a cell-free cytotoxic strategy [163–165].

Dendritic cell-derived exosomes (DC-Exos) present tumour antigens with co-stimulatory molecules (CD80, CD86) and MHC complexes, enabling T-cell priming and enhancing anti-tumour immunity, making them suitable as cell-free cancer vaccines. However, low yields and the requirement for donor-matched antigens limit large-scale production [145, 166, 167]. In addition, tumour-derived exosomes can activate DCs, promoting maturation and antigen presentation and further enhancing anti-tumour immune responses [145].

Macrophage-derived exosomes, particularly from M1-polarized macrophages, can repolarize tumour-associated M2 macrophages toward a pro-inflammatory phenotype, increasing immune infiltration and anti-tumour activity. Engineered M1-EVs carrying co-stimulatory ligands or siRNA targeting immune checkpoints (e.g., PD-L1) can modulate the tumour microenvironment and inhibit tumour progression via functional microRNA transfer [168, 169].

Despite their therapeutic promise, immune cell-derived exosomes face significant challenges for clinical translation. Their production yields are generally lower than those of MSC-derived exosomes, and variability in donor cells or immune activation states can compromise reproducibility and batch-to-batch consistency. Standardized manufacturing therefore requires careful optimization of culture conditions, activation stimuli, and robust quality-control assays to ensure consistent potency and safety [166, 167, 169, 170].

**Fig. 2:**
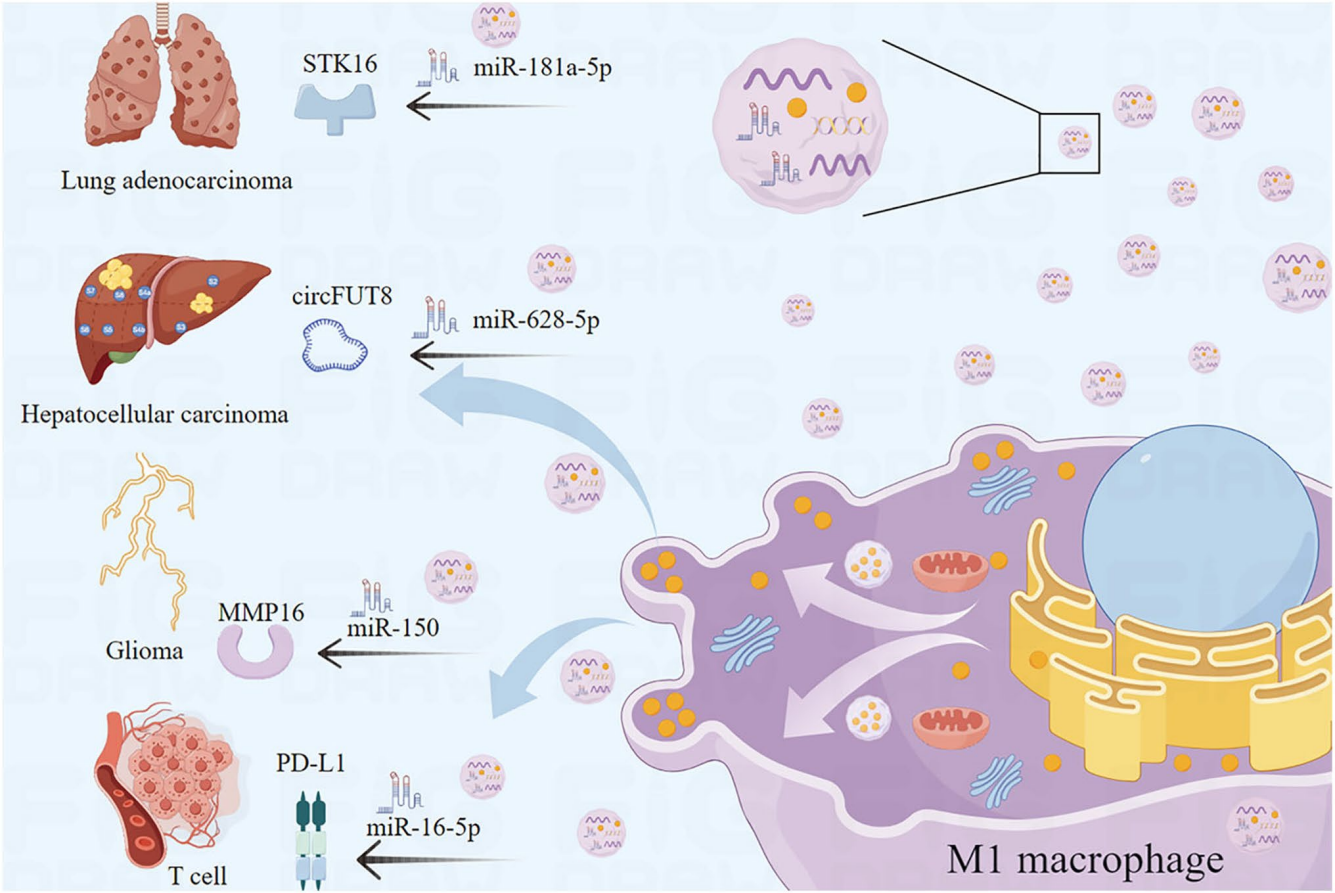
Illustration showing how M1 macrophage-derived extracellular vesicles (M1-EVs) inhibit tumour growth and progression through the intercellular transfer of microRNAs. From Zhou *et al*. [168], under the terms of CC BY 4.0 license.

Examples of therapeutic applications include the delivery of sorafenib (SFB) to breast cancer spheroids via NK-Exos, which enhanced intracellular trafficking, sustained drug release, and selective cytotoxicity [143]. DC-derived exosomes presenting tumour antigens have been tested in early clinical trials as cancer vaccines [166, 167]. Conversely, immune cell-derived exosomes (for example, M1 macrophage-derived exosomes engineered to overexpress co-stimulatory ligands or carry siRNA against PD-L1) can repolarize the tumour microenvironment and increase T-cell infiltration. As illustrated in Fig. 2, M1-EVs can delay and inhibit the progression of various cancers through the transfer of functional microRNAs that modulate tumour signaling and immune responses [168]. New immune checkpoint pathways involving macrophage-mediated innate immunity show great promise in breast cancer targeting owing to its abundance.

#### Tumour-cell-derived Exosomes (TDEs)

Tumour-derived exosomes have a natural propensity for “homotypic” uptake by parental or similar tumour cells and therefore possess an intrinsic tumour tropism that experimentalists have exploited for targeted delivery [171]. Nevertheless, TDEs frequently carry oncogenic proteins and miRNAs that promote metastasis, immune suppression and therapy resistance, and thus pose a safety concern if used directly as drug carriers without extensive re-engineering [172]. Because of these hazards, several groups favour using tumour-membrane cloaked nanovesicles or hybrid membranes to capture homotypic targeting while avoiding the transfer of oncogenic biomolecules.

Notably, recent advances in breast cancer research have provided new insights into this approach. For instance, Ni *et al*. has shown that exosomes derived from metastatic breast cancer cells can remodel the tumour microenvironment and facilitate pre-metastatic niche formation through the transfer of specific integrins and signaling molecules [173].

#### Blood-cell and Erythrocyte-derived Vesicles

Red blood cell (RBC) membrane-derived vesicles and RBC-EVs are emerging as low-immunogenic carriers with favourable circulatory lifetimes [174]. RBCs are an attractive bulk source due to their abundance and lack of a nucleus. RBC-derived vesicles have been explored for antibody and drug loading; however, validated hemocompatibility testing and robust processing are required to ensure reproducible membrane composition and removal of residual donor blood products [175]. Recent studies highlight their potential in breast cancer therapy. Romano *et al*. [135] developed Cisplatin- and Cetuximab-loaded RBC-EVs with enhanced tumour targeting and reduced systemic toxicity, demonstrating significant tumour growth inhibition and favourable safety profiles in preclinical breast cancer models [176].

### Bacterial Vesicles and Other Non-mammalian Sources

Bacterial extracellular vesicles (BEVs), including outer membrane vesicles (OMVs) from Gram-negative bacteria and membrane vesicles (MVs) from Gram-positive species, represent a rapidly expanding class of bio-nanoparticles with therapeutic potential. These vesicles (20–250 nm) are naturally released during bacterial growth and stress and mediate intercellular communication by delivering proteins, nucleic acids, lipids, and metabolites to host cells [177]. In oncology, BEVs are increasingly explored as immunostimulatory nanocarriers, drug delivery systems, and tumour-targeted immunotherapeutic platforms due to their intrinsic bioactivity and surface modifiability [178].

OMVs from Gram-negative bacteria are particularly notable for their strong immunogenicity, providing valuable adjuvant effects in vaccine and cancer immunotherapy. They have been engineered to display tumour-associated antigens or co-deliver photosensitizers and chemotherapeutics, producing synergistic antitumour effects through combined cytotoxicity and immune activation in preclinical models [179].

Recent advances demonstrate their relevance in breast cancer therapy. Sepahdar *et al*. showed that *Escherichia coli*-derived OMVs engineered to deliver PTX enhanced tumour growth inhibition and immune activation in TNBC models [180]. Similarly, Shadi Vaziri *et al*. reported that *Helicobacter pylori*-derived OMVs conjugated with photosensitizers induced potent photothermal and immune-mediated cytotoxicity against breast tumours [147].

In contrast, BEVs from Gram-positive bacteria, such as *Lactobacillus*, *Bifidobacterium*, and *Streptococcus* species, are considered safer due to the absence of lipopolysaccharides (LPS) [181]. Their relevance to breast cancer is supported by findings from An *et al*. who showed that *Staphylococcus aureus*-derived EVs (Sa-EVs) significantly enhanced tamoxifen efficacy in oestrogen receptor-positive (ER⁺) breast cancer cells by amplifying apoptosis, downregulating PI3K/AKT and ERK signalling, and increasing TNF-α expression [149].

### Food and Plant-derived Extracellular Vesicles

Food- and milk-derived exosomes have gained attention as biocompatible nanocarriers for cancer drug delivery. In breast cancer, these vesicles improve the pharmacokinetic and pharmacodynamic profiles of loaded agents and exhibit intrinsic bioactivity, providing synergistic anticancer and immunomodulatory effects that may help overcome drug resistance and tumour recurrence.

#### Milk-derived Exosomes (mEXOs)

**Fig. 3:**
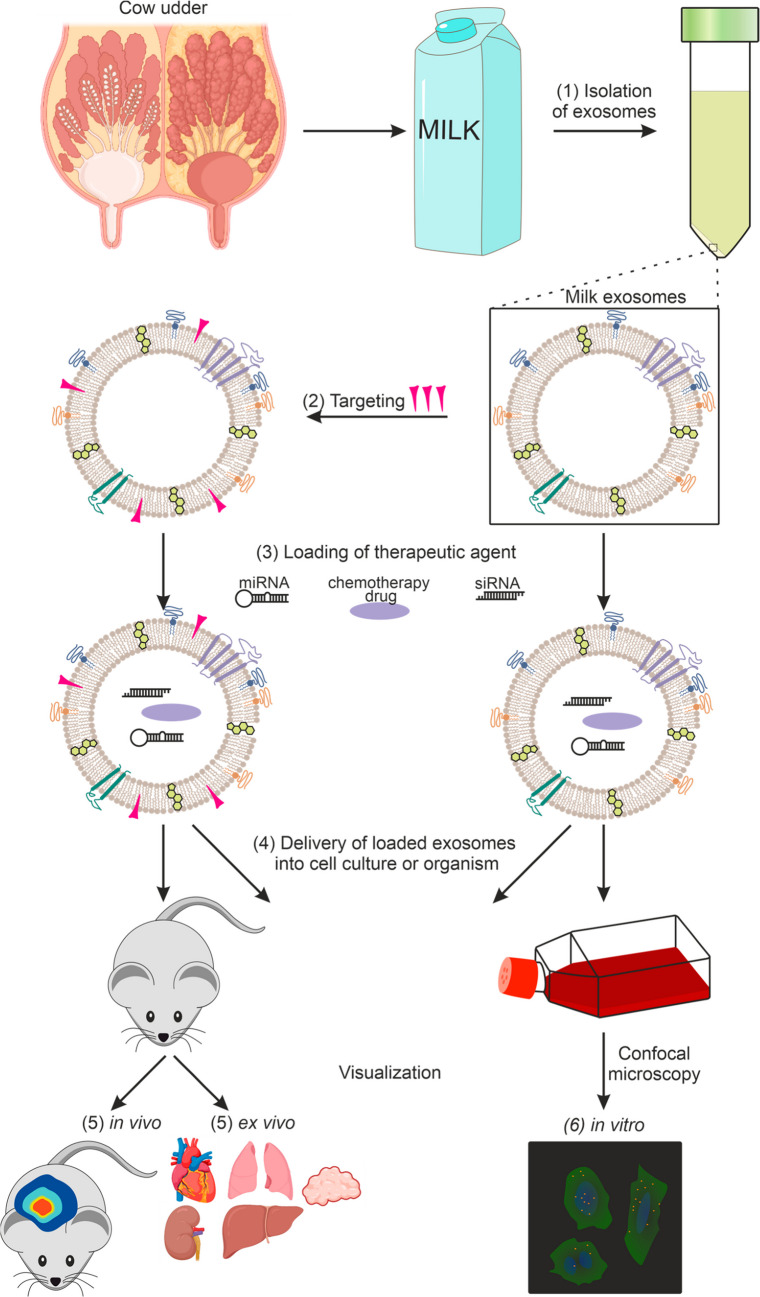
Steps for using exosomes as therapeutic carriers: (1) isolate exosomes from milk, (2) functionalize or target them, (3) load the therapeutic cargo, (4) administer to cells or organisms, (5) perform *in vivo* and ex vivo imaging, and (6) conduct *in vitro* imaging. From Timofeeva *et al*. [185], under the terms of CC BY 4.0 license.

Bovine and human milk are exceptionally rich sources of small extracellular vesicles (sEVs) and have attracted intense interest as scalable carriers for small molecules and macromolecular therapeutics. Milk-derived exosomes (mEXOs) combine several pharmaceutically attractive properties: they are naturally evolved for oral delivery (adapted to survive the gastrointestinal environment to some extent), they demonstrate cross-species biocompatibility in many models, and they can encapsulate both hydrophobic and hydrophilic cargos [182]. Additionally, their surfaces can be engineered for tissue-specific targeting, and milk serves as a scalable source ideal for therapeutics [183]. Crucially, they protect their cargo from gastrointestinal degradation, enabling oral delivery and systemic biodistribution, including to the brain [184]. A schematic overview of the workflow for using milk-derived exosomes as a delivery system, from isolation to imaging, is presented in Fig. 3.

As an example, Raloxifene (RAL) and Genistein (GEN) co-loaded milk exosomes were synthesized by Gupta *et al*. boosting their delivery in breast cancer [151]. The proposed mechanism of action for this exosome co-delivery system involves a synergistic sensitization strategy. The subsequent outcome is a dual benefit: a significant amplification of the therapeutic effect and a reduction in the off-target toxicities commonly linked to RAL monotherapy [151].

#### Plant-derived Exosome-like Nanoparticles (PDENs)

Vesicles isolated from plants have been described under many names (plant-derived extracellular vesicles, exosome-like nanoparticles or PDENs). PDENs are attractive because they are inexpensive to produce at scale, are inherently biocompatible when administered orally, and in many studies carry bioactive lipids, polyphenols and RNAs with intrinsic pharmacologic activity. Mechanistically, PDENs can be internalized by mammalian cells through lipid-dependent endocytosis and can deliver payloads that modulate oxidative stress, inflammation and cell proliferation pathways relevant to carcinogenesis [186]. Beyond their role as drug delivery vehicles, plant-derived exosomes are increasingly investigated for their inherent anticancer activity. This therapeutic potential is underpinned by a suite of advantageous biophysical properties, including their immunogenic profile, innate biocompatibility, demonstrated safety, cell-free nature, and optimal nanoscale dimensions, which collectively facilitate interactions with tumour cells. [158].

For instance, the first study to report that ginger exosomes diminishing the proliferation of triple-negative breast cancer cells showed boosted cellular uptake within 6 h of exposure [152]. Additionally, Avocado-derived exosomes, a natural drug delivery system for DOX, were synthesized and evaluated in breast cancer [153]. DOX is successfully encapsulated in avocado-derived exosomes exhibiting pronounced anticancer activity against various cancer cell lines and promotes apoptosis in MDA-MB-231 breast cancer cells confirming the potential of exosomes in delivering chemotherapeutics efficiently [153]. Citrus limon-derived extracellular nanovesicles (CLENs) were evaluated for the anticancer efficacy in triple negative breast cancer cell lines (4T1 and HCC-1806 cells) [157]. Results revealed that CLENs were internalized by both cell lines via an endocytic pathway. This efficient cellular uptake correlated with a consequent reduction in cell viability, which was observed to be dependent on both dose and exposure time. Furthermore, the anti-metastatic potential of CLENs was evaluated, demonstrating a significant impairment in the migration and evasion capacities of TNBC cells [157].

## Source-Specific Considerations

The selection of an appropriate vesicle source is a pivotal decision with significant regulatory and clinical implications. Several key factors must be carefully considered, including the intrinsic bioactivity of the native vesicles the variability among donors, potential pathogen risks, and the feasibility of producing clinical-grade batches with consistent critical quality attributes (CQAs). Additionally, the compatibility between the vesicle source’s stability and immunogenic profile is essential to ensure both safety and therapeutic efficacy [30].

Despite this considerable promise, the clinical translation of EV-based therapies faces significant challenges. Translation is constrained by practical and conceptual obstacles. Manufacturing standardization, scalable and orthogonal purification methods, validated potency assays and robust characterization of critical quality attributes are still incompletely established for most EV pipelines [187].

## The Therapeutic Potential of EV-Based Delivery Systems in Breast Cancer

Exosomes possess several features that make them particularly advantageous for breast cancer therapy. Their endogenous origin and membrane composition confer high biocompatibility and low immunogenicity, enabling safe systemic administration with minimal immune clearance [188, 189]. They also carry membrane proteins and lipids reflective of their parental cells, which provide intrinsic targeting capabilities, promoting preferential uptake by tumour cells and components of the breast cancer microenvironment [31, 190].

Additionally, the lipid bilayer protects therapeutic cargoes, including chemotherapeutics, nucleic acids, and proteins from enzymatic degradation, enhancing circulation stability, bioavailability, and tumour penetration compared with free drugs or many synthetic carriers [191, 192]. Importantly, exosomes can transport complex bioactive molecules such as siRNAs, miRNAs, and proteins, enabling gene modulation and immunotherapy strategies that are challenging to achieve with conventional nanoparticles [192, 193]. These characteristics collectively support the use of exosomes as promising delivery vehicles in breast cancer therapy.

## Payload Selection: What Breast Cancer Needs?

Breast cancer’s heterogeneity demands flexible therapies targeting proliferating cells, therapy-resistant subpopulations (including cancer stem cells), and the immunosuppressive microenvironment that drives metastasis and recurrence [194]. From a therapeutic standpoint, the selection of payloads for exosome-based drug delivery should be guided by several key considerations. First, the choice should address the specific pharmacokinetic and pharmacodynamic (PK/PD) limitations of the candidate drug, such as poor solubility, rapid systemic clearance, active efflux by ABC transporters, or limited penetration into protected tissue niches [195]. Second, the therapeutic index of the agent should be evaluated, with particular emphasis on reducing off-target toxicities through targeted delivery [196]. Finally, the payload should exhibit mechanistic compatibility with exosome biology, for instance, nucleic acids encapsulated within exosomes benefit from enhanced stability and efficient cytosolic delivery [197]. Exosomes uniquely facilitate uptake and tissue tropism, protect labile cargo, and evade some innate immune recognition due to their size and natural origin [198].

### Conventional Cytotoxic Agents

Conventional cytotoxic agents, including anthracyclines such as DOX, taxanes such as PTX and docetaxel, and platinum-based compounds, remain cornerstone treatments in both early- and advanced-stage breast cancer [199]. However, their clinical utility is often limited by significant systemic toxicities and dose-limiting adverse effects.

Encapsulation of cytotoxic agents within exosome is particularly appealing due to the intrinsic properties of exosomes, which can modify drug biodistribution, promote tumour-specific accumulation, and protect encapsulated drugs from premature metabolism, plasma protein binding, and efflux transporter recognition [9]. Preclinical studies have demonstrated that exosome-encapsulated formulations of DOX and PTX exhibit greater tumour localization and enhanced antitumor activity compared with their free drug counterparts, while concurrently reducing systemic toxicity markers in animal models [35, 200].

### Nucleic acid Therapeutics: Protection, Delivery and Biological Consequences

Nucleic acid-based therapeutics, such as small interfering RNA (siRNA), microRNA (miRNA) mimics or inhibitors, antisense oligonucleotides, and gene-editing constructs, provide highly specific strategies to suppress oncogene expression, restore tumour suppressor function, and reprogram the tumour microenvironment [201]. In breast cancer, these modalities hold particular promise for targeting key molecular drivers of malignancy, including dysregulated signaling pathways and therapy-resistant phenotypes [202]. However, their clinical translation has been hindered by major delivery challenges, such as rapid degradation by extracellular nucleases, inefficient cellular uptake, and sequestration within endo-lysosomal compartments that prevent cytosolic release and functional activity [203].

Exosomes offer a biologically inspired solution to these barriers. As natural mediators of intercellular communication, they are evolutionarily optimized to transport RNA and proteins between cells. Exosomes inherently package small RNAs and deliver them through mechanisms such as endocytosis, membrane fusion, or receptor-ligand interactions, thereby protecting their cargo from enzymatic degradation and facilitating efficient intracellular delivery [204].

In breast cancer, accumulating evidence underscores the biological relevance of exosome-mediated RNA transfer. For instance, tumour-derived exosomes enriched with oncogenic miRNAs have been shown to enhance invasion, metastasis, and epithelial-mesenchymal transition in recipient cells [205, 206]. These ongoing research studies not only highlight the potent role of exosomal RNAs in driving disease progression but also point to a compelling therapeutic opportunity, in which engineering exosomes to deliver tumour-suppressive RNAs or RNA interference agents is capable of reversing malignant phenotypes and overcoming therapeutic resistance [207].

Several mechanistic and therapeutic studies show that exosome loading of functional RNAs can alter breast cancer phenotypes *in vitro* and *in vivo*. A HEK293T exosome platform carrying let-7c-5p miRNA mimics was developed by Kim and Rhee [142]. This system achieved efficient delivery into MDA-MB-231 cells and produced reproducible suppression of proliferation and migration while modulating validated downstream targets, establishing a straightforward proof of principle that engineered exosomes can deliver tumour suppressive miRNAs to aggressive breast cancer cells [142].

More complex therapeutic constructs have also been translated to animal models. A recent study by Zhao *et al*. packaged siRNA against S100A4 into an exosome based nanocomplex formed with cationic bovine serum albumin [141]. This CBSA/siS100A4 in Exosome formulation protected siRNA from degradation, produced potent target knockdown and markedly reduced postoperative lung metastasis in murine breast cancer models [141].

Taken together, these studies illustrate two consistent messages. First, exosomes naturally contain diverse RNA cargo that tracks with tumour biology, which supports both biomarker and therapeutic applications. Second, engineered exosomes can deliver small interfering RNAs, microRNA mimics and long noncoding RNAs to breast cancer cells and can produce biologically meaningful knockdown or phenotypic reprogramming *in vitro* and *in vivo* [207].

Nonetheless, several technical challenges continue to limit clinical translation. Achieving efficient and reproducible loading of nucleic acids into exosomes remains a key hurdle, as does preventing RNA aggregation or degradation during the loading process [208] (see Loading Strategies Section). Another critical step is ensuring successful endosomal escape following cellular uptake, which is essential for the RNA payload to reach the cytosol and exert its intended gene-silencing effect. Comparative studies have shown that techniques such as sonication and certain chemical conjugation approaches may achieve higher RNA loading efficiency and preserve RNA integrity more effectively than electroporation in some experimental settings. However, each method requires careful optimization to maintain exosome structural integrity, biological activity, and functional delivery performance [209].

### Repurposed Small Molecules: Harnessing Known Safety with Targeted Delivery

Active pharmaceutical ingredients APIs repurposing presents a compelling novel strategy for oncology, particularly in breast cancer, because safety profiles, human PK, and manufacturing routes are already established. Candidate repurposed agents that have attracted attention for breast cancer include metformin, statins, itraconazole and disulfiram (Table IV). Combining repurposed small molecules with exosome delivery has two complementary advantages.

**Table IV:** Representative Repurposed Drugs & Herbal Bioactives Studied With Exosome Platforms for Breast Cancer (Selected Evidence & Translational Gaps)

Drug/Bioactive Compound	Rationale in Breast Cancer	Exosome Type/Source	Loading Method	Exosome Studies/Models	Key Translational Gap	Ref
Metformin	Modulates tumour metabolism and stemness via mitochondrial inhibition	Blood-derived exosomes	Passive co-loading with cPLA_₂_ siRNA	Crossed BBB and suppressed tumour growth in Glioblastoma Patient-Derived Xenograft (PDX) model	No breast-specific exosome studies; PK/BD data needed	[216]
Atorvastatin	Anti-angiogenic and antiproliferative statin; suppresses VEGF	Human endometrial mesenchymal stem cells (hEnMSC) exosomes	Passive loading	Induced apoptosis and inhibited angiogenesis in U87 GBM 3D model	No breast data; in-vivo validation needed	[213]
Simvastatin	Lipophilic statin; inhibits breast cancer invasion and metastasis by suppressing pituitary tumour-transforming gene 1 (PTTG1) expression, reducing MMP-2/9 activity, and downstream pro-invasive genes	–	Not exosome-based	*In vitro*: Hs578T and MDA-MB-231 cells; siRNA knockdown and PTTG1 overexpression confirmed mechanism	Preclinical only; exosome-mediated or targeted delivery not explored; *in vivo* breast cancer validation needed	[217]
	Kills radioresistant breast cancer cells; induces apoptosis and autophagy; reverses EMT and reduces migration	–	Not exosome-based	*In vitro*: radioresistant MDA-MB-231-RR, T47D-RR, Au565-RR cells	Preclinical only; no *in vivo* validation or targeted/exosome delivery explored; radio sensitization in patients untested	[218]
Itraconazole	Antiangiogenic and anti-metastatic potential	Cancer cell-derived EVs	Acts as inhibitor (not loaded)	Blocked EV cargo nuclear entry via VOR complex in colon cancer cells	No breast cancer studies; indirect evidence	[214]
Disulfiram	Inhibits vasculogenic mimicry and metastasis in TNBC	–	LIFU/MMP-2 dual-responsive nanodroplets	Ultrasound-controlled tumour-targeted release reduced VM formation and lung metastasis in TNBC models	No exosome delivery explored; potential for future studies	[219]
Orlistat	Fatty Acid Synthase (FAS) inhibitor; blocks lipid metabolism and tumour growth in LDLR/FAS-overexpressing breast cancer cells	–	LDLR-targeted lipid nanoparticles (LDLR-OTNs)	Rapid receptor-mediated uptake and cytotoxicity in BT-474, MDA-MB-453, MCF-7 cells	No exosome-based delivery explored; *in vivo* validation needed	[220]
	Inhibits FAS; potential TNBC therapy	–	PLGA-PEG nanoparticles, optionally co-loaded with antisense-miR-21	Enhanced apoptosis and reduced IC50 in MDA-MB-231 and SKBr3 TNBC cells; synergistic with DOX or miR-21 inhibition	No exosome delivery explored; clinical translation pending	[221]
Curcumin	Anti-cancer, antioxidant, anti-inflammatory; low solubility and rapid metabolism limit efficacy	Cell-derived exosomes	Encapsulation/priming	Improved stability and bioavailability; enhanced therapeutic effects *in vitro*, *in vivo*, and limited clinical studies	Requires targeted breast cancer-specific studies; standardized dosing and PK data needed	[222]
Resveratrol	Polyphenol with anticancer and antiproliferative effects; limited bioavailability	Milk-derived exosomes	Encapsulation	Dose-dependent antiproliferative effects in SH-SY5Y and U-87MG cells; protected from metabolism	Breast cancer-specific studies needed; clinical validation required	[223]
Curcumin/Resveratrol	Polyphenols with antiproliferative and pro-apoptotic effects; limited bioavailability otherwise	Milk-derived exosomes	Encapsulation	Rapid breast tissue delivery; inhibited proliferation and induced apoptosis in MCF-7 and MDA-MB-231 cells; avoided ABC transporters	Preclinical only; human studies needed	[150]
Epigallocatechin gallate (EGCG)	Anti-tumour polyphenol; modulates tumour microenvironment via TAM/M2 regulation	4T1 breast cancer cell-derived exosomes	Natural exosomes from EGCG-treated cells	Suppressed tumour growth; decreased TAM/M2 infiltration; miR-16 transfer to TAM in murine model	Preclinical only; human breast cancer studies needed	[140]
	Chemo preventive polyphenol; reduces TNBC EV-induced inflammation and senescence in stromal cells	MDA-MB-231 TNBC-derived EVs	EVs from EGCG-treated cells	Reduced inflammatory markers (CCL2, IL-1β) and senescence in hADMSCs; modulated signaling pathways	*In vitro* only, breast tissue microenvironment validation needed	[224]
Berberine	Anti-inflammatory, anti-apoptotic; potential support therapy	M2 macrophage-derived exosomes	Ultrasonic loading into M2 Exos	Polarized macrophages to M2; reduced TNF-α, IL-1β, IL-6; improved functional outcomes in SCI model	Not breast-specific; breast cancer application untested	[225]
Berberine/Doxorubicin	Berberine regulates HMGB1-TLR4 axis; Dox cytotoxic	Self-assembled nanodrug (DBNP), biomimetic 4T1 membrane coating	Co-assembly; cloaked with 4T1 cell membrane	Suppressed tumour growth and pulmonary metastasis in 4T1 orthotopic model; prolonged circulation	No exosome delivery; clinical translation pending	[226]

Several repurposed drugs have already drawn sustained interest in breast cancer research. Metformin exerts anticancer actions through AMPK activation, mTOR suppression and metabolic reprogramming of cancer stem cell populations [210]. Importantly, metformin has also been reported to modify extracellular vesicle biology, increasing exosome biogenesis and secretion in experimental systems, which both informs mechanism and suggests potential synergy or interaction when metformin is combined with exosome-based strategies [211].

Statins inhibit the mevalonate pathway and exhibit antiproliferative and anti-metastatic effects in preclinical breast cancer models by altering prenylation-dependent signaling and cell cycle regulators [212]. Exosome-mediated statin delivery is feasible and may enhance tumour targeting while limiting systemic lipid-lowering. For example, Valipour *et al*. showed that human endometrial mesenchymal stem cell-derived exosomes carrying atorvastatin inhibited angiogenesis more effectively than free drug in glioblastoma, highlighting potential applications in cancer therapy [213]. Antifungal drugs like itraconazole, with antiangiogenic and immunomodulatory activity, can also block intracellular trafficking and nuclear delivery of EV cargo [214]. Jin *et al.* demonstrated that composite nanoparticles co-loaded with itraconazole and VEGF siRNA synergistically suppressed breast tumour growth by inhibiting angiogenesis, inducing apoptosis, and reducing proliferation, while maintaining low toxicity [215].

Disulfiram (DSF) exhibits anticancer activity in breast cancer and benefits from nanoparticle encapsulation to improve tumour delivery and reduce systemic toxicity [227]. Disulfiram has been explored as a therapeutic agent in TNBC due to its ability to inhibit vasculogenic mimicry (VM) by reducing COL1 expression and suppressing pro-MMP-2 activation. Liu *et al*. developed a low-intensity focused ultrasound (LIFU) and MMP-2 dual-responsive nanoplatform (PFP@PDM-PEG) to efficiently deliver DSF deep into TNBC tumours, achieving controlled drug release, VM inhibition, and significant suppression of tumour progression and metastasis [219]. Orlistat, a fatty acid synthase inhibitor, also shows direct antitumour effects in breast cancer, including reduced proliferation, metastatic potential, and induction of lipid stress pathways, making it a promising candidate for exosome-mediated delivery [220, 221].

### Herbal Phytochemicals

A broad array of phytochemicals exhibits anti-cancer properties *in vitro* and in some *in vivo* models, but poor aqueous solubility, chemical instability, rapid metabolism, and enterohepatic clearance often limit their clinical translation (Table IV). Exosome encapsulation can mitigate many of these pharmacologic liabilities, enhancing solubility, protecting labile moieties from metabolic degradation, and improving tissue delivery [228].

One of the most promising phytochemicals is thymoquinone (Tq), a key bioactive compound isolated from black seed (*Nigella sativa*). Its anticancer efficacy is mediated through a diverse array of mechanisms encompassing induction of apoptosis and the suppression of cell proliferation, emerging evidence indicates that Tq also potently inhibits critical processes in tumour progression [229]. Furthermore, it shows impairment of cancer cell migration and invasion, as well as the modulation of key epigenetic markers, thereby presenting a multi-pronged attack against malignancy [230].

Complex botanical extracts rich in saponins and flavonoids have also been encapsulated into exosomes [231]. Flavonoids act primarily as antioxidants, neutralizing free radicals and thereby mitigating a key pathway for carcinogenesis [232]. Saponins offer a robust defence by countering oxidative stress and promoting the elimination of cancerous cells through the induction of apoptosis [233]. For instance, a saponin- and flavonoid-enriched black bean extract was loaded into exosomes [139]. Results showed that exosome delivery enhanced bioactivity, and a marked potentiation of antiproliferative activity with the exosomal delivery system outperforming conventional liposomal carriers [139].

Of the spectrum of polyphenols present in green tea, epigallocatechin-3-gallate (EGCG) has emerged as the most extensively studied and potent bioactive compound. EGCG has been demonstrated to impede the invasive and metastatic potential of cancer cells and to suppress the critical process of angiogenesis, thereby starving tumours of a necessary blood supply [234].

## Integrating Payload and Carrier

The selection of payload for exosome carriers is not only a function of the drug intrinsic activity but also of the specific physicochemical and biological challenges it presents. Lipophilic cytotoxic agents and phytochemicals that suffer dissolution and efflux problems benefit from the amphiphilic bilayer of exosomes [196]. Nucleic acids require protection from nucleases and a vehicle that uses natural uptake pathways, which are attributes intrinsic to exosomes [208]. Repurposed small molecules are well-suited for exosome-mediated delivery to mitigate systemic exposure, whereas surface-engineered cytokines, such as IL-12, can be displayed on exosomes to enhance intratumoral localization and reduce systemic toxicity [196]. In all cases, the interplay between payload chemistry, intended route of administration, and the choice of exosome source determines the optimal loading and formulation strategy [228]. Therapeutic cargo can be loaded into exosomes either during their formation in the producing cells or after the exosomes have been isolated, using various techniques listed in Table V. Choosing the right method requires balancing efficient cargo loading, maintaining exosome function and targeting, and ensuring the process can be scaled for clinical applications [235].

**Table V:** Comparative Summary of Exosome Loading Methods (Pharmaceutic View, Breast-Cancer Relevance)

Method	Ideal payloads for breast cancer	Typical encapsulation efficiency (%)	Effect on vesicle integrity	GMP/scale considerations	Notes	Ref
Incubation (passive)	Hydrophobic drugs, phytochemicals, Curcumin, Resveratrol, Paclitaxel and Docetaxel	Low (~ 1–10%)	Vesicle morphology, largely preserved	Simple and gentle; scalable with careful standardization	Preserves integrity; low loading efficiency; not effective for hydrophilic drugs/nucleotides	[150, 190, 200, 239–244]
Sonication/ultrasonication	Hydrophobic drugs (Paclitaxel, Doxorubicin); some nucleic acids (siRNA)	High (~ 20–40%)	Possible membrane disruption; depends on parameters	Moderate; process control required	Higher loading than passive; can be combined with incubation; requires cooling	[200, 242–248]
Electroporation	siRNA, miRNA; occasionally small drugs (Doxorubicin)	Moderate (~ 5–20%) variable, may decrease due to aggregation	Transient membrane pores; possible aggregation/protein loss	Moderate; buffer and voltage optimization needed	Widely used for nucleic acids; trehalose pulse media enhances stability and loading	[15, 200, 242, 244, 249–251]
Extrusion	Small molecules (Paclitaxel), some nucleic acids	Moderate (~ 20–30%), depends on cycles/pore size	Can alter size distribution and surface proteins	Moderate; scalable with controlled parameters	Produces homogeneous vesicles; useful for hybrid vesicle assembly	[136, 242–244]
freeze–thaw	Small molecules (paclitaxel, limited use for nucleic acids	Low–moderate (~ 10–20%)	Can increase vesicle size and aggregation; partial surface disruption	Simple and low-cost; limited large-scale use	Can be combined with other methods to enhance loading	[242–244, 252]
Chemical Conjugation/Chemical Induction	Targeting ligands, prodrugs	Variable (~ 20–50%, method-dependent), covalent or saponin-assisted)	Minimal disruption if optimized; risk of haemolysis at high saponin	Lower; complex chemistry; requires purification	Versatile post-loading; saponin improves loading; transfection reagents may introduce toxicity	[242, 253–255]
Endogenous (cell engineering)	miRNA, siRNA, protein therapeutics	High for genetic cargo; often > 30–50%)	Physiological incorporation during EV biogenesis preserves structure	Moderate to lower; requires validated stable cell lines	Suited for gene therapy; regulatory hurdles for GM cells; uniform cargo packaging	[242, 256–259]

* Encapsulation efficiency values are approximate ranges derived from representative literature and may vary depending on cargo type, exosome source, and experimental conditions [244]

### Pre-loading Strategies

Pre-loading by transfection or stable expression is the prevailing route for delivering nucleic acids and proteins because these cargos are packaged naturally into forming vesicles. Among pre-loading strategies is co-incubation, through which parent cells are exposed to a therapeutic agent, which passively internalizes and is subsequently packaged into EVs during biogenesis [236]. The efficiency of this process is not guaranteed, as it relies on passive transport mechanisms and is significantly influenced by the drug's characteristics, the established concentration gradient, and the innate secretory behaviour of the parent cells, often resulting in suboptimal loading yields [236]. Another method is Ultrasound-stimulated microbubbles (USMB) technique, which employs cavitation-induced shear forces to transiently disrupt exosome membrane integrity, enabling the influx of drug molecules [237]. Wang *et al*. employed a sonication-based loading method, incubating PTX with M1 macrophage-derived exosomes in a defined ratio. The mixture was subjected to sonication to transiently disrupt membrane integrity, followed by a recovery incubation at 37 °C to allow for membrane resealing and drug encapsulation [238].

### Post-loading Strategies

**Fig. 4:**
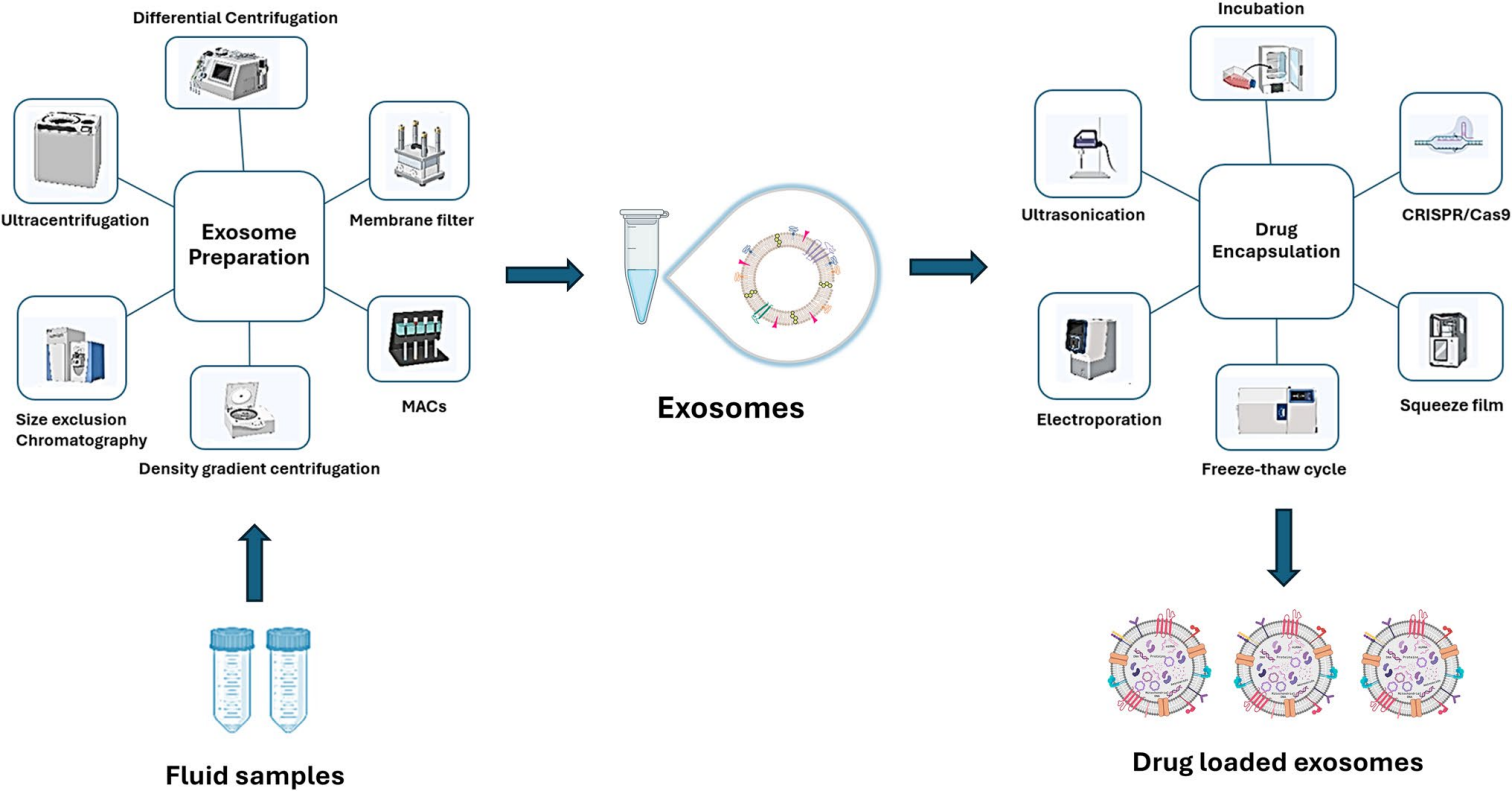
Methods for drug loading into engineered exosomes: Exogenous methods load cargo into isolated exosomes and include incubation, electroporation, sonication, freeze–thaw cycles, and extrusion.

Post-loading involves the incorporation of therapeutic payloads into isolated EVs, encompassing passive co-incubation, as well as active physical or chemical methods. Post-loading represents a highly versatile technique, distinguished from pre-loading by its superior control over the loading process and its capacity to avoid the incorporation of residual cellular debris [260]. Figure 4 illustrates the commonly used exogenous methods for loading cargo into isolated exosomes, including incubation, electroporation, sonication, freeze–thaw cycles, and extrusion. Post-loading strategies are categorized into two principal branches: passive and active loading.

Passive loading operates through the straightforward co-incubation of exosomes with a concentrated drug solution, relying on the compound's inherent ability to permeate the lipid membrane and diffuse into the vesicle interior [261]. Although valued for its straightforward protocol and minimal impact on membrane structure, the utility of this approach is constrained by two significant limitations: suboptimal loading efficiency and a narrow scope of application that is generally restricted to hydrophobic molecules [262]. Reported encapsulation efficiencies (EE%) for passive loading are typically low, commonly in the range of ~ 1–10% for small molecules and proteins, with representative values of ~ 4.9% for catalase and ~ 1.44–9.2% for paclitaxel reported in experimental studies [200, 243, 262].

Active loading strategies are employed when therapeutic cargo lacks the intrinsic ability to cross the EV membrane via passive diffusion. These methods are broadly classified into physical and chemical induction [263]. Physical induction techniques, such as electroporation, sonication, extrusion, and freeze–thaw cycles, utilize external energy to transiently disrupt membrane integrity, thereby creating temporary openings for cargo entry [200]. In contrast, chemical induction employs membrane permeabilizers to aid loading without inflicting substantial physical damage to the vesicle structure, offering an alternative pathway with potentially superior membrane preservation [264, 265].

Electroporation is widely used for loading hydrophilic molecules, particularly nucleic acids, into exosomes. In this technique, short high-voltage electrical pulses create transient nanopores in the lipid bilayer, allowing charged molecules such as siRNA or miRNA to enter the vesicle lumen before the membrane reseals [228]. **Encapsulation efficiencies for electroporation are typically reported in the range of ~ 5–20%, with representative values around ~ 5.3% reported for paclitaxel loading** [200]**. This variability is strongly influenced by buffer composition and electrical parameters, and may lead to cargo aggregation or overestimation of loading efficiency** [266, 267]**.**While electroporation can achieve relatively high loading efficiencies for nucleic acids, studies have reported potential drawbacks including RNA aggregation and partial alteration of vesicle integrity if electrical parameters are not carefully optimized [268, 269]. Despite these limitations, electroporation remains one of the most commonly applied methods for loading therapeutic RNA into extracellular vesicles for gene delivery applications [270].

Mechanical loading approaches such as sonication and extrusion improve encapsulation efficiency by temporarily disrupting or restructuring the exosomal membrane [7]. Sonication exposes vesicles to ultrasonic shear forces that increase membrane fluidity and permeability, enabling drug molecules to diffuse into the vesicle lumen before membrane recovery [271]. Sonication has been reported to achieve encapsulation efficiencies of approximately ~ 20–40%, with representative values of ~ 26.1% for catalase and ~ 28.29% for paclitaxel [200, 243]. Direct comparative studies further highlight these differences, with sonication achieving significantly higher loading efficiency (~ 28.29%) compared to electroporation (~ 5.3%) and passive incubation (~ 1.44%) when loading paclitaxel into macrophage-derived exosomes [200]. These findings consistently demonstrate superior loading performance compared to passive and electroporation methods across multiple experimental models [200, 272].

Extrusion, in contrast, forces mixtures of exosomes and therapeutic cargo through membranes with defined pore sizes, generating shear stress that reorganizes lipid bilayers and facilitates cargo incorporation [273]. Extrusion-based loading typically yields encapsulation efficiencies in the range of ~ 20–30%, with representative values around ~ 22.2% reported in comparative studies [243]. Comparative studies have shown that these approaches often achieve higher loading efficiencies than passive incubation, with extrusion and sonication demonstrating loading efficiencies exceeding those obtained through co-incubation technique [274].

Freeze–thaw loading represents a simpler physicochemical strategy in which repeated freezing and thawing cycles induce transient membrane disruption and lipid phase transitions, allowing cargo molecules to enter the vesicle lumen during membrane reformation [275]. Encapsulation efficiencies for freeze–thaw methods are generally reported in the range of ~ 10–20%, with representative values around ~ 14.7% observed in model protein loading studies [243]. Although this method is straightforward and does not require specialized instrumentation, its loading efficiency is generally lower than that achieved with sonication or extrusion, and repeated cycles may lead to vesicle aggregation or structural instability if not carefully controlled [276]. Consequently, freeze–thaw approaches are often used as complementary methods or in combination with other loading techniques to improve cargo encapsulation while preserving vesicle functionality [7, 277].

Overall, mechanical methods such as sonication and extrusion typically achieve higher loading efficiencies than electroporation or freeze–thaw approaches, although they may induce greater structural perturbation of exosomal membranes. A general hierarchy of encapsulation efficiency can be described as: passive incubation (~ 1–10%) < electroporation (~ 5–20%) < freeze–thaw (~ 10–20%) < sonication/extrusion (~ 20–40%), consistent with comparative experimental studies [200, 243, 244]. This hierarchy is widely supported across recent systematic and experimental studies evaluating loading strategies in extracellular vesicles [244, 278].

Beyond classical active methods, novel approaches such as USMB [235], microfluidic shear-based loading [279], and chemical conjugation strategies (click chemistry, lipid-anchor prodrugs) [280] are being developed to increase loading efficiency while preserving the exosomal surface landscape. USMB techniques and microfluidic devices can create brief, localized disruptions in the exosome membrane, allowing therapeutic payloads to enter while minimizing bulk heating or shear stress. Alternatively, covalent attachment of prodrugs to exosomal lipids enables controlled and sustained release. Engineered exosomes displaying surface scaffolds, can stably present proteins and present immunomodulatory activity within the tumour microenvironment [281, 282]. Each of these loading strategies must be carefully assessed for encapsulation efficiency, preservation of vesicle structure and function, and scalability before translation to clinical applications.

## Targeting Strategies: Achieving Breast Tumour Selectivity

Systemically administered exosomes display a characteristic biodistribution dominated by clearance organs and by sequestration in perivascular and reticuloendothelial compartments, which reduces the fraction available to extravasate into breast tumours. To overcome this limitation, investigators have developed multiple complementary strategies that either harness intrinsic tropisms (homotypic/homing mechanisms) or impose new, active targeting functions on the exosome surface. Each strategy carries distinct advantages and translational implications; below we review the principal approaches and the supporting evidence relevant to breast cancer as shown in Table VI.

**Table VI:** Targeting Strategies Employed in Exosome-Based Delivery for Breast Tumour Selectivity

Source	Drug	Targeting strategy	Outcomes	Ref
Active targeting moieties				
Macrophage- derived	Paclitaxel	Aminoethylanisamide-polyethylene glycol (AA-PEG) vector moiety	Engineered exosomes impose a revolutionary approach to abolish drug resistance via specific ligand-receptor binding and endocytosis	[247]
HFL-1 cells	Erastin	Folate	Suppression of the growth and spread of MDA-MB-231 cells confirmed through flow cytometry	[248]
Engineered immature dendritic cells (imDCs)	Doxorubicin	iRGD peptide	Remarkable antitumor efficacy in both *in vitro* and *in vivo* models	[35]
Human embryonic kidney cells (HEK293F)	Doxorubicin	iRGD and tLyp1	A marked enhancement in DOX internalization was observed in MCF-7 and MDA-MB-231 treated with the dual iRGD/tLyp1-functionalized exosomes, compared to non-targeted controls	[283]
Homotypic targeting				
MCF-7 cells	No drug loaded	Homotypic targeting	Marked elevation in ROS production within TNBC cells implies a direct role in reprogramming the tumour microenvironment	[284]
Tumour-cell biomimetic	Doxorubicin	Homotypic targeting	Potentiated tumour accumulation, extravasation, and parenchymal penetration	[36]
Modified-Structure exosome				
Engineered 4T1 cells	Gambogic Acid and IR780 iodide	Hybrid exosomes	Synergistic loop between of IR780 controlling the release of gambogic acid which then amplified the primary photothermal effect resulted in highly effective and targeted tumour treatment	[285]
Human plasma	Palbociclib	Hybrid exosomes	Simultaneously improved drug delivery into MCF-7 cells, ensured the therapy stayed inside the cells longer, and ultimately led to a more powerful and efficient cancer cell death	[286]
Macrophage- (RAW247.6-Exo)	Docetaxel and Bcl-2 siRNA	Hybrid exosomes	Biomimetic nanocomplex by consolidating the Bcl-2 siRNA and docetaxel efficiently targeted the tumour site and extended drug circulation time	[287]
HEK-293T human embryonic kidney cells	Indocyanine green paclitaxel sodium bicarbonate	Stimuli-responsive and externally guided systems	Synergistic chemo-sonodynamic therapy guided by real-time photoacoustic imaging via ICG, PTX and sodium bicarbonate optimizing the pH	[288]

### Active Targeting viaSurface Modification

Exosomes surface is amenable to functionalization to achieve active targeting. Engineered exosomes from donor cells transfer their cargo to recipient cells either through direct membrane fusion or via endocytosis [289]. Engineered exosomes designed to target cancer cells via specific ligand-receptor binding and endocytosis confer a revolutionary approach to abolish drug resistance [247]. Active targeting modifies the exosome outer surface to display ligands that bind receptors enriched on breast cancer cells or within the tumour microenvironment. Engineered exosomes can therefore engage receptor-mediated endocytosis pathways and increase tumour cell internalization relative to untargeted carriers. A canonical example is the Lysosomal-Associated Membrane Protein 2 (Lamp2b)-iRGD construct, where donor cells were transduced to express the iRGD peptide fused to Lamp2b, producing exosomes that preferentially accumulated in αv integrin-expressing tumours and markedly enhanced DOX delivery in preclinical models [290]. The principle has been extended to HER2-targeting fragments, folate moieties, and single-chain antibody fragments for receptor-selective delivery in breast cancer models. For instance, folate receptors overexpressed on the surface of breast cancer cells are targeted via folate labelled exosomes [248].

In another study, Tian *et al*. generated a targeted exosome delivery system with minimal immune activation [35]. They utilized engineered immature dendritic cells (imDCs) as a source. The tumour-targeting capability was engineered by transducing imDCs to express a fusion protein, particularly Lamp2b linked to the integrin-specific iRGD peptide (CRGDKGPDC). This approach ensured the efficient display of the targeting moiety on the surface of the secreted exosomes (iRGD-Exos). Subsequently, iRGD-Exos were loaded with the chemotherapeutic agent Dox via electroporation. The resultant iRGD-Exos-Dox demonstrated significantly enhanced antitumor efficacy [35].

Despite the clear efficacy gains, surface engineering may increase recognition by the innate and adaptive immune system if non-self-peptides or non-human linkers are used. Hence, it can change particle clearance kinetics and induce off-target binding in tissues that express the cognate receptor at lower levels. Importantly, the choice of conjugation chemistry and ligand density must be optimized to maintain membrane fluidity and avoid steric masking of endogenous exosomal proteins that contribute to circulation or cellular uptake.

### Homotypic Targeting (parental tumour-cell exosomes)

Exosomes have fundamentally expanded our understanding of intercellular communication within the tumour microenvironment [191, 284]. A biologically intuitive targeting tactic is to use exosomes derived from breast tumour cells themselves. Tumour-derived exosomes retain membrane proteins and glycosylation patterns that promote preferential uptake by parental and closely related tumour cells, a phenomenon sometimes termed “homing” or homotypic targeting. This property has been exploited to enhance delivery of therapeutics to breast cancer.

Critically, exosome cargoes retain biological activity upon uptake by recipient cells. For instance, exosomes derived from both primary mammary epithelial cells and breast cancer cells have been shown to elevate intracellular ROS levels. This oxidative stress triggers a DNA damage response, culminating in p53 stabilization and the subsequent maintenance of cellular homeostasis within the epithelial cell population [291]. However, the use of tumour-derived EVs raises safety concerns as mentioned previously. Consequently, translational strategies using tumour-sourced material typically incorporate additional purification and detoxification steps to remove or neutralize harmful cargo while preserving membrane-mediated targeting.

### Hybrid Exosomes

Hybridization of exosomes with synthetic liposomes or the coating of core nanoparticles with exosomal membranes represents a pragmatic compromise between biological targeting and payload control. The hybrid approach facilitates scalable manufacture because liposomal or inorganic core components are often easier to standardize and sterilize than whole-vesicle isolates. Hybrid vesicles combine the payload capacity and stimuli-responsive functionalities of synthetic liposomes with the natural surface proteins of exosomes to retain biocompatibility while improving payload loading and release control. For breast cancer, hybrids allow higher chemotherapeutic payloads with engineered release in tumour microenvironments [292]. For instance, a biocompatible tumour-cell-derived exosome-biomimetic porous silicon nanoparticles were evaluated by Yong *et al*. [36]. Intravenous administration of exosome-sheathed DOX-loaded porous silicon nanoparticles (DOX@E-PSiNPs) leads to enhanced tumour accumulation, extravasation, and parenchymal penetration [36].

**Fig. 5:**
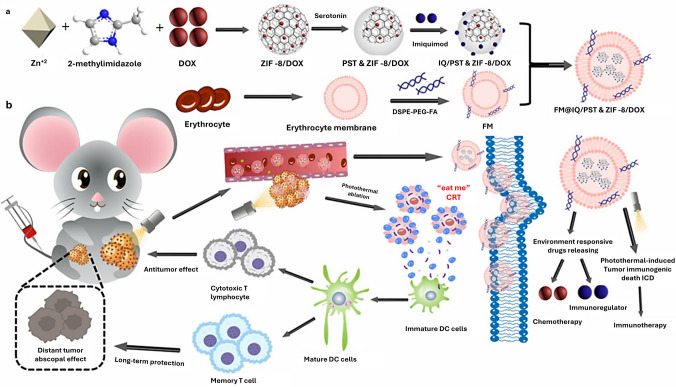
(**a**) Schematic representation of the fabrication steps for the FM@IQ/PST&ZIF-8/DOX nanoplatform (FM: folate-modified membrane; IQ: indolequinone; PST: polydopamine-silica-tannic acid composite; ZIF-8: zeolitic imidazolate framework-8; DOX: doxorubicin). (**b**) Illustration of the *in vivo* mechanism underlying the synergistic antitumor action of the FM@IQ/PST&ZIF-8/DOX system. From Wang *et al*. [293], under the terms of CC BY 4.0 license.

Another example is exosome-liposome (Exo-Lipo) hybrids. Recent studies have demonstrated that Exo-Lipo constructs can be loaded with photothermal agents, ROS-responsive prodrugs, and chemotherapeutics to achieve multimodal therapy in TNBC models. For instance, Hu *et al*. developed genetically-engineered-exosome fused with liposomes carrier (Exo-Lipo) [285]. Exo-Lipo was used to encapsulate the ROS-responsive prodrug of gambogic acid and photothermal agent IR780 iodide (IR780) to formulate an engineered-exosome multifunctional nanoplatform (IG Exo- Lipo) for TNBC therapy [285]. Upon cellular internalization, near infrared (NIR) irradiation of IR780 induced mild temperature photothermal therapy (PTT) and simultaneously generated ROS, triggering controlled release of gambogic acid. This synergistic mechanism achieved highly effective, targeted breast cancer treatment. The design and therapeutic mechanism of this engineered IG Exo-Lipo nanoplatform are illustrated in Fig. 5.

**Fig. 6:**
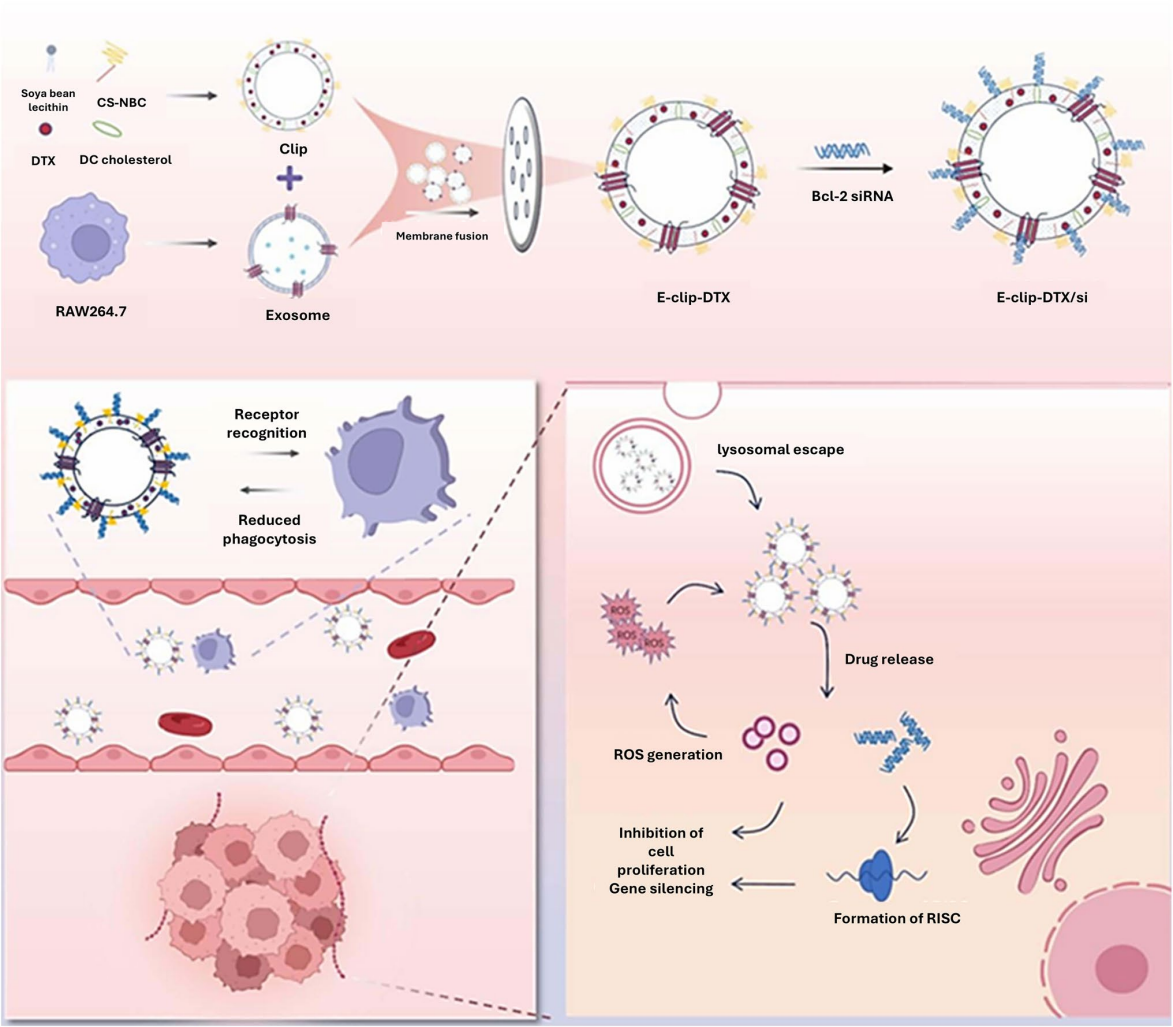
ROS-responsive biomimetic nanocomplexes combining cationic liposomes and macrophage-derived exosomes for targeted co-delivery of docetaxel and Bcl-2 siRNA in breast cancer therapy. From Xu *et al*. [287], licensed under CC BY 4.0.

Another study utilized the hybrid exosomes integrating macrophage-derived exosomes with ROS-responsive cationic liposomes (c-Lip) forming ROS-responsive biomimetic nanocomplex encapsulating both docetaxel (DTX) and Bcl-2 siRNA as therapeutics to target breast cancer [287]. After reaching the target site, these nanocomplexes respond to the abundant ROS by breaking apart and releasing their chemotherapeutic payload (DTX). This targeted release directly inhibits breast tumour cell proliferation, maximizing the drug's effect on the cancer while minimizing off-target damage (Fig. 6) [287].

The major translational questions for hybrid platforms concern membrane stability after manufacturing, retention of key exosomal proteins during the fusion process, and the immunological consequences of combining biological and synthetic moieties. Furthermore, hybridization does not completely eliminate mononuclear phagocyte system (MPS) uptake and clearance; therefore, rational surface design (e.g., low-immunogenic coatings, selective ligand display) and dosing strategies remain critical.

In addition to exosome-liposome constructs, recent studies have explored hybridization between exosomes and lipid nanoparticles (LNPs) to combine the clinically validated nucleic acid delivery capacity of LNPs with the intrinsic targeting and biocompatibility of exosomes [294]. LNPs, commonly composed of ionizable lipids, phospholipids, cholesterol, and PEG-lipids are widely used for RNA therapeutics, including mRNA vaccines. Fusion of these synthetic nanoparticles with exosomal membranes creates exosome-lipid nanoparticle hybrids (ELNs) that retain key exosomal surface markers while improving nucleic acid encapsulation and cytosolic delivery efficiency. These hybrid vesicles are typically fabricated through membrane fusion techniques such as freeze–thaw cycles, sonication, extrusion, or incubation, which facilitate lipid bilayer mixing between the LNP and exosome membranes [294]. Experimental evidence suggests that ELNs can significantly improve therapeutic nucleic acid delivery compared with conventional LNP systems. For example, Abdel-Bar *et al*. developed optimized exosome-LNP hybrids for siRNA delivery, achieving fusion efficiencies exceeding 50% while preserving key exosomal markers such as CD9, CD63, and CD81. In cancer models, these ELNs demonstrated enhanced cellular association and approximately 2.5-fold greater siRNA uptake compared with standard LNPs, resulting in effective gene silencing and improved tumour growth inhibition in murine models [295]. Another example, Son *et al*. recently developed fusogenic lipid nanoparticles capable of rapidly merging with exosomal membranes to enable efficient therapeutic cargo loading [296]. In this system, drug-loaded lipid nanoparticles were mixed with exosomes, leading to spontaneous membrane fusion within approximately 10 min and enabling the encapsulation of diverse cargos including doxorubicin, immunoglobulin G, and mRNA. Importantly, the resulting hybrid vesicles retained the intrinsic biological properties of exosomes, including their ability to traverse physiological barriers, while achieving near-complete loading efficiency for nucleic acids and other large biomolecules. Complementary studies further highlight the growing interest in integrating extracellular vesicles with lipid nanoparticle technology for RNA therapeutics and targeted drug delivery. Wang *et al*. engineered hybrid extracellular vesicles (HEVs) through low-pH–induced fusion of extracellular vesicles with lipid nanoparticles to improve mRNA loading and intracellular delivery [297]. The resulting HEVs preserved classical EV characteristics while incorporating lipid nanoparticle components capable of promoting endosomal escape. Functional studies demonstrated that these hybrids could deliver mRNA both *in vitro* and *in vivo*, with improved cellular tolerability and enhanced gene expression compared with native extracellular vesicles alone.

Recent work has also demonstrated the potential of exosome-lipid nanoparticle (EV-LNP) hybrid systems for combinational cancer immunotherapy. Beyond nucleic acid delivery, these hybrid platforms are increasingly being explored for multifunctional cancer immunotherapy applications. For instance, Sun *et al*. developed a multifunctional hybrid nanotherapeutic platform, in which tumour-derived exosomes were used to camouflage lipid nanoparticles to improve tumour targeting and immune compatibility. In this system, the LNP core was engineered to co-deliver a photothermal agent (IR806), interleukin-2 (IL-2) mRNA, and an anti-lymphocyte activation gene-3 immune checkpoint inhibitor, while the exosomal membrane provided tumour-homing capability and biological camouflage [298]. Upon near-infrared irradiation, the photothermal component induced mild hyperthermia that promoted the conversion of immunologically “cold” tumours into “hot” tumours by enhancing immune cell infiltration. Concurrently, IL-2 mRNA delivery stimulated T-cell proliferation and activation, while anti-LAG3 checkpoint blockade further enhanced antitumour immune responses. The integration of these three therapeutic modalities within the exosome-camouflaged LNP system generated a positive immunotherapeutic feedback loop that significantly improved immune checkpoint blockade efficacy in triple-negative breast cancer (TNBC) models by effectively reprogramming the immunosuppressive tumour microenvironment [298].

Collectively, these studies highlight the growing potential of exosome–lipid nanoparticle hybrid systems as versatile delivery platforms that combine the targeting capability and biocompatibility of exosomes with the high cargo-loading efficiency and scalability of lipid nanoparticles for advanced therapeutic applications.

### Stimuli-responsive and Externally Guided Systems

**Fig. 7:**
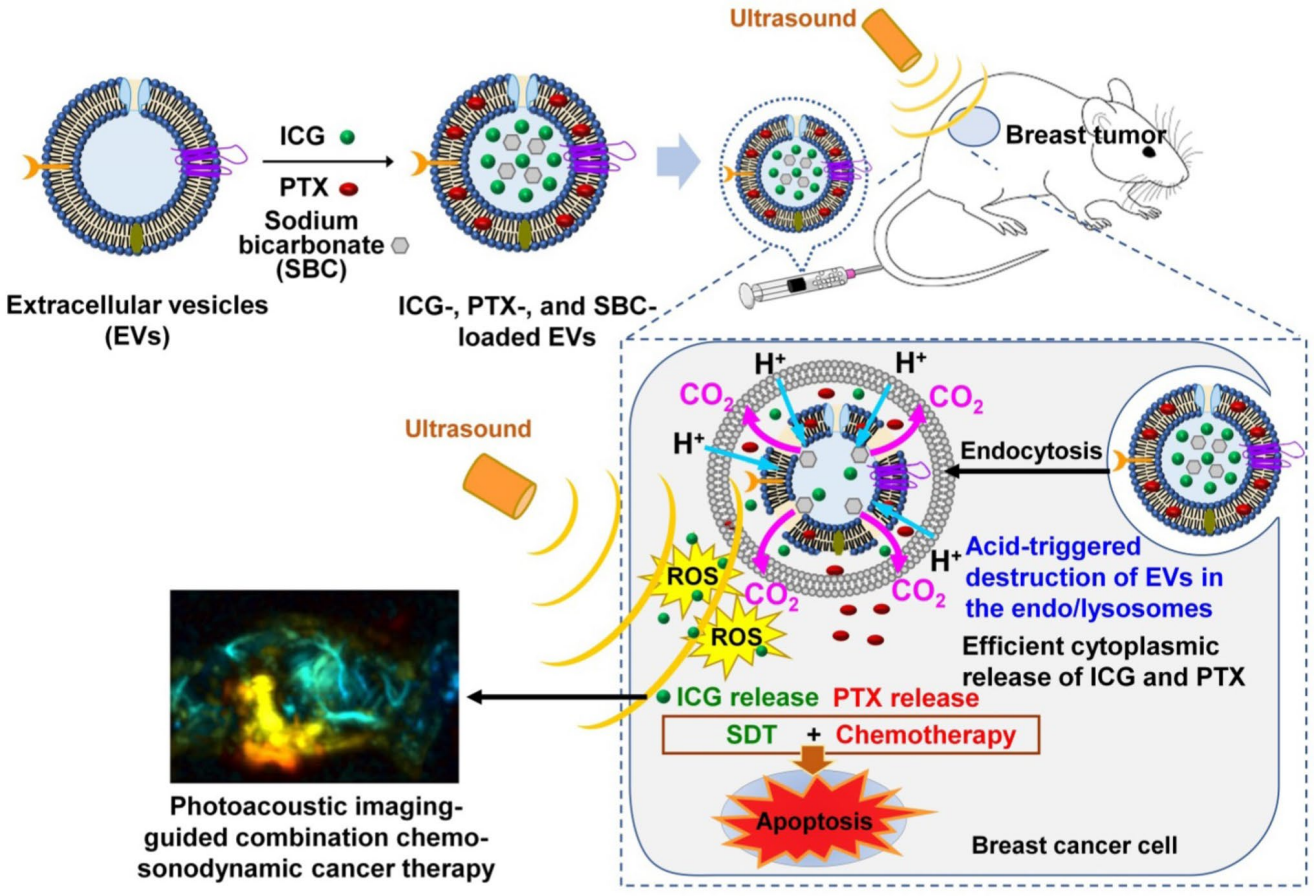
Schematic draw of sonotheranostic action of pH- and ultrasound-responsive EVs co-loaded with ICG, PTX, and SBC. From Cao *et al.* [288], under the terms of CC BY 4.0 license.

Stimuli-responsive designs incorporate molecular switches that release cargo in response to tumour microenvironmental cues (acidic pH, elevated ROS, enzymes) or external triggers (ultrasound, light, magnetic fields). Externally guided approaches, such as magnetic nanoparticle loading or focused ultrasound, allow spatiotemporal control but require integrated imaging and precise application to avoid off-target effects. These strategies enhance the functional versatility of EV therapeutics in breast cancer, particularly for locally advanced or metastatic lesions, by improving the therapeutic index. For instance, Nguyen *et al*. developed stimuli-responsive EVs to improve the specificity towards breast cancer treatment. Figure 7 illustrates the design and sonotheranostic mechanism of this system. This system co-loads indocyanine green (ICG), PTX, and sodium bicarbonate (SBC) into extracellular vesicles [288]. The formulation capitalizes on ICG's dual function as a sonosensitizer and photoacoustic imaging agent. The inclusion of SBC provides a critical pH-responsive element, allowing for tumour microenvironment-triggered activation. The engineered EVs demonstrated significantly enhanced cellular uptake of ICG. In the acidic environment of endo/lysosomes, SBC-generated CO₂ bubbles provoked membrane rupture and the initial burst release of PTX. Ultrasound (US) further amplified triggers, resulting in a powerful combinatorial chemo-sonodynamic effect that yielded high efficacy against breast cancer cells [288].

### Exosomes in Breast Cancer Theranostics

Exosomes are increasingly recognized as powerful theranostic platforms in breast cancer, combining therapeutic and diagnostic functions [31, 289]. Carrying proteins, lipids, and nucleic acids reflective of their parent cells, they serve as valuable biomarkers for early detection, disease monitoring, and assessment of therapeutic response [299, 300]. Furthermore, exosomes can be modified to incorporate imaging probes, enabling real-time tumour visualization and tracking of therapeutic delivery, establishing them as versatile platforms for integrated breast cancer theranostic [242, 301, 302].

Extensive profiling studies have highlighted the diagnostic and prognostic potential of exosome cargo in breast cancer. Exosomes encapsulate nearly half of the human proteome [303], providing a rich source for disease-specific biomarker discovery [96, 111, 114, 143, 144]. Key protein biomarkers identified in BC include EDIL3, fibronectin, Connexin-46 (Cx46), Glypican-1 (GPC-1), Glucose Transporter Type 1 GLUT-1, A Disintegrin and Metalloproteinase domain-containing protein 10 ADAM10, and Survivin-2B, which are associated with tumour progression, metastasis, and subtype stratification [110, 304–307]. Advanced liquid biopsy and exosome profiling techniques, including microfluidic chips, DNA aptamer capture, and surface-enhanced Raman scattering nanotags, allow rapid, sensitive detection of tumour markers such as EpCAM and HER2, supporting non-invasive theranostic strategies [110, 304, 305, 307, 308]. Despite these advantages, clinical translation faces challenges including vesicle heterogeneity, cargo loading efficiency, source cell selection, and administration optimization [286].

## Clinical Translation and Ongoing Preclinical and Clinical Trials

Over the past decade, exosome-based therapeutics have advanced from conceptual studies and small-scale proof-of-principle experiments to comprehensive preclinical programs and an increasing number of early clinical trials. In breast cancer models, parallel efforts in preclinical modelling and selective clinical testing have yielded valuable translational lessons regarding administration routes, biodistribution, biomarker selection, and regulatory requirements.

### Preclinical Status: Breast-cancer Models and Mechanistic Readouts

In breast cancer, exosomes have been used to deliver cytotoxic agents, nucleic acids, antisense oligonucleotides, and repurposed or natural compounds, with studies assessing effects on tumour growth, metastasis, cancer stem cells, drug resistance, and systemic toxicity. Systematic analyses show that exosome-based formulations often increase intratumoral drug accumulation, enhance antitumour efficacy *versus* free drugs, and in some cases reduce off-target toxicity. These benefits arise from efficient uptake via multiple endocytic pathways, protection of labile cargo from serum degradation, and the ability to engineer preferential delivery to breast cancer cells [190].

Representative studies underscore these translationally relevant findings. DOX-loaded exosomes derived from adipose-derived mesenchymal stem cells enhanced apoptosis and inhibited tumours cell growth while reducing systemic toxicity [309]. Folic acid-modified bovine exosomes loaded with PTX increased cellular uptake via receptor-mediated endocytosis and improved antitumor efficacy in breast cancer models [310]. Hybrid exosome-liposome constructs carrying DOX or PTX demonstrated enhanced accumulation in orthotopic mammary tumours and mitigated systemic toxicity in xenograft models [311]. Exosome-mediated delivery of siRNAs targeting MEK1 effectively silenced oncogenic pathways in triple-negative breast cancer, reducing proliferation and promoting apoptosis [312]. Similarly, exosome delivery of antisense oligonucleotides or siRNAs has reprogrammed tumours-associated macrophages or silenced resistance drivers, resulting in tumours regression in syngeneic models [15]. While these preclinical achievements are encouraging, they remain heterogeneous in design and reporting. Comparative head-to-head studies *versus* established carriers, such as liposomes or lipid nanoparticles, and comprehensive pharmacokinetic and pharmacodynamic characterization remain relatively limited.

### Clinical Trials and Real-world Translation

Clinical translation of exosome-based therapeutics has progressed to early-phase clinical trials, primarily focusing on safety, pharmacokinetics, and proof-of-mechanism. A systematic analysis indicates a rapid increase in trial registrations since 2015. However, the majority of active trials remain small and exploratory, with only a minority specifically targeting breast cancer [313].

Several exosome-based biomarkers are currently being evaluated in clinical settings, adding to the pool of existing breast cancer markers. For example, exosomes isolated from blood (NCT04288141), cerebrospinal fluid (CSF) (NCT05286684 and NCT03974204), and tumor-derived exosomes in patients receiving neoadjuvant therapy (NCT01344109) are being studied for their potential to improve diagnosis and prognosis as shown in Table VII.

**Table VII:** Selected Clinical Trials Investigating Exosomes in Breast Cancer

NCT Number	Study Status	Conditions	Interventions	Study Type	Ref
NCT01344109	Withdrawn	Breast Neoplasms		Observational	[318]
NCT05955521	Active; not recruiting	Triple Negative Breast Cancer|HER2-positive Breast Cancer	Exosome and ctDNA evaluation	Interventional	[319]
NCT05286684	Completed	Breast Cancer	Cerebral and medullary MRI, lumbar puncture, CSF sampling; biological test	Interventional	[320]
NCT04653740	Active; not recruiting	Advanced Breast Cancer	Specimen sample collection	Interventional	[321]
NCT04530890	Recruiting	Breast Cancer; Digestive Cancer; Gynecologic Cancer; Circulating Tumor DNA Exosomes	Blood samples	Interventional	[322]
NCT03974204	Withdrawn	Breast Cancer/Leptomeningeal Metastasis	Cerebrospinal fluid and blood sample collection if conclusions of initial diagnostic assessment are "confirmed", "probable" or "possible", leading to leptomeningeal metastases specific treatment	Interventional	[323]
NCT02977468	Recruiting	Triple Negative Breast Cancer	DRUG: Merck 3475 Pembrolizumab/Intraoperative radiation therapy (IORT)	Interventional	[324]
NCT04258735	Unknown	Metastatic Breast Cancer	Diagnostic test: Genomic analysis	Interventional	[325]

Recent clinical studies highlight the emerging role of exosomes as prognostic and predictive biomarkers in breast cancer. In patients with HER2-positive early breast cancer, one study is evaluating whether blood-derived exosome analysis can provide a minimally invasive alternative to tissue biopsies for treatment monitoring. Using fluorescence lifetime imaging microscopy-Förster resonance energy transfer (FLIM-FRET) to assess HER2-HER3 interactions, the study examines whether circulating exosomes reflect molecular events within tumour tissue. Serial blood samples are analysed to assess feasibility, reproducibility, and integration into clinical workflows, including implementation within the UK National Health Service (NHS), with exosomal signatures correlated to clinical and pathological responses such as residual cancer burden [314, 315].

Exosome-based diagnostics are also being explored for advanced disease monitoring and early detection. A multicentre study analysing cerebrospinal fluid (CSF) and blood-derived exosomes in patients with suspected leptomeningeal metastasis aims to improve diagnostic accuracy through proteomic and transcriptomic profiling, identify metastasis-associated molecular signatures, and monitor treatment-related temporal changes [316]. In parallel, a recent clinical study combines surface-enhanced Raman spectroscopy (SERS) with artificial intelligence to analyse extracellular vesicles across multiple cancer types, including breast cancer, enabling detection of cancer-specific signatures and tissue of origin and supporting the development of a universal, non-invasive diagnostic platform [317].

Despite these advances, breast cancer-specific interventional trials using exosome therapeutics remain scarce. Current clinical efforts predominantly focus on diagnostic and biomarker applications of tumour-derived extracellular vesicles rather than therapeutic administration, reflecting the need for stronger preclinical justification and robust, validated assays before tumour-specific efficacy trials.

### Safety, Immunogenicity and Tumour-promoting Risk

While exosomes offer strong therapeutic promise, their biological behaviour raises important safety concerns. After systemic administration, exosomes preferentially accumulate in mononuclear phagocyte system (MPS) organs, particularly the liver, spleen, and lungs, limiting tumour delivery and increasing off-target risk. Surface modifications such as PEGylation can reduce clearance but may induce anti-PEG immune responses and accelerated blood clearance upon repeat dosing, prompting the development of alternative strategies that avoid foreign polymers or exploit biomimetic “self” coatings to improve delivery while reducing immunogenicity [326].

Exosomes inherently transport bioactive cargo reflective of their cellular origin, which can exert both therapeutic and pro-tumour effects. Tumour-derived exosomes have been shown to promote organ-specific pre-metastatic niche formation through integrin-mediated organotropism and to transfer oncogenic nucleic acids and proteins that remodel the microenvironment to favour metastasis. Accordingly, safety strategies focus on stringent source selection, depletion or inactivation of unwanted cargo, and genetic or biochemical purification approaches [327].

Exosome source critically influences efficacy and safety. Mesenchymal stem cell (MSC)-derived exosomes offer scalability and clinical precedent but display context-dependent effects, with reports of both tumour suppression and pro-tumour activities, including enhanced proliferation, angiogenesis, and drug resistance driven by specific cargo. Immune-cell-derived exosomes, such as those from dendritic or natural killer cells, deliver potent effector molecules and may combine immunotherapeutic activity with acceptable immunogenicity, although scalable and well-controlled manufacturing remains challenging [328].

Regulatory translation requires clear definition of critical quality attributes, validated identity and mechanism-relevant potency assays, and rigorous control of source, isolation, and functional characterization, as emphasized by guidance from the International Society for Extracellular Vesicles (ISEV) and recent consensus statements. Early regulatory engagement is essential to align on release criteria, sterility, viral safety, and clinical pharmacology requirements, which remain central to advancing exosome therapeutics into definitive efficacy trials [329].

## Final Remarks and Future Perspectives

Exosome-based therapeutics represent a compelling convergence of cell biology and pharmaceutical science that has the potential to reshape how breast cancer is treated and, ultimately, how drug delivery paradigms are defined. The most immediate real-world impact of advances discussed in this field lies in improving therapeutic effectiveness while reducing systemic toxicity. By leveraging endogenous vesicular transport mechanisms, exosomes can enhance intratumoral drug accumulation, stabilize labile nucleic acids, and enable combination strategies, such as chemo-immunotherapy or chemo-sensitization that are difficult to achieve with conventional synthetic carriers. If successfully translated, these properties could influence treatment guidelines by supporting lower dosing regimens, reducing off-target adverse effects, and enabling personalized or biomarker-guided therapeutic strategies, particularly in treatment-resistant or aggressive breast cancer subtypes.

From a health-economics perspective, exosome therapeutics could initially increase manufacturing and development costs due to biological complexity and quality-control requirements. However, these costs may be offset by improved treatment durability, reduced hospitalization from toxicity, and the repurposing of off-patent drugs and phytochemicals with known safety profiles. Importantly, many proposed exosome platforms do not aim to replace existing therapies but rather to enhance their performance, making gradual and realistic integration into clinical practice feasible. Nonetheless, adoption into routine oncology practice will remain limited until reproducible manufacturing standards, validated potency assays, and robust pharmacokinetic and biodistribution data are established and accepted by regulatory authorities.

At present, several critical challenges prevent widespread clinical implementation. Foremost among these is the lack of standardization across exosome source selection, isolation, purification, and characterization. Variability in vesicle size, surface composition, RNA and protein cargo, and functional behaviour significantly complicates cross-study comparisons and undermines regulatory confidence. Unlike synthetic nanocarriers, exosomes are inherently heterogeneous, and this biological variability challenges the traditional pharmaceutical framework that relies on tightly controlled composition and batch-to-batch consistency. Without harmonized workflows and clearly defined critical quality attributes, exosome therapeutics risk remaining confined to proof-of-concept studies rather than progressing toward clinical maturity.

Loading strategies present another unresolved bottleneck. Current approaches, ranging from passive incubation to electroporation and membrane disruption, often involve trade-offs between encapsulation efficiency, vesicle integrity, scalability, and reproducibility. In the absence of consensus methodologies, it remains difficult to compare therapeutic outcomes across studies or to identify best-in-class platforms for specific payloads. Furthermore, pharmacokinetic and biodistribution data are frequently incomplete or inconsistent. Systemically administered exosomes face rapid clearance by the mononuclear phagocyte system, while orally administered vesicles, such as those derived from milk or plants, raise unanswered questions regarding absorption mechanisms, inter-individual variability, and dose predictability.

Safety considerations represent perhaps the most significant translational barrier. Exosomes derived from tumour cells carry a theoretical and, in some contexts, demonstrated risk of transferring pro-tumorigenic signals. Even non-tumour-derived vesicles may induce immunogenicity when surface-modified or repeatedly administered, as seen with anti-PEG antibody formation in other nanomedicine platforms. These risks necessitate rigorous safety evaluation, including long-term immunogenicity studies, biodistribution analysis, and functional assessment of unintended biological effects—requirements that have not yet been consistently met across the field.

Despite these challenges, the potential of further research in exosome therapeutics remains substantial. The ultimate goal is not merely to develop another drug carrier, but to establish a modular, biologically intelligent delivery platform capable of adapting to diverse therapeutic needs in breast cancer. Near-term opportunities are particularly strong for repurposed small molecules, phytochemicals, and nucleic acid therapeutics, where exosomes address well-recognized delivery limitations rather than introducing entirely new pharmacological entities.

Whether the future of breast cancer research lies predominantly in exosome therapeutics depends on how effectively the field addresses its foundational limitations. Alternative and potentially competing approaches, such as synthetic lipid nanoparticles, antibody–drug conjugates, and cell-based therapies continue to advance rapidly and benefit from clearer regulatory precedents. However, exosomes occupy a unique niche at the interface of these technologies, combining biological targeting with nanoscale engineering. Rather than being displaced, exosomes are more likely to evolve alongside these platforms, particularly in combination strategies or niche indications where biological delivery offers clear advantages.

Looking ahead five to ten years, the field is likely to undergo a shift from exploratory heterogeneity toward disciplined standardization. The current norm, characterized by diverse isolation methods, inconsistent reporting, and variable biological interpretation will likely give way to a smaller number of validated production platforms, standardized reference materials, and clinically relevant potency assays. In this future landscape, exosome therapeutics may not be universally applied across all breast cancer patients but selectively deployed where their unique properties justify added complexity. What the field may lose is some of its early experimental flexibility; what it will gain is translational credibility and clinical relevance.

In conclusion, exosome therapeutics for breast cancer represent a scientifically sound yet demanding opportunity. The promise is real, but realization will depend on integrating biological insight with pharmaceutical discipline, embracing transparency in safety evaluation, and designing translational studies that answer clinically meaningful questions. If these conditions are met, exosomes could emerge as a versatile and impactful component of next-generation precision oncology rather than remaining an elegant but underutilized concept.

## Data Availability

This work did not include any datasets or data to be shared.

## References

[1] Kim J, Harper A, McCormack V, Sung H, Houssami N, Morgan E, *et al*. Global patterns and trends in breast cancer incidence and mortality across 185 countries. Nat Med. 2025;31:1154–62. 10.1038/s41591-025-03502-3. 39994475 10.1038/s41591-025-03502-3

[2] Breast cancer cases and deaths are projected to rise globally [Internet]. [cited 2025 Dec 25]. https://www.iarc.who.int/news-events/breast-cancer-cases-and-deaths-are-projected-to-rise-globally. Accessed 25 Dec 2025

[3] Zagami P, Carey LA. Triple negative breast cancer: pitfalls and progress. npj Breast Cancer Nature Publishing Group. 2022;8:95. 10.1038/s41523-022-00468-0. 10.1038/s41523-022-00468-0 PMC9392735 35987766

[4] Kim E-S. Molecular targets and therapies associated with poor prognosis of triple-negative breast cancer (Review). Int J Oncol. 2025;66:52. 10.3892/ijo.2025.5758. 40444482 10.3892/ijo.2025.5758 PMC12118953

[5] Yu J, Mu Q, Fung M, Xu X, Zhu L, Ho RJ. Challenges and opportunities in metastatic breast cancer treatments: nano-drug combinations delivered preferentially to metastatic cells may enhance therapeutic response. Pharmacol Ther. 2022;236:108108. 10.1016/j.pharmthera.2022.108108. 34999182 10.1016/j.pharmthera.2022.108108 PMC9256851

[6] Obidiro O, Battogtokh G, Akala EO. Triple negative breast cancer treatment options and limitations: future outlook. Pharmaceutics. 2023;15:1796. 10.3390/pharmaceutics15071796. 37513983 10.3390/pharmaceutics15071796 PMC10384267

[7] Koh HB, Kim HJ, Kang S-W, Yoo T-H. Exosome-based drug delivery: translation from bench to clinic. Pharmaceutics. 2023;15:2042. 10.3390/pharmaceutics15082042. 37631256 10.3390/pharmaceutics15082042 PMC10459753

[8] Dikici E, Önal Acet B, Gül D, Kummer N, Stauber RH, Odabaşı M, *et al*. Bringing exosomes into the game: Current situation, opportunities, limitations and future perspectives. Materials Today Advances. 2025;28:100623. 10.1016/j.mtadv.2025.100623.

[9] Li J, Wang J, Chen Z. Emerging role of exosomes in cancer therapy: progress and challenges. Mol Cancer. 2025;24:13. 10.1186/s12943-024-02215-4. 39806451 10.1186/s12943-024-02215-4 PMC11727182

[10] Serrano DR, Juste F, Anaya BJ, Ramirez BI, Sánchez-Guirales SA, Quispillo JM, *et al*. Exosome-based drug delivery: a next-generation platform for cancer, infection, neurological and immunological diseases, gene therapy and regenerative medicine. Pharmaceutics. 2025. 10.3390/pharmaceutics17101336. 41155971 10.3390/pharmaceutics17101336 PMC12567338

[11] Bahreyni A, Mohamud Y, Ashraf Nouhegar S, Zhang J, Luo H. Synergistic viro-chemoimmunotherapy in breast cancer enabled by bioengineered immunostimulatory exosomes and dual-targeted Coxsackievirus B3. ACS Nano. 2024;18:4241–55. 10.1021/acsnano.3c09491. 38278522 10.1021/acsnano.3c09491 PMC10851665

[12] Yang Q, Li S, Ou H, Zhang Y, Zhu G, Li S, *et al*. Exosome-based delivery strategies for tumor therapy: an update on modification, loading, and clinical application. J Nanobiotechnol. 2024;22:41. 10.1186/s12951-024-02298-7. 10.1186/s12951-024-02298-7 PMC10823703 38281957

[13] Haraszti RA, Miller R, Stoppato M, Sere YY, Coles A, Didiot M-C, *et al*. Exosomes produced from 3D cultures of MSCs by tangential flow filtration show higher yield and improved activity. Mol Ther. 2018;26:2838–47. 10.1016/j.ymthe.2018.09.015. 30341012 10.1016/j.ymthe.2018.09.015 PMC6277553

[14] Sun WY, Lee D-S, Park JH, Kim O-H, Choi HJ, Kim S-J. Utilizing miR-34a-loaded HER2-targeting exosomes to improve breast cancer treatment: insights from an animal model. J Breast Cancer. 2025;28:139–57. 10.4048/jbc.2024.0262. 40133988 10.4048/jbc.2024.0262 PMC12230292

[15] Limoni SK, Moghadam MF, Moazzeni SM, Gomari H, Salimi F. Engineered exosomes for targeted transfer of siRNA to HER2 positive breast cancer cells. Appl Biochem Biotechnol. 2019;187:352–64. 10.1007/s12010-018-2813-4. 29951961 10.1007/s12010-018-2813-4

[16] Yi X, Chen J, Huang D, Feng S, Yang T, Li Z, *et al*. Current perspectives on clinical use of exosomes as novel biomarkers for cancer diagnosis. Front Oncol. 2022;12:966981. 10.3389/fonc.2022.966981. 36119470 10.3389/fonc.2022.966981 PMC9472136

[17] Rezaie J, Feghhi M, Etemadi T. A review on exosomes application in clinical trials: perspective, questions, and challenges. Cell Commun Signal. 2022;20:145. 10.1186/s12964-022-00959-4. 36123730 10.1186/s12964-022-00959-4 PMC9483361

[18] Wang C, Feng Y, Rong X, Yan J, Lv B, Jiang H, *et al*. Mesenchymal stromal cell exosomes for drug delivery of prostate cancer treatments: a review. Stem Cell Res Ther. 2025;16:18. 10.1186/s13287-025-04133-8. 39849570 10.1186/s13287-025-04133-8 PMC11755940

[19] Chen TS, Arslan F, Yin Y, Tan SS, Lai RC, Choo ABH, *et al*. Enabling a robust scalable manufacturing process for therapeutic exosomes through oncogenic immortalization of human ESC-derived MSCs. J Transl Med. 2011. 10.1186/1479-5876-9-47. 21513579 10.1186/1479-5876-9-47 PMC3100248

[20] Luo S, Chen J, Xu F, Chen H, Li Y, Li W. Dendritic cell-derived exosomes in cancer immunotherapy. Pharmaceutics. 2023;15:2070. 10.3390/pharmaceutics15082070. 37631284 10.3390/pharmaceutics15082070 PMC10457773

[21] Chen X, Tian B, Wang Y, Zheng J, Kang X. Potential and challenges of utilizing exosomes in osteoarthritis therapy (Review). Int J Mol Med. 2025;55:43. 10.3892/ijmm.2025.5484. 39791222 10.3892/ijmm.2025.5484 PMC11759586

[22] Yadav S, Maity P, Kapat K, Yadav S, Maity P, Kapat K. The opportunities and challenges of mesenchymal stem cells-derived exosomes in theranostics and regenerative medicine. Cells. 2024. 10.3390/cells13231956. 39682706 10.3390/cells13231956 PMC11640604

[23] Cai H, Huang L-Y, Hong R, Song J-X, Guo X-J, Zhou W, *et al*. *Momordica charantia* exosome-like nanoparticles exert neuroprotective effects against ischemic brain injury via inhibiting matrix metalloproteinase 9 and activating the AKT/GSK3β signaling pathway. Front Pharmacol. 2022. 10.3389/fphar.2022.908830. 35814200 10.3389/fphar.2022.908830 PMC9263912

[24] Bei HP, Hung PM, Yeung HL, Wang S, Zhao X. Bone‐a‐petite: engineering exosomes towards bone, osteochondral, and cartilage repair. Small. 2021. 10.1002/smll.202101741. 34288410 10.1002/smll.202101741

[25] Chen Y, Qi W, Wang Z, Niu F, Chen Y, Qi W, *et al*. Exosome source matters: a comprehensive review from the perspective of diverse cellular origins. Pharmaceutics. 2025. 10.3390/pharmaceutics17020147. 40006514 10.3390/pharmaceutics17020147 PMC11858990

[26] Sha A, Luo Y, Xiao W, He J, Chen X, Xiong Z, *et al*. Plant-derived exosome-like nanoparticles: a comprehensive overview of their composition, biogenesis, isolation, and biological applications. Int J Mol Sci. 2024;25:12092. 10.3390/ijms252212092. 39596159 10.3390/ijms252212092 PMC11593521

[27] Jabłońska M, Sawicki T, Żulewska J, Staniewska K, Łobacz A, Przybyłowicz KE. The role of bovine milk-derived exosomes in human health and disease. Molecules. 2024;29:5835. 10.3390/molecules29245835. 39769923 10.3390/molecules29245835 PMC11728725

[28] Gimona M, Brizzi MF, Choo ABH, Dominici M, Davidson SM, Grillari J, *et al*. Critical considerations for the development of potency tests for therapeutic applications of mesenchymal stromal cell-derived small extracellular vesicles. Cytotherapy. 2021;23:373–80. 10.1016/j.jcyt.2021.01.001. 33934807 10.1016/j.jcyt.2021.01.001

[29] Wiklander OPB, Brennan MÁ, Lötvall J, Breakefield XO, El Andaloussi S. Advances in therapeutic applications of extracellular vesicles. Sci Transl Med. 2019;11:eaav8521. 10.1126/scitranslmed.aav8521. 31092696 10.1126/scitranslmed.aav8521 PMC7104415

[30] Lener T, Gimona M, Aigner L, Börger V, Buzas E, Camussi G, *et al*. Applying extracellular vesicles based therapeutics in clinical trials - an ISEV position paper. J Extracell Vesicles. 2015;4:30087. 10.3402/jev.v4.30087. 26725829 10.3402/jev.v4.30087 PMC4698466

[31] Kalluri R, LeBleu VS. The biology, function, and biomedical applications of exosomes. Science. 2020;367:eaau6977. 10.1126/science.aau6977. 32029601 10.1126/science.aau6977 PMC7717626

[32] Sheykhhasan M, Kalhor N, Sheikholeslami A, Dolati M, Amini E, Fazaeli H. Exosomes of mesenchymal stem cells as a proper vehicle for transfecting miR-145 into the breast cancer cell line and its effect on metastasis. Biomed Res Int. 2021;2021:5516078. 10.1155/2021/5516078. 34307654 10.1155/2021/5516078 PMC8263260

[33] Hosseini M, Ezzeddini R, Hashemi SM, Soudi S, Salek Farrokhi A. Enhanced anti-tumor efficacy of S3I-201 in breast cancer mouse model through Wharton jelly- exosome. Cancer Cell Int. 2024;24:318. 10.1186/s12935-024-03501-3. 39294673 10.1186/s12935-024-03501-3 PMC11409531

[34] Ramezani R, Mohammadian M, Hosseini ES, Zare M. The effect of bovine milk lactoferrin-loaded exosomes (exoLF) on human MDA-MB-231 breast cancer cell line. BMC Complement Med Ther. 2023;23:228. 10.1186/s12906-023-04045-1. 37422619 10.1186/s12906-023-04045-1 PMC10329373

[35] Tian Y, Li S, Song J, Ji T, Zhu M, Anderson GJ, *et al*. A doxorubicin delivery platform using engineered natural membrane vesicle exosomes for targeted tumor therapy. Biomaterials. 2014;35:2383–90. 10.1016/j.biomaterials.2013.11.083. 10.1016/j.biomaterials.2013.11.083 24345736

[36] Yong T, Zhang X, Bie N, Zhang H, Zhang X, Li F, *et al*. Tumor exosome-based nanoparticles are efficient drug carriers for chemotherapy. Nature Communications [Internet]. Springer Science and Business Media LLC; 2019;10. 10.1038/s41467-019-11718-4 10.1038/s41467-019-11718-4 PMC6707218 31444335

[37] Zhang Y, Bi J, Huang J, Tang Y, Du S, Li P. Exosome: a review of its classification, isolation techniques, storage, diagnostic and targeted therapy applications. Int J Nanomedicine. 2020;15:6917–34. 10.2147/IJN.S264498. 33061359 10.2147/IJN.S264498 PMC7519827

[38] Tian Y, Gong M, Hu Y, Liu H, Zhang W, Zhang M, *et al*. Quality and efficiency assessment of six extracellular vesicle isolation methods by nano-flow cytometry. J Extracell Vesicles. 2019;9:1697028. 10.1080/20013078.2019.1697028. 31839906 10.1080/20013078.2019.1697028 PMC6896440

[39] Iwai K, Minamisawa T, Suga K, Yajima Y, Shiba K. Isolation of human salivary extracellular vesicles by iodixanol density gradient ultracentrifugation and their characterizations. J Extracell Vesicles. 2016;5:10.3402/jev.v5.30829. 10.3402/jev.v5.30829. 10.3402/jev.v5.30829 PMC4871899 27193612

[40] Hoshino A, Costa-Silva B, Shen T-L, Rodrigues G, Hashimoto A, Tesic Mark M, *et a*l. Tumour exosome integrins determine organotropic metastasis. Nature. 2015;527:329–35. 10.1038/nature15756. 10.1038/nature15756 PMC4788391 26524530

[41] Liew YW, Goh JX, Lim JS, Hamzah S, Daniel Looi QH, Lim MN, *et al*. Optimizing purification techniques and evaluating cytotoxicity of NK-92-derived small extracellular vesicles for sEVs-Liposome hybrid development in drug delivery. Journal of Drug Delivery Science and Technology. 2025;112:107263. 10.1016/j.jddst.2025.107263.

[42] Busatto S, Vilanilam G, Ticer T, Lin W-L, Dickson DW, Shapiro S, *et al*. Tangential flow filtration for highly efficient concentration of extracellular vesicles from large volumes of fluid. Cells. 2018;7:273. 10.3390/cells7120273. 30558352 10.3390/cells7120273 PMC6315734

[43] Visan KS, Lobb RJ, Ham S, Lima LG, Palma C, Edna CPZ, *et al*. Comparative analysis of tangential flow filtration and ultracentrifugation, both combined with subsequent size exclusion chromatography, for the isolation of small extracellular vesicles. J Extracell Vesicles. 2022;11:12266. 10.1002/jev2.12266. 36124834 10.1002/jev2.12266 PMC9486818

[44] Liangsupree T, Multia E, Riekkola M-L. Modern isolation and separation techniques for extracellular vesicles. J Chromatogr A. 2021;1636:461773. 10.1016/j.chroma.2020.461773. 33316564 10.1016/j.chroma.2020.461773

[45] Malik SZA, Muhilan Y, Nordin F, Ng MH, Law JX, Imran SAM, *et a*l. Stem cell derived exosome trilogy: an epic comparison of human MSCs, ESCs and iPSCs. Stem Cell Res Ther. 2025;16:318. 10.1186/s13287-025-04440-0. 40551257 10.1186/s13287-025-04440-0 PMC12186388

[46] Gheinani AH, Vögeli M, Baumgartner U, Vassella E, Draeger A, Burkhard FC, *et al*. Improved isolation strategies to increase the yield and purity of human urinary exosomes for biomarker discovery. Sci Rep. 2018;8:3945. 10.1038/s41598-018-22142-x. 29500443 10.1038/s41598-018-22142-x PMC5834546

[47] Baranyai T, Herczeg K, Onódi Z, Voszka I, Módos K, Marton N, *et al*. Isolation of exosomes from blood plasma: qualitative and quantitative comparison of ultracentrifugation and size exclusion chromatography methods. PLoS One. 2015;10:e0145686. 10.1371/journal.pone.0145686. 10.1371/journal.pone.0145686 PMC4686892 26690353

[48] Mitchell MI, Ma J, Carter CL, Loudig O. Circulating exosome cargoes contain functionally diverse cancer biomarkers: from biogenesis and function to purification and potential translational utility. Cancers (Basel). 2022;14:3350. 10.3390/cancers14143350. 35884411 10.3390/cancers14143350 PMC9318395

[49] Abhange K, Makler A, Wen Y, Ramnauth N, Mao W, Asghar W, *et al*. Small extracellular vesicles in cancer. Bioact Mater. 2021;6:3705–43. 10.1016/j.bioactmat.2021.03.015. 33898874 10.1016/j.bioactmat.2021.03.015 PMC8056276

[50] Fang S, Tian H, Li X, Jin D, Li X, Kong J, *et al*. Clinical application of a microfluidic chip for immunocapture and quantification of circulating exosomes to assist breast cancer diagnosis and molecular classification. PLoS One. 2017;12:e0175050. 10.1371/journal.pone.0175050. 28369094 10.1371/journal.pone.0175050 PMC5378374

[51] Gab-Allah MA, Jeongkwon K. Polyethylene glycol (PEG)-based precipitation for exosome enrichment: a review on recent developments, current challenges, and future perspectives. Anal Sci Technol. 2025;38:42–64. 10.5806/AST.2025.38.2.42.

[52] Jung HH, Kim J-Y, Lim JE, Im Y-H. Cytokine profiling in serum-derived exosomes isolated by different methods. Sci Rep. 2020;10:14069. 10.1038/s41598-020-70584-z. 32826923 10.1038/s41598-020-70584-z PMC7442638

[53] Su W, Li H, Chen W, Qin J. Microfluidic strategies for label-free exosomes isolation and analysis. TrAC Trends Anal Chem. 2019;118:686–98. 10.1016/j.trac.2019.06.037.

[54] Gao J, Li A, Hu J, Feng L, Liu L, Shen Z. Recent developments in isolating methods for exosomes. Front Bioeng Biotechnol. 2023;10:1100892. 10.3389/fbioe.2022.1100892. 36714629 10.3389/fbioe.2022.1100892 PMC9879965

[55] Wu M, Ouyang Y, Wang Z, Zhang R, Huang P-H, Chen C, *et al*. Isolation of exosomes from whole blood by integrating acoustics and microfluidics. Proc Natl Acad Sci USA. 2017;114:10584–9. 10.1073/pnas.1709210114. 28923936 10.1073/pnas.1709210114 PMC5635903

[56] Le M-CN, Fan ZH. Exosome isolation using nanostructures and microfluidic devices. Biomed Mater. 2021;16:022005. 10.1088/1748-605X/abde70. 33477118 10.1088/1748-605X/abde70 PMC8082697

[57] Fang H, Liu M, Jiang W. Nickel-doped microfluidic chip for rapid and efficient immunomagnetic separation and detection of breast cancer cell-derived exosomes. Appl Biochem Biotechnol. 2023;195:3109–21. 10.1007/s12010-022-04272-1. 36542270 10.1007/s12010-022-04272-1

[58] Gwak H, Park S, Kim J, Lee JD, Kim I-S, Kim S-I, *et al*. Microfluidic chip for rapid and selective isolation of tumor-derived extracellular vesicles for early diagnosis and metastatic risk evaluation of breast cancer. Biosens Bioelectron. 2021;192:113495. 10.1016/j.bios.2021.113495. 34273737 10.1016/j.bios.2021.113495

[59] Chen W, Li H, Su W, Qin J. Microfluidic device for on-chip isolation and detection of circulating exosomes in blood of breast cancer patients. Biomicrofluidics. 2019;13:054113. 10.1063/1.5110973. 31893011 10.1063/1.5110973 PMC6932858

[60] Chen X, Liu X, Zhang C, Xia H, Qin X, Guo Y, *et al*. Isolation and detection of exosomes on microfluidic chips. Biomedical Instrumentation. 2025;1:100007. 10.1016/j.bmi.2025.100007.

[61] Singh K, Nalabotala R, M. Koo K, Bose S, Nayak R, A. Shiddiky MJ, *et al*. Separation of distinct exosome subpopulations: isolation and characterization approaches and their associated challenges. Analyst. 2021;146:3731–49. 10.1039/D1AN00024A. 33988193 10.1039/d1an00024a

[62] Kim G, Seo M, Xu J, Park J, Gim S, Chun H. Large-area silicon nitride nanosieve for enhanced diffusion-based exosome isolation. Small Methods. 2024;8:e2301624. 10.1002/smtd.202301624. 38801014 10.1002/smtd.202301624

[63] Kim G, Kim K-H, Seo M, Kim T, Chun H. High-throughput exosome isolation based on bidirectional flow control through nanoporous membrane. Sens Actuators B Chem. 2024;418:136233. 10.1016/j.snb.2024.136233.

[64] Kim G, Park MC, Jang S, Han D, Kim H, Kim W, *et al*. Diffusion-Based Separation of Extracellular Vesicles by Nanoporous Membrane Chip. Biosensors (Basel). 2021;11:347. 10.3390/bios11090347. 34562937 10.3390/bios11090347 PMC8472239

[65] Han SB, Lee SS, Multidisciplinary Digital Publishing Institute. Simultaneous detection of exosomal microRNAs isolated from cancer cells using surface acoustic wave sensor array with high sensitivity and reproducibility. Micromachines. 2024;15:249. 10.3390/mi15020249. 38398977 10.3390/mi15020249 PMC10892992

[66] Nikoloff JM, Saucedo-Espinosa MA, Dittrich PS, American Chemical Society. Microfluidic platform for profiling of extracellular vesicles from single breast cancer cells. Anal Chem. 2023;95:1933–9. 10.1021/acs.analchem.2c04106. 36608325 10.1021/acs.analchem.2c04106 PMC9878503

[67] Liu J, Qu Y, Wang H, Multidisciplinary Digital Publishing Institute. Immuno-acoustic sorting of disease-specific extracellular vesicles by acoustophoretic force. Micromachines. 2021;12:1534. 10.3390/mi12121534. 34945384 10.3390/mi12121534 PMC8709371

[68] Kurian TK, Banik S, Gopal D, Chakrabarti S, Mazumder N. Elucidating methods for isolation and quantification of exosomes: a review. Mol Biotechnol. 2021;63:249–66. 10.1007/s12033-021-00300-3. 33492613 10.1007/s12033-021-00300-3 PMC7940341

[69] Lan M, Yang F. Applications of dielectrophoresis in microfluidic-based exosome separation and detection. Chem Eng J. 2024;491:152067. 10.1016/j.cej.2024.152067.

[70] Kim S, Song J, Roh SM, Kim HJ, Kim H, Lee S, *et al*. Efficient exosome separation utilizing dielectrophoretic force in conductive spiral microfluidic chips and validation via a reduced graphene oxide (rGO)-based biosensor. Sens Actuators, B Chem. 2024;404:135207. 10.1016/j.snb.2023.135207.

[71] Lan M, Ren Z, Cheng C, Li G, Yang F. Small extracellular vesicles detection using dielectrophoresis-based microfluidic chip for diagnosis of breast cancer. Biosens Bioelectron. 2024;259:116382. 10.1016/j.bios.2024.116382. 38749284 10.1016/j.bios.2024.116382

[72] van der Pol E, Böing AN, Harrison P, Sturk A, Nieuwland R. Classification, functions, and clinical relevance of extracellular vesicles. Pharmacol Rev. 2012;64:676–705. 10.1124/pr.112.005983. 22722893 10.1124/pr.112.005983

[73] I. Ramirez M, G. Amorim M, Gadelha C, Milic I, A. Welsh J, M. Freitas V, *et al*. Technical challenges of working with extracellular vesicles. Nanoscale. Royal Society of Chemistry; 2018;10:881–906. 10.1039/C7NR08360B. 10.1039/c7nr08360b 29265147

[74] Gardiner C, Di Vizio D, Sahoo S, Théry C, Witwer KW, Wauben M, *et al*. Techniques used for the isolation and characterization of extracellular vesicles: results of a worldwide survey. J Extracell Vesicles. 2016;5:32945. 10.3402/jev.v5.32945. 27802845 10.3402/jev.v5.32945 PMC5090131

[75] Andreu Z, Rivas E, Sanguino-Pascual A, Lamana A, Marazuela M, González-Alvaro I, *et al*. Comparative analysis of EV isolation procedures for miRNAs detection in serum samples. J Extracell Vesicles. 2016;5:31655. 10.3402/jev.v5.31655. 27330048 10.3402/jev.v5.31655 PMC4916259

[76] Davies RT, Kim J, Jang SC, Choi E-J, Gho YS, Park J, *et al*. Microfluidic filtration system to isolate extracellular vesicles from blood. Lab Chip. 2012;12:5202–10. 10.1039/C2LC41006K. 23111789 10.1039/c2lc41006k

[77] Nordin JZ, Lee Y, Vader P, Mäger I, Johansson HJ, Heusermann W, *et a*l. Ultrafiltration with size-exclusion liquid chromatography for high yield isolation of extracellular vesicles preserving intact biophysical and functional properties. Nanomedicine. 2015;11:879–83. 10.1016/j.nano.2015.01.003. 25659648 10.1016/j.nano.2015.01.003

[78] Wahlund CJE, Eklund A, Grunewald J, Gabrielsson S. Pulmonary extracellular vesicles as mediators of local and systemic inflammation. Front Cell Dev Biol. 2017;5:39. 10.3389/fcell.2017.00039. 28491866 10.3389/fcell.2017.00039 PMC5405144

[79] Kalra H, Adda CG, Liem M, Ang C-S, Mechler A, Simpson RJ, *et al*. Comparative proteomics evaluation of plasma exosome isolation techniques and assessment of the stability of exosomes in normal human blood plasma. Proteomics. 2013;13:3354–64. 10.1002/pmic.201300282. 24115447 10.1002/pmic.201300282

[80] Raposo G, Stoorvogel W. Extracellular vesicles: exosomes, microvesicles, and friends. J Cell Biol. 2013;200:373–83. 10.1083/jcb.201211138. 23420871 10.1083/jcb.201211138 PMC3575529

[81] Tauro BJ, Greening DW, Mathias RA, Ji H, Mathivanan S, Scott AM, *et al*. Comparison of ultracentrifugation, density gradient separation, and immunoaffinity capture methods for isolating human colon cancer cell line LIM1863-derived exosomes. Methods. 2012;56:293–304. 10.1016/j.ymeth.2012.01.002. 22285593 10.1016/j.ymeth.2012.01.002

[82] Popovic M, Mazzega E, Toffoletto B, de Marco A. Isolation of anti-extra-cellular vesicle single-domain antibodies by direct panning on vesicle-enriched fractions. Microb Cell Fact. 2018;17:6. 10.1186/s12934-017-0856-9. 29331148 10.1186/s12934-017-0856-9 PMC5766977

[83] Bano R, Ahmad F, Mohsin M, Royal Society of Chemistry. A perspective on the isolation and characterization of extracellular vesicles from different biofluids. RSC Advances. 2021;11:19598–615. 10.1039/D1RA01576A. 35479207 10.1039/d1ra01576a PMC9033677

[84] Caby M-P, Lankar D, Vincendeau-Scherrer C, Raposo G, Bonnerot C. Exosomal-like vesicles are present in human blood plasma. Int Immunol. 2005;17:879–87. 10.1093/intimm/dxh267. 15908444 10.1093/intimm/dxh267

[85] Balaj L, Atai NA, Chen W, Mu D, Tannous BA, Breakefield XO, *et al*. Heparin affinity purification of extracellular vesicles. Sci Rep. 2015;5:10266. 10.1038/srep10266. 25988257 10.1038/srep10266 PMC4437317

[86] Yoo CE, Kim G, Kim M, Park D, Kang HJ, Lee M, *et al*. A direct extraction method for microRNAs from exosomes captured by immunoaffinity beads. Anal Biochem. 2012;431:96–8. 10.1016/j.ab.2012.09.008. 22982508 10.1016/j.ab.2012.09.008

[87] Conde-Vancells J, Rodriguez-Suarez E, Embade N, Gil D, Matthiesen R, Valle M, *et al*. Characterization and comprehensive proteome profiling of exosomes secreted by hepatocytes. J Proteome Res. 2008;7:5157–66. 10.1021/pr8004887. 19367702 10.1021/pr8004887 PMC2696236

[88] Jakobsen KR, Paulsen BS, Bæk R, Varming K, Sorensen BS, Jørgensen MM. Exosomal proteins as potential diagnostic markers in advanced non-small cell lung carcinoma. J Extracell Vesicles. 2015;4:26659. 10.3402/jev.v4.26659. 25735706 10.3402/jev.v4.26659 PMC4348413

[89] Wang Z, Wu H, Fine D, Schmulen J, Hu Y, Godin B, *et al*. Ciliated micropillars for the microfluidic-based isolation of nanoscale lipid vesicles. Lab Chip. 2013;13:2879–82. 10.1039/C3LC41343H. 10.1039/c3lc41343h PMC3740541 23743667

[90] Böing AN, van der Pol E, Grootemaat AE, Coumans FAW, Sturk A, Nieuwland R. Single-step isolation of extracellular vesicles by size-exclusion chromatography. J Extracell Vesicles. 2014;3. 10.3402/jev.v3.23430. 10.3402/jev.v3.23430 PMC4159761 25279113

[91] Sódar BW, Kittel Á, Pálóczi K, Vukman KV, Osteikoetxea X, Szabó-Taylor K, *et al*. Low-density lipoprotein mimics blood plasma-derived exosomes and microvesicles during isolation and detection. Sci Rep. 2016;6:24316. 10.1038/srep24316. 27087061 10.1038/srep24316 PMC4834552

[92] Lobb RJ, Becker M, Wen SW, Wong CSF, Wiegmans AP, Leimgruber A, *et al*. Optimized exosome isolation protocol for cell culture supernatant and human plasma. J Extracell Vesicles. 2015;4:27031. 10.3402/jev.v4.27031. 26194179 10.3402/jev.v4.27031 PMC4507751

[93] Gámez-Valero A, Monguió-Tortajada M, Carreras-Planella L, Franquesa M, Beyer K, Borràs FE. Size-exclusion chromatography-based isolation minimally alters extracellular vesicles’ characteristics compared to precipitating agents. Sci Rep. 2016;6:33641. 10.1038/srep33641. 27640641 10.1038/srep33641 PMC5027519

[94] Vergauwen G, Dhondt B, Van Deun J, De Smedt E, Berx G, Timmerman E, *et al*. Confounding factors of ultrafiltration and protein analysis in extracellular vesicle research. Sci Rep. 2017;7:2704. 10.1038/s41598-017-02599-y. 28577337 10.1038/s41598-017-02599-y PMC5457435

[95] Boriachek K, Islam M, Möller A, Salomon C, Nguyen N-T, Hossain M, *et al.* Biological functions and current advances in isolation and detection strategies for exosome nanovesicles. Small. 2018;14:1702153. 10.1002/smll.201702153. 10.1002/smll.201702153 29282861

[96] Engvall E, Perlmann P. Enzyme-linked immunosorbent assay (ELISA) quantitative assay of immunoglobulin G. Immunochemistry. 1971;8:871–4. 10.1016/0019-2791(71)90454-X. 5135623 10.1016/0019-2791(71)90454-x

[97] Zarovni N, Corrado A, Guazzi P, Zocco D, Lari E, Radano G, *et al*. Integrated isolation and quantitative analysis of exosome shuttled proteins and nucleic acids using immunocapture approaches. Methods. 2015;87:46–58. 10.1016/j.ymeth.2015.05.028. 26044649 10.1016/j.ymeth.2015.05.028

[98] Logozzi M, Milito AD, Lugini L, Borghi M, Calabrò L, Spada M, *et al*. High levels of exosomes expressing CD63 and Caveolin-1 in plasma of melanoma patients. PLoS One. 2009;4:e5219. 10.1371/journal.pone.0005219. 10.1371/journal.pone.0005219 PMC2667632 19381331

[99] Lässer C, Eldh M, Lötvall J. Isolation and Characterization of RNA-Containing Exosomes. Journal of Visualized Experiments (JoVE). 2012;e3037. 10.3791/3037. 10.3791/3037 PMC3369768 22257828

[100] Hannafon BN, Trigoso YD, Calloway CL, Zhao YD, Lum DH, Welm AL, *et al*. Plasma exosome microRNAs are indicative of breast cancer. Breast Cancer Res. 2016;18:90. 10.1186/s13058-016-0753-x. 27608715 10.1186/s13058-016-0753-x PMC5016889

[101] Erdbrügger U, Lannigan J. Analytical challenges of extracellular vesicle detection: a comparison of different techniques. Cytometry A. 2016;89:123–34. 10.1002/cyto.a.22795. 26651033 10.1002/cyto.a.22795

[102] Kim HK, Song KS, Lee ES, Lee YJ, Park YS, Lee KR, *et al*. Optimized flow cytometric assay for the measurement of platelet microparticles in plasma: pre-analytic and analytic considerations. Blood Coagul Fibrinolysis. 2002;13:393–7. 10.1097/00001721-200207000-00003. 12138366 10.1097/00001721-200207000-00003

[103] Kesimer M, Scull M, Brighton B, DeMaria G, Burns K, O’Neal W, *et al*. Characterization of exosome-like vesicles released from human tracheobronchial ciliated epithelium: a possible role in innate defense. FASEB J. 2009;23:1858–68. 10.1096/fj.08-119131. 19190083 10.1096/fj.08-119131 PMC2698655

[104] Julius MH, Masuda T, Herzenberg LA. Demonstration that antigen-binding cells are precursors of antibody-producing cells after purification with a fluorescence-activated cell sorter. Proc Natl Acad Sci U S A. 1972;69:1934–8. 10.1073/pnas.69.7.1934. 4114858 10.1073/pnas.69.7.1934 PMC426835

[105] Hunter MP, Ismail N, Zhang X, Aguda BD, Lee EJ, Yu L, *et al*. Detection of microRNA Expression in Human Peripheral Blood Microvesicles. PLOS ONE. Public Library of Science; 2008;3:e3694. 10.1371/journal.pone.0003694. 10.1371/journal.pone.0003694 PMC2577891 19002258

[106] Yu D, Li Y, Wang M, Gu J, Xu W, Cai H, *et al*. Exosomes as a new frontier of cancer liquid biopsy. Mol Cancer. 2022;21:56. 10.1186/s12943-022-01509-9. 35180868 10.1186/s12943-022-01509-9 PMC8855550

[107] Moura SL, Martín CG, Martí M, Pividori MI. Multiplex detection and characterization of breast cancer exosomes by magneto-actuated immunoassay. Talanta. 2020;211:120657. 10.1016/j.talanta.2019.120657. 32070615 10.1016/j.talanta.2019.120657

[108] Zhu S, Ma L, Wang S, Chen C, Zhang W, Yang L, *et al*. Light-scattering detection below the level of single fluorescent molecules for high-resolution characterization of functional nanoparticles. ACS Nano. 2014;8:10998–1006. 10.1021/nn505162u. 25300001 10.1021/nn505162u PMC4212780

[109] Carr B, Hole P, Malloy A, Nelson P, Wright M, Smith J. Applications of nanoparticle tracking analysis in nanoparticle research - a mini review. Eur J Parenter Pharm Sci. 2009;14:45–50.

[110] Risha Y, Minic Z, Ghobadloo SM, Berezovski MV. The proteomic analysis of breast cell line exosomes reveals disease patterns and potential biomarkers. Sci Rep. 2020;10:13572. 10.1038/s41598-020-70393-4. 32782317 10.1038/s41598-020-70393-4 PMC7419295

[111] Maas SLN, Broekman MLD, de Vrij J. Tunable resistive pulse sensing for the characterization of extracellular vesicles. Methods Mol Biol. 2017;1545:21–33. 10.1007/978-1-4939-6728-5_2. 27943204 10.1007/978-1-4939-6728-5_2

[112] Vogel R, Willmott G, Kozak D, Roberts GS, Anderson W, Groenewegen L, *et al*. Quantitative sizing of nano/microparticles with a tunable elastomeric pore sensor. Anal Chem. 2011;83:3499–506. 10.1021/ac200195n. 21434639 10.1021/ac200195n

[113] Vogel R, Coumans FAW, Maltesen RG, Böing AN, Bonnington KE, Broekman ML, *et al*. A standardized method to determine the concentration of extracellular vesicles using tunable resistive pulse sensing. J Extracell Vesicles. 2016;5:31242. 10.3402/jev.v5.31242. 27680301 10.3402/jev.v5.31242 PMC5040823

[114] Jia R, Rotenberg SA, Mirkin MV. Electrochemical resistive-pulse sensing of extracellular vesicles. Anal Chem. 2022;94:12614–20. 10.1021/acs.analchem.2c01216. 36083276 10.1021/acs.analchem.2c01216

[115] Coumans FAW, van der Pol E, Böing AN, Hajji N, Sturk G, van Leeuwen TG, *et al*. Reproducible extracellular vesicle size and concentration determination with tunable resistive pulse sensing. Journal of Extracellular Vesicles. Taylor & Francis; 2014;3:25922. 10.3402/jev.v3.25922. 10.3402/jev.v3.25922 PMC4263901 25498889

[116] Odenthal KJ, Gooding JJ. An introduction to electrochemical DNAbiosensors. Analyst. 2007;132:603–10. 10.1039/B701816A. 17592577 10.1039/b701816a

[117] Jeong S, Park J, Pathania D, Castro CM, Weissleder R, Lee H. Integrated magneto-electrochemical sensor for exosome analysis. ACS Nano. 2016;10:1802–9. 10.1021/acsnano.5b07584. 26808216 10.1021/acsnano.5b07584 PMC4802494

[118] Doldán X, Fagúndez P, Cayota A, Laíz J, Tosar JP. Electrochemical sandwich immunosensor for determination of exosomes based on surface marker-mediated signal amplification. Anal Chem. 2016;88:10466–73. 10.1021/acs.analchem.6b02421. 27734678 10.1021/acs.analchem.6b02421

[119] Makler A, Asghar W. Exosomal biomarkers for cancer diagnosis and patient monitoring. Expert Rev Mol Diagn. 2020;20:387–400. 10.1080/14737159.2020.1731308. 32067543 10.1080/14737159.2020.1731308 PMC7071954

[120] Sabapatha A, Gercel-Taylor C, Taylor DD. Specific isolation of placenta-derived exosomes from the circulation of pregnant women and their immunoregulatory consequences. Am J Reprod Immunol. 2006;56:345–55. 10.1111/j.1600-0897.2006.00435.x. 17076679 10.1111/j.1600-0897.2006.00435.x

[121] Merchant ML, Powell DW, Wilkey DW, Cummins TD, Deegens JK, Rood IM, *et al*. Microfiltration isolation of human urinary exosomes for characterization by MS. Proteomics Clin Appl. 2010;4:84–96. 10.1002/prca.200800093. 21137018 10.1002/prca.200800093

[122] Lötvall J, Hill AF, Hochberg F, Buzás EI, Di Vizio D, Gardiner C, *et al*. Minimal experimental requirements for definition of extracellular vesicles and their functions: a position statement from the International Society for Extracellular Vesicles. J Extracell Vesicles. 2014;3:26913. 10.3402/jev.v3.26913. 25536934 10.3402/jev.v3.26913 PMC4275645

[123] Clayton A, Court J, Navabi H, Adams M, Mason MD, Hobot JA, *et al*. Analysis of antigen presenting cell derived exosomes, based on immuno-magnetic isolation and flow cytometry. J Immunol Methods. 2001;247:163–74. 10.1016/S0022-1759(00)00321-5. 11150547 10.1016/s0022-1759(00)00321-5

[124] Ma H, Li J, Gao M, Dong Y, Luo Y, Su S. An electrochemical aptasensor for accurate and sensitive detection of exosomes based on dual-probe recognition and hybridization chain reaction. Biosensors (Basel). 2025;15:302. 10.3390/bios15050302. 40422041 10.3390/bios15050302 PMC12109912

[125] Liu C, Yang Y, Wu Y. Recent advances in exosomal protein detection via liquid biopsy biosensors for cancer screening, diagnosis, and prognosis. AAPS J. 2018;20:41. 10.1208/s12248-018-0201-1. 29520676 10.1208/s12248-018-0201-1 PMC8628518

[126] Serrano-Pertierra E, Oliveira-Rodríguez M, Rivas M, Oliva P, Villafani J, Navarro A, *et al*. Characterization of Plasma-Derived Extracellular Vesicles Isolated by Different Methods: A Comparison Study. Bioengineering [Internet]. publisher; 2019 [cited 2025 Dec 26];6. 10.3390/bioengineering6010008. 10.3390/bioengineering6010008 PMC6466225 30658418

[127] Lyu TS, Ahn Y, Im Y-J, Kim S-S, Lee K-H, Kim J, *et al*. The characterization of exosomes from fibrosarcoma cell and the useful usage of Dynamic Light Scattering (DLS) for their evaluation. PLoS One. 2021;16:e0231994. 10.1371/journal.pone.0231994. 33497388 10.1371/journal.pone.0231994 PMC7837462

[128] Hoo CM, Starostin N, West P, Mecartney ML. A comparison of atomic force microscopy (AFM) and dynamic light scattering (DLS) methods to characterize nanoparticle size distributions. J Nanopart Res. 2008;10:89–96. 10.1007/s11051-008-9435-7.

[129] Bryant G, Thomas JC. Improved particle size distribution measurements using multiangle dynamic light scattering. Langmuir. 1995;11:2480–5. 10.1021/la00007a028.

[130] Chen B, Qiu X. Surface-enhanced raman scattering (SERS) for exosome detection. Clin Chim Acta. 2025;568:120148. 10.1016/j.cca.2025.120148. 39842651 10.1016/j.cca.2025.120148

[131] Zhang P, Wang L, Fang Y, Zheng D, Lin T, Wang H. Label-free exosomal detection and classification in rapid discriminating different cancer types based on specific raman phenotypes and multivariate statistical analysis. Molecules. 2019;24:2947. 10.3390/molecules24162947. 31416240 10.3390/molecules24162947 PMC6720265

[132] Xie Y, Su X, Wen Y, Zheng C, Li M, American Chemical Society. Artificial intelligent label-free SERS profiling of serum exosomes for breast cancer diagnosis and postoperative assessment. Nano Lett. 2022;22:7910–8. 10.1021/acs.nanolett.2c02928. 36149810 10.1021/acs.nanolett.2c02928

[133] Im H, Shao H, Park YI, Peterson VM, Castro CM, Weissleder R, *et al*. Label-free detection and molecular profiling of exosomes with a nano-plasmonic sensor. Nat Biotechnol. 2014;32:490–5. 10.1038/nbt.2886. 24752081 10.1038/nbt.2886 PMC4356947

[134] Sina AAI, Vaidyanathan R, Wuethrich A, Carrascosa LG, Trau M. Label-free detection of exosomes using a surface plasmon resonance biosensor. Anal Bioanal Chem. 2019;411:1311–8. 10.1007/s00216-019-01608-5. 30719562 10.1007/s00216-019-01608-5

[135] De Sousa KP, Rossi I, Abdullahi M, Ramirez MI, Stratton D, Inal JM. Isolation and characterization of extracellular vesicles and future directions in diagnosis and therapy. Wiley Interdiscip Rev Nanomed Nanobiotechnol. 2023;15:e1835. 10.1002/wnan.1835. 35898167 10.1002/wnan.1835 PMC10078256

[136] Kalimuthu S, Gangadaran P, Rajendran RL, Zhu L, Oh JM, Lee HW, *et al*. A new approach for loading anticancer drugs into mesenchymal stem cell-derived exosome mimetics for cancer therapy. Front Pharmacol. 2018. 10.3389/fphar.2018.01116. 10.3389/fphar.2018.01116 PMC6168623 30319428

[137] Farhadi S, Mohammadi-Yeganeh S, Kiani J, Hashemi SM, Koochaki A, Sharifi K, *et al*. Exosomal delivery of 7SK long non-coding RNA suppresses viability, proliferation, aggressiveness and tumorigenicity in triple negative breast cancer cells. Life Sci. 2023;322:121646. 10.1016/j.lfs.2023.121646. 37011870 10.1016/j.lfs.2023.121646

[138] Ebrahimian M, Hashemi M, Etemad L, Salmasi Z. Thymoquinone-loaded mesenchymal stem cell-derived exosome as an efficient nano-system against breast cancer cells. Iran J Basic Med Sci. 2022;25:723–31. 10.22038/IJBMS.2022.64092.14116. 35949303 10.22038/IJBMS.2022.64092.14116 PMC9320205

[139] Donoso-Quezada J, Guajardo-Flores D, González-Valdez J. Enhanced exosome-mediated delivery of black bean phytochemicals (*Phaseolus vulgaris* L.) for cancer treatment applications. Biomed Pharmacother. 2020;131:110771. 10.1016/j.biopha.2020.110771. 33152932 10.1016/j.biopha.2020.110771

[140] Jang J-Y, Lee J-K, Jeon Y-K, Kim C-W. Exosome derived from epigallocatechin gallate treated breast cancer cells suppresses tumor growth by inhibiting tumor-associated macrophage infiltration and M2 polarization. BMC Cancer [Internet]. Springer Science and Business Media LLC; 2013;13. 10.1186/1471-2407-13-421. 10.1186/1471-2407-13-421 PMC3848851 24044575

[141] Zhao L, Gu C, Gan Y, Shao L, Chen H, Zhu H. Exosome-mediated siRNA delivery to suppress postoperative breast cancer metastasis. J Control Release. 2020;318:1–15. 10.1016/j.jconrel.2019.12.005. 31830541 10.1016/j.jconrel.2019.12.005

[142] Kim H, Rhee WJ. Exosome-mediated Let7c-5p delivery for breast cancer therapeutic development. Biotechnol Bioprocess Eng. 2020;25:513–20. 10.1007/s12257-020-0002-0.

[143] Hashemi ZS, Ghavami M, Kiaie SH, Mohammadi F, Barough MS, Khalili S, *et al*. Novel delivery of sorafenib by natural killer cell-derived exosomes-enhanced apoptosis in triple-negative breast cancer. Nanomedicine (Lond). 2023;18:437–53. 10.2217/nnm-2022-0237. 37199259 10.2217/nnm-2022-0237

[144] Yu Y, Li T, Ou M, Luo R, Chen H, Ren H, *et al*. OX40L-expressing M1-like macrophage exosomes for cancer immunotherapy. J Control Release. 2024;365:469–79. 10.1016/j.jconrel.2023.11.051. 38040340 10.1016/j.jconrel.2023.11.051

[145] Huang L, Rong Y, Tang X, Yi K, Qi P, Hou J, *et al*. Engineered exosomes as an in situ DC-primed vaccine to boost antitumor immunity in breast cancer. Mol Cancer. 2022;21:45. 10.1186/s12943-022-01515-x. 35148751 10.1186/s12943-022-01515-x PMC8831689

[146] Llevenes P, Chen A, Lawton M, Rondón-Ortiz AN, Qiu Y, Seen M, *et al*. Plasma Exosomes in Insulin Resistant Obesity Exacerbate Progression of Triple Negative Breast Cancer. bioRxiv. 2025;2024.10.10.617639. 10.1101/2024.10.10.617639. 10.1186/s12885-025-14447-8 PMC12239313 40629272

[147] Shadi Vaziri S, Tajbakhsh E, Khamesipour F, Momtaz H, Mazaheri Z. Impact of *Helicobacter pylori*-derived outer membrane vesicles on inflammation, immune responses, and tumor cell migration in breast cancer through the snail/Β-catenin pathway. Rep Biochem Mol Biol. 2024;13:263–72. 10.61186/rbmb.13.2.263. 39995644 10.61186/rbmb.13.2.263 PMC11847590

[148] Li Y, Wu J, Qiu X, Dong S, He J, Liu J, *et al*. Bacterial outer membrane vesicles-based therapeutic platform eradicates triple-negative breast tumor by combinational photodynamic/chemo-/immunotherapy. Bioact Mater. 2023;20:548–60. 10.1016/j.bioactmat.2022.05.037. 35846843 10.1016/j.bioactmat.2022.05.037 PMC9253654

[149] An J, Kwon H, Lim W, Moon B-I. *Staphylococcus aureus*-derived extracellular vesicles enhance the efficacy of endocrine therapy in breast cancer cells. J Clin Med. 2022;11:2030. 10.3390/jcm11072030. 35407638 10.3390/jcm11072030 PMC9000115

[150] González-Sarrías A, Iglesias-Aguirre CE, Cortés-Martín A, Vallejo F, Cattivelli A, del Pozo-Acebo L, *et al*. Milk-Derived Exosomes as Nanocarriers to Deliver Curcumin and Resveratrol in Breast Tissue and Enhance Their Anticancer Activity. International Journal of Molecular Sciences. MDPI AG; 2022;23:2860. 10.3390/ijms23052860. 10.3390/ijms23052860 PMC8911159 35270004

[151] Gupta P, Marwaha D, Sharma S, Sethi VA. Hypothesizing the co-delivery of raloxifene and genistein by bovine milk exosomes for enhanced drug delivery in breast cancer management. Med Hypotheses. 2025;199:111644. 10.1016/j.mehy.2025.111644.

[152] Anusha R, Ashin M, Priya S. Ginger exosome-like nanoparticles (GELNs) induced apoptosis, cell cycle arrest, and anti-metastatic effects in triple-negative breast cancer MDA-MB-231 cells. Food Chem Toxicol. 2023;182:114102. 10.1016/j.fct.2023.114102. 37865333 10.1016/j.fct.2023.114102

[153] Salem D, Abdel-Ghany S, Mohamed E, Alahmady N, Alqosaibi A, Al-Dhuayan I, *et al*. Natural nanoparticles for drug delivery: proteomic insights and anticancer potential of doxorubicin-loaded avocado exosomes. Pharmaceuticals. 2025;18:844. 10.3390/ph18060844. 10.3390/ph18060844 PMC12195682 40573239

[154] Chen Q, Li Q, Liang Y, Zu M, Chen N, Canup BSB,* et al*. Natural exosome-like nanovesicles from edible tea flowers suppress metastatic breast cancer via ROS generation and microbiota modulation. Acta Pharmaceutica Sinica B Elsevier BV. 2022;12:907–23. 10.1016/j.apsb.2021.08.016. 10.1016/j.apsb.2021.08.016 PMC8897038 35256954

[155] Chen Q, Zu M, Gong H, Ma Y, Sun J, Ran S, *et al*. Tea leaf-derived exosome-like nanotherapeutics retard breast tumor growth by pro-apoptosis and microbiota modulation. J Nanobiotechnology. 2023. 10.1186/s12951-022-01755-5. 10.1186/s12951-022-01755-5 PMC9811040 36600299

[156] Han X, Wei Q, Lv Y, Weng L, Huang H, Wei Q, *et al*. Ginseng-derived nanoparticles potentiate immune checkpoint antibody efficacy by reprogramming the cold tumor microenvironment. Mol Ther. 2022;30:327–40. 10.1016/j.ymthe.2021.08.028. 34450250 10.1016/j.ymthe.2021.08.028 PMC8753455

[157] Cui L, Perini G, Augello A, Palmieri V, De Spirito M, Papi M. Plant-derived extracellular nanovesicles: a promising biomedical approach for effective targeting of triple negative breast cancer cells. Front Bioeng Biotechnol. 2024. 10.3389/fbioe.2024.1390708. 38952670 10.3389/fbioe.2024.1390708 PMC11215178

[158] Colombo M, Raposo G, Théry C, Annual Reviews. Biogenesis, secretion, and intercellular interactions of exosomes and other extracellular vesicles. Annu Rev Cell Dev Biol. 2014;30:255–89. 10.1146/annurev-cellbio-101512-122326. 25288114 10.1146/annurev-cellbio-101512-122326

[159] Al-Awsi GRL, Alsaikhan F, Margiana R, Ahmad I, Patra I, Najm MAA, *et al*. Shining the light on mesenchymal stem cell-derived exosomes in breast cancer. Stem Cell Res Ther. 2023;14:21. 10.1186/s13287-023-03245-3. 36750912 10.1186/s13287-023-03245-3 PMC9906907

[160] Zhang F, Guo J, Zhang Z, Qian Y, Wang G, Duan M, *et al*. Mesenchymal stem cell-derived exosome: A tumor regulator and carrier for targeted tumor therapy. Cancer Lett. 2022;526:29–40. 10.1016/j.canlet.2021.11.015. 34800567 10.1016/j.canlet.2021.11.015

[161] Zhang L. The role of mesenchymal stem cells in modulating the breast cancer microenvironment. Cell Transplant. 2023;32:09636897231220073. 10.1177/09636897231220073. 38135917 10.1177/09636897231220073 PMC10748553

[162] Wiest EF, Zubair AC, Multidisciplinary Digital Publishing Institute. Generation of Current Good Manufacturing Practices-Grade Mesenchymal Stromal Cell-Derived Extracellular Vesicles Using Automated Bioreactors. Biology (Basel). 2025;14:313. 10.3390/biology14030313. 40136569 10.3390/biology14030313 PMC11940689

[163] Zhu L, Kalimuthu S, Gangadaran P, Oh JM, Lee HW, Baek SH, *et al*. Exosomes derived from natural killer cells exert therapeutic effect in melanoma. Theranostics. 2017;7:2732–45. 10.7150/thno.18752. 28819459 10.7150/thno.18752 PMC5558565

[164] Luo H, Zhou Y, Zhang J, Zhang Y, Long S, Lin X, *et al*. Nk cell-derived exosomes enhance the anti-tumor effects against ovarian cancer by delivering cisplatin and reactivating NK cell functions. Front Immunol [Internet]. 2023. 10.3389/fimmu.2022.1087689. 10.3389/fimmu.2022.1087689 PMC9892755 36741396

[165] Zhang A, Yin X, Ma J, Springer Science and Business Media LLC. NK-derived exosomes in anti-tumor strategies. Medical Oncology [Internet]. 2025. 10.1007/s12032-025-02965-1. 40782186 10.1007/s12032-025-02965-1 PMC12335398

[166] Robbins PD, Morelli AE. Regulation of immune responses by extracellular vesicles. Nat Rev Immunol. 2014;14:195–208. 10.1038/nri3622. 24566916 10.1038/nri3622 PMC4350779

[167] Zitvogel L, Regnault A, Lozier A, Wolfers J, Flament C, Tenza D, *et al*. Eradication of established murine tumors using a novel cell-free vaccine: dendritic cell-derived exosomes. Nat Med. 1998;4:594–600. 10.1038/nm0598-594. 9585234 10.1038/nm0598-594

[168] Zhou W, Yang F, Zhang X. Roles of M1 macrophages and their extracellular vesicles in cancer therapy. Cells. 2024;13:1428. 10.3390/cells13171428. 39273000 10.3390/cells13171428 PMC11394047

[169] Liu L, Zhang S, Ren Y, Wang R, Zhang Y, Weng S, *et al*. Macrophage-derived exosomes in cancer: a double-edged sword with therapeutic potential. J Nanobiotechnol. 2025;23:319. 10.1186/s12951-025-03321-1. 10.1186/s12951-025-03321-1 PMC12034189 40287762

[170] Shao H, Im H, Castro CM, Breakefield X, Weissleder R, Lee H. New technologies for analysis of extracellular vesicles. Chem Rev. 2018;118:1917–50. 10.1021/acs.chemrev.7b00534. 29384376 10.1021/acs.chemrev.7b00534 PMC6029891

[171] Bai S, Wei Y, Liu R, Xu R, Xiang L, Du J. Role of tumour-derived exosomes in metastasis. Biomed Pharmacother. 2022;147:112657. 10.1016/j.biopha.2022.112657. 35078096 10.1016/j.biopha.2022.112657

[172] Wu X, Meng Y, Yao Z, Lin X, Hu M, Cai S, *et al*. Extracellular vesicles as nature’s nano carriers in cancer therapy: insights toward preclinical studies and clinical applications. Pharmacol Res. 2025;217:107751. 10.1016/j.phrs.2025.107751. 40345354 10.1016/j.phrs.2025.107751

[173] Ni C, Fang Q-Q, Chen W-Z, Jiang J-X, Jiang Z, Ye J, *et al*. Breast cancer-derived exosomes transmit lncRNA SNHG16 to induce CD73+γδ1 Treg cells. Sig Transduct Target Ther. 2020;5:41. 10.1038/s41392-020-0129-7. 10.1038/s41392-020-0129-7 PMC7188864 32345959

[174] Ding J, Ding X, Liao W, Lu Z. Red blood cell-derived materials for cancer therapy: construction, distribution, and applications. Mater Today Bio. 2024;24:100913. 10.1016/j.mtbio.2023.100913. 38188647 10.1016/j.mtbio.2023.100913 PMC10767221

[175] Ma S-R, Xia H-F, Gong P, Yu Z-L. Red Blood Cell-Derived Extracellular Vesicles: an overview of current research progress, challenges, and opportunities. Biomedicines. 2023;11:2798. 10.3390/biomedicines11102798. 37893171 10.3390/biomedicines11102798 PMC10604118

[176] Romano M, Musicò A, Zendrini A, Gilberti E, Orlandi F, Pedrazzi T, *et al*. Red Blood Cell-derived Extracellular Vesicles enable Cisplatin and Cetuximab Synergistic Therapy against Triple-Negative Breast Cancer [Internet]. bioRxiv; 2025 [cited 2025 Oct 8]. p. 2025.03.20.644320. 10.1101/2025.03.20.644320 10.1186/s12951-026-04628-3 PMC13471389 42252443

[177] Chai Q-Q, Li D, Zhang M, Gu Y-W, Li A-X, Wu X, *et al*. Engineering nanoplatforms of bacterial outer membrane vesicles to overcome cancer therapy resistance. Drug Resist Updates. 2025;83:101277. 10.1016/j.drup.2025.101277. 10.1016/j.drup.2025.101277 40712413

[178] Yang Y, Wu Y. Potential of bacterial outer membrane vesicles in tumor vaccine: characteristics, advancements, and future directions. Essays Biochem. 2025;69:EBC20253004. 10.1042/EBC20253004. 40159726 10.1042/EBC20253004 PMC12204010

[179] Cao H. Bacterial endotoxin lipopolysaccharides regulate gene expression in human colon cancer cells. BMC Res Notes. 2023;16:216. 10.1186/s13104-023-06506-9. 37705049 10.1186/s13104-023-06506-9 PMC10500902

[180] Sepahdar Z, Miroliaei M, Bouzari S, Khalaj V, Salimi M, Frontiers. Surface engineering of *Escherichia coli*–derived OMVs as promising nano-carriers to target EGFR-overexpressing breast cancer cells. Front Pharmacol [Internet]. 2021. 10.3389/fphar.2021.719289. 34867325 10.3389/fphar.2021.719289 PMC8638777

[181] Jiang Y, Wang L, Yang B, Ma G, Chen Z, Ma J, *et al*. *Bifidobacterium*-derived membrane vesicles inhibit triple-negative breast cancer growth by inducing tumor cell apoptosis. Mol Biol Rep. 2023;50:7547–56. 10.1007/s11033-023-08702-z. 37498438 10.1007/s11033-023-08702-z

[182] del Pozo‐Acebo L, López de las Hazas M, Margollés A, Dávalos A, García‐Ruiz A, Wiley. Eating microRNAs: pharmacological opportunities for cross‐kingdom regulation and implications in host gene and gut microbiota modulation. Br J Pharmacol. 2021;178:2218–45. 10.1111/bph.15421. 33644849 10.1111/bph.15421

[183] Adriano B, Cotto NM, Chauhan N, Jaggi M, Chauhan SC, Yallapu MM, *et al*. Milk exosomes: nature’s abundant nanoplatform for theranostic applications. Bioact Mater. 2021;6:2479–90. 10.1016/j.bioactmat.2021.01.009. 33553829 10.1016/j.bioactmat.2021.01.009 PMC7856328

[184] López de las Hazas M-C, del Pozo-Acebo L, Hansen MS, Gil-Zamorano J, Mantilla-Escalante DC, Gómez-Coronado D, *et al*. Dietary bovine milk miRNAs transported in extracellular vesicles are partially stable during GI digestion, are bioavailable and reach target tissues but need a minimum dose to impact on gene expression. Eur J Nutr. 2021;61:1043–56. 10.1007/s00394-021-02720-y. 10.1007/s00394-021-02720-y 34716465

[185] Timofeeva AM, Paramonik AP, Sedykh SS, Nevinsky GA, Multidisciplinary Digital Publishing Institute. Milk exosomes: next-generation agents for delivery of anticancer drugs and therapeutic nucleic acids. Int J Mol Sci. 2023;24:10194. 10.3390/ijms241210194. 37373342 10.3390/ijms241210194 PMC10298983

[186] Suharta S, Barlian A, Hidajah AC, Notobroto HB, Ana ID, Indariani S, *et al*. Plant‐derived exosome‐like nanoparticles: a concise review on its extraction methods, content, bioactivities, and potential as functional food ingredient. J Food Sci. 2021;86:2838–50. 10.1111/1750-3841.15787. 10.1111/1750-3841.15787 34151426

[187] Wang C, Tsai T, Lee C. Regulation of exosomes as biologic medicines: regulatory challenges faced in exosome development and manufacturing processes. Clin Transl Sci. 2024;17:e13904. 10.1111/cts.13904. 39115257 10.1111/cts.13904 PMC11307316

[188] Ha D, Yang N, Nadithe V. Exosomes as therapeutic drug carriers and delivery vehicles across biological membranes: current perspectives and future challenges. Acta Pharm Sin B. 2016;6:287–96. 10.1016/j.apsb.2016.02.001. 27471669 10.1016/j.apsb.2016.02.001 PMC4951582

[189] EL Andaloussi S, Mäger I, Breakefield XO, Wood MJA. Extracellular vesicles: biology and emerging therapeutic opportunities. Nat Rev Drug Discov. 2013;12:347–57. 10.1038/nrd3978. 23584393 10.1038/nrd3978

[190] Kumar DN, Chaudhuri A, Aqil F, Dehari D, Munagala R, Singh S, *et al*. Exosomes as emerging drug delivery and diagnostic modality for breast cancer: recent advances in isolation and application. Cancers (Basel). 2022;14:1435. 10.3390/cancers14061435. 35326585 10.3390/cancers14061435 PMC8946254

[191] Johnsen KB, Gudbergsson JM, Skov MN, Pilgaard L, Moos T, Duroux M. A comprehensive overview of exosomes as drug delivery vehicles - endogenous nanocarriers for targeted cancer therapy. Biochim Biophys Acta. 2014;1846:75–87. 10.1016/j.bbcan.2014.04.005. 24747178 10.1016/j.bbcan.2014.04.005

[192] Wang Y, Ma Q, Wang T, Xing J, Li Q, Wang D, *et al*. The involvement and application potential of exosomes in breast cancer immunotherapy. Front Immunol. 2024;15:1384946. 10.3389/fimmu.2024.1384946. 38835784 10.3389/fimmu.2024.1384946 PMC11148227

[193] Kamerkar S, LeBleu VS, Sugimoto H, Yang S, Ruivo CF, Melo SA, *et al*. Exosomes facilitate therapeutic targeting of oncogenic KRAS in pancreatic cancer. Nature. 2017;546:498–503. 10.1038/nature22341. 28607485 10.1038/nature22341 PMC5538883

[194] Guo L, Kong D, Liu J, Zhan L, Luo L, Zheng W, *et al*. Breast cancer heterogeneity and its implication in personalized precision therapy. Exp Hematol Oncol. 2023;12:3. 10.1186/s40164-022-00363-1. 36624542 10.1186/s40164-022-00363-1 PMC9830930

[195] Hashida M. Role of pharmacokinetic consideration for the development of drug delivery systems: a historical overview. Adv Drug Deliv Rev. 2020;157:71–82. 10.1016/j.addr.2020.06.015. 32565225 10.1016/j.addr.2020.06.015

[196] Chavda VP, Pandya A, Kumar L, Raval N, Vora LK, Pulakkat S, *et al*. Exosome nanovesicles: A potential carrier for therapeutic delivery. Nano Today. 2023;49:101771. 10.1016/j.nantod.2023.101771.

[197] Ferreira D, Moreira JN, Rodrigues LR. New advances in exosome-based targeted drug delivery systems. Crit Rev Oncol Hematol. 2022;172:103628. 10.1016/j.critrevonc.2022.103628. 35189326 10.1016/j.critrevonc.2022.103628

[198] Huang L, Wu E, Liao J, Wei Z, Wang J, Chen Z, *et al.* Research advances of engineered exosomes as drug delivery carrier. ACS Omega. 2023;8:43374–87. 10.1021/acsomega.3c04479. 38027310 10.1021/acsomega.3c04479 PMC10666244

[199] Roskoski JR. Targeted and cytotoxic inhibitors used in the treatment of breast cancer. Pharmacol Res. 2024;210:107534. 10.1016/j.phrs.2024.107534. 39631485 10.1016/j.phrs.2024.107534

[200] Kim MS, Haney MJ, Zhao Y, Mahajan V, Deygen I, Klyachko NL, *et al*. Development of exosome-encapsulated paclitaxel to overcome MDR in cancer cells. Nanomedicine Nanotechnology Biol Med. 2016;12:655–64. 10.1016/j.nano.2015.10.012. 10.1016/j.nano.2015.10.012 PMC4809755 26586551

[201] Liu M, Wang Y, Zhang Y, Hu D, Tang L, Zhou B, *et al*. Landscape of small nucleic acid therapeutics: moving from the bench to the clinic as next-generation medicines. Sig Transduct Target Ther. 2025;10:73. 10.1038/s41392-024-02112-8. 10.1038/s41392-024-02112-8 PMC11891339 40059188

[202] Maqsood Q, Khan MU, Fatima T, Khalid S, Malik ZI. Recent insights into breast cancer: molecular pathways, epigenetic regulation, and emerging targeted therapies. Breast Cancer (Auckl). 2025;19:11782234251355663. 10.1177/11782234251355663. 40661160 10.1177/11782234251355663 PMC12256763

[203] Gautam S, Maurya R, Vikal A, Patel P, Thakur S, Singh A, *et al*. Understanding drug resistance in breast cancer: Mechanisms and emerging therapeutic strategies. Medicine in Drug Discovery. 2025;26:100210. 10.1016/j.medidd.2025.100210.

[204] Amiri A, Bagherifar R, Ansari Dezfouli E, Kiaie SH, Jafari R, Ramezani R. Exosomes as bio-inspired nanocarriers for RNA delivery: preparation and applications. J Transl Med. 2022;20:125. 10.1186/s12967-022-03325-7. 35287692 10.1186/s12967-022-03325-7 PMC8919142

[205] Singh R, Pochampally R, Watabe K, Lu Z, Mo Y-Y. Exosome-mediated transfer of miR-10b promotes cell invasion in breast cancer. Mol Cancer. 2014;13:256. 10.1186/1476-4598-13-256. 25428807 10.1186/1476-4598-13-256 PMC4258287

[206] Wong GL, Abu Jalboush S, Lo H-W. Exosomal MicroRNAs and organotropism in breast cancer metastasis. Cancers (Basel). 2020;12:1827. 10.3390/cancers12071827. 32646059 10.3390/cancers12071827 PMC7408921

[207] Kulus M, Farzaneh M, Sheykhi-Sabzehpoush M, Ghaedrahmati F, Mehravar F, Józkowiak M, *et al*. Exosomes and non-coding RNAs: exploring their roles in human myocardial dysfunction. Biomed Pharmacother. 2025;183:117853. 10.1016/j.biopha.2025.117853. 39827809 10.1016/j.biopha.2025.117853

[208] Iqbal Z, Rehman K, Mahmood A, Shabbir M, Liang Y, Duan L, *et al*. Exosome for mRNA delivery: strategies and therapeutic applications. J Nanobiotechnology. 2024;22:395. 10.1186/s12951-024-02634-x. 38965553 10.1186/s12951-024-02634-x PMC11225225

[209] Paul A, Muralidharan A, Biswas A, Kamath BV, Joseph A, Alex AT. siRNA therapeutics and its challenges: recent advances in effective delivery for cancer therapy. Open Nano. 2022;7:100063. 10.1016/j.onano.2022.100063.

[210] Chae YK, Arya A, Malecek M-K, Shin DS, Carneiro B, Chandra S, *et al*. Repurposing metformin for cancer treatment: current clinical studies. Oncotarget. 2016;7:40767–80. 10.18632/oncotarget.8194. 27004404 10.18632/oncotarget.8194 PMC5130043

[211] Abbasi R, Nejati V, Rezaie J. Exosomes biogenesis was increased in metformin-treated human ovary cancer cells; possibly to mediate resistance. Cancer Cell Int. 2024;24:137. 10.1186/s12935-024-03312-6. 38627767 10.1186/s12935-024-03312-6 PMC11022479

[212] Juarez D, Fruman DA. Targeting the Mevalonate Pathway in Cancer. Trends Cancer. 2021;7:525–40. 10.1016/j.trecan.2020.11.008. 33358111 10.1016/j.trecan.2020.11.008 PMC8137523

[213] Valipour E, Ranjbar FE, Mousavi M, Ai J, Malekshahi ZV, Mokhberian N, *et al*. The anti-angiogenic effect of atorvastatin loaded exosomes on glioblastoma tumor cells: An in vitro 3D culture model. Microvasc Res. 2022;143:104385. 10.1016/j.mvr.2022.104385. 35609635 10.1016/j.mvr.2022.104385

[214] Santos MF, Rappa G, Karbanová J, Fontana S, Bella MAD, Pope MR, *et al*. Itraconazole inhibits nuclear delivery of extracellular vesicle cargo by disrupting the entry of late endosomes into the nucleoplasmic reticulum. J Extracell Vesicles. 2021;10:e12132. 10.1002/jev2.12132. 34429859 10.1002/jev2.12132 PMC8363911

[215] Jin M, Zeng B, Liu Y, Jin L, Hou Y, Liu C, *et al*. Co-delivery of repurposing Itraconazole and VEGF siRNA by composite nanoparticulate system for collaborative anti-angiogenesis and anti-tumor efficacy against breast cancer. Pharmaceutics. 2022;14:1369. 10.3390/pharmaceutics14071369. 10.3390/pharmaceutics14071369 PMC9317122 35890264

[216] Zhan Q, Yi K, Cui X, Li X, Yang S, Wang Q, *et al*. Blood exosomes-based targeted delivery of cPLA2 siRNA and metformin to modulate glioblastoma energy metabolism for tailoring personalized therapy. Neuro Oncol. 2022;24:1871–83. 10.1093/neuonc/noac071. 35312010 10.1093/neuonc/noac071 PMC9629419

[217] Yin L, He Z, Yi B, Xue L, Sun J. Simvastatin suppresses human breast cancer cell invasion by decreasing the expression of pituitary tumor-transforming gene 1. Front Pharmacol. 2020;11:574068. 10.3389/fphar.2020.574068. 33250768 10.3389/fphar.2020.574068 PMC7672329

[218] Aschenbrenner B, Negro G, Savic D, Sorokin M, Buzdin A, Ganswindt U, *et al*. Simvastatin Is Effective in Killing the Radioresistant Breast Carcinoma Cells. Radiol Oncol. 2021;55:305–16. 10.2478/raon-2021-0020. 33939900 10.2478/raon-2021-0020 PMC8366725

[219] Liu Y, Tang R, Cao Y, Wu N, Qin Q, Chen Y, *et al*. LIFU/MMP-2 dual-responsive release of repurposed drug disulfiram from nanodroplets for inhibiting vasculogenic mimicry and lung metastasis in triple-negative breast cancer. Journal of Nanobiotechnology. 2024;22:209. 10.1186/s12951-024-02492-7. 38664830 10.1186/s12951-024-02492-7 PMC11046851

[220] Bebawy G, Collier P, Williams PM, Burley JC, Needham D. Ldlr-targeted orlistat therapeutic nanoparticles: peptide selection, assembly, characterization, and cell-uptake in breast cancer cell lines. Int J Pharm. 2025;676:125574. 10.1016/j.ijpharm.2025.125574. 40239877 10.1016/j.ijpharm.2025.125574

[221] Bhargava-Shah A, Foygel K, Devulapally R, Paulmurugan R. Orlistat and antisense-miRNA-loaded PLGA-PEG nanoparticles for enhanced triple negative breast cancer therapy. Nanomedicine (Lond). 2016;11:235–47. 10.2217/nnm.15.193. 26787319 10.2217/nnm.15.193 PMC4910964

[222] Oskouie MN, Aghili Moghaddam NS, Butler AE, Zamani P, Sahebkar A. Therapeutic use of curcumin-encapsulated and curcumin-primed exosomes. J Cell Physiol. 2019;234:8182–91. 10.1002/jcp.27615. 30317632 10.1002/jcp.27615

[223] Ávila-Gálvez MÁ, Garay-Mayol B, Giménez-Bastida JA, Hazas M del CL de las, Mazarío-Gárgoles C, Brito MA, *et al*. Enhanced brain delivery and antiproliferative activity of resveratrol using milk-derived exosomes. Journal of Agriculture and Food Research. 2024;18:101370. 10.1016/j.jafr.2024.101370.

[224] Gonzalez Suarez N, Fernandez-Marrero Y, Hébert MPA, Roy M-E, Boudreau LH, Annabi B. EGCG inhibits the inflammation and senescence inducing properties of MDA-MB-231 triple-negative breast cancer (TNBC) cells-derived extracellular vesicles in human adipose-derived mesenchymal stem cells. Cancer Cell Int. 2023;23:240. 10.1186/s12935-023-03087-2. 37833751 10.1186/s12935-023-03087-2 PMC10576371

[225] Gao Z-S, Zhang C-J, Xia N, Tian H, Li D-Y, Lin J-Q, *et al*. Berberine-loaded M2 macrophage-derived exosomes for spinal cord injury therapy. Acta Biomater. 2021;126:211–23. 10.1016/j.actbio.2021.03.018. 33722788 10.1016/j.actbio.2021.03.018

[226] Zheng X, Zhao Y, Jia Y, Shao D, Zhang F, Sun M, *et al*. Biomimetic co-assembled nanodrug of doxorubicin and berberine suppresses chemotherapy-exacerbated breast cancer metastasis. Biomaterials. 2021;271:120716. 10.1016/j.biomaterials.2021.120716. 33621894 10.1016/j.biomaterials.2021.120716

[227] Kang X, Jadhav S, Annaji M, Huang C-H, Amin R, Shen J, *et al*. Advancing Cancer Therapy with Copper/Disulfiram Nanomedicines and Drug Delivery Systems. Pharmaceutics. 2023;15:1567. 10.3390/pharmaceutics15061567. 37376016 10.3390/pharmaceutics15061567 PMC10302862

[228] Zeng H, Guo S, Ren X, Wu Z, Liu S, Yao X. Current strategies for exosome cargo loading and targeting delivery. Cells. 2023;12:1416. 10.3390/cells12101416. 37408250 10.3390/cells12101416 PMC10216928

[229] Imran M, Rauf A, Khan IA, Shahbaz M, Qaisrani TB, Fatmawati S, *et al*. Thymoquinone: A novel strategy to combat cancer: A review. Biomedicine & Pharmacotherapy Elsevier BV. 2018;106:390–402. 10.1016/j.biopha.2018.06.159. 10.1016/j.biopha.2018.06.159 29966985

[230] Asaduzzaman Khan Md, Tania M, Fu S, Fu J. Thymoquinone, as an anticancer molecule: from basic research to clinical investigation. Oncotarget. Impact Journals, LLC; 2017;8:51907–19. 10.18632/oncotarget.17206. 10.18632/oncotarget.17206 PMC5584300 28881699

[231] Scott JL, Gupta RC, Aqil F, Jeyabalan J, Schultz DJ. Exosomal Delivery Enhances the Antiproliferative Effects of Acid-Hydrolyzed Apiaceae Spice Extracts in Breast Cancer Cells. Foods. 2024;13:2811. 10.3390/foods13172811. 39272578 10.3390/foods13172811 PMC11395330

[232] Nobili S, Lippi D, Witort E, Donnini M, Bausi L, Mini E, *et al*. Natural compounds for cancer treatment and prevention. Pharmacol Res. 2009;59:365–78. 10.1016/j.phrs.2009.01.017. 10.1016/j.phrs.2009.01.017 19429468

[233] Liu WK, Xu SX, Che CT, Elsevier BV. Anti-proliferative effect of ginseng saponins on human prostate cancer cell line. Life Sci. 2000;67:1297–306. 10.1016/s0024-3205(00)00720-7. 10972198 10.1016/s0024-3205(00)00720-7

[234] Thangapazham RL, Singh AK, Sharma A, Warren J, Gaddipati JP, Maheshwari RK, *et al*. Green tea polyphenols and its constituent epigallocatechin gallate inhibits proliferation of human breast cancer cells in vitro and in vivo. Cancer Lett. 2007;245:232–41. 10.1016/j.canlet.2006.01.027. 16519995 10.1016/j.canlet.2006.01.027

[235] Kim HI, Park J, Zhu Y, Wang X, Han Y, Zhang D, *et al*. Recent advances in extracellular vesicles for therapeutic cargo delivery. Exp Mol Med. 2024;56:836–49. 10.1038/s12276-024-01201-6. 38556545 10.1038/s12276-024-01201-6 PMC11059217

[236] Smyth T, Kullberg M, Malik N, Smith-Jones P, Graner MW, Anchordoquy TJ, *et al*. Biodistribution and delivery efficiency of unmodified tumor-derived exosomes. J Control Release. 2015;199:145–55. 10.1016/j.jconrel.2014.12.013. 25523519 10.1016/j.jconrel.2014.12.013 PMC4441346

[237] Le Saux S, Aarrass H, Lai-Kee-Him J, Bron P, Armengaud J, Miotello G, *et al*. Post-production modifications of murine mesenchymal stem cell (mMSC) derived extracellular vesicles (EVs) and impact on their cellular interaction. Biomaterials. 2020;231:119675. 10.1016/j.biomaterials.2019.119675. 31838346 10.1016/j.biomaterials.2019.119675

[238] Wang P, Wang H, Huang Q, Peng C, Yao L, Chen H, *et al*. Exosomes from M1-Polarized Macrophages Enhance Paclitaxel Antitumor Activity by Activating Macrophages-Mediated Inflammation. Theranostics Ivyspring International Publisher. 2019;9:1714–27. 10.7150/thno.30716. 10.7150/thno.30716 PMC6485189 31037133

[239] Zhao Y, Zheng Y, Zhu Y, Li H, Zhu H, Liu T. Docetaxel-loaded M1 macrophage-derived exosomes for a safe and efficient chemoimmunotherapy of breast cancer. J Nanobiotechnology. 2022;20:359. 10.1186/s12951-022-01526-2. 35918698 10.1186/s12951-022-01526-2 PMC9344780

[240] Aqil F, Munagala R, Jeyabalan J, Agrawal AK, Gupta R. Exosomes for the enhanced tissue bioavailability and efficacy of curcumin. AAPS J. 2017;19:1691–702. 10.1208/s12248-017-0154-9. 29047044 10.1208/s12248-017-0154-9

[241] Salarpour S, Forootanfar H, Pournamdari M, Ahmadi-Zeidabadi M, Esmaeeli M, Pardakhty A. Paclitaxel incorporated exosomes derived from glioblastoma cells: comparative study of two loading techniques. Daru. 2019;27:533–9. 10.1007/s40199-019-00280-5. 31317441 10.1007/s40199-019-00280-5 PMC6895332

[242] Abdul-Rahman T, Roy P, Herrera-Calderón RE, Khidri FF, Omotesho QA, Rumide TS, *et al*. Extracellular vesicle-mediated drug delivery in breast cancer theranostics. Discov Oncol. 2024;15:181. 10.1007/s12672-024-01007-y. 38780753 10.1007/s12672-024-01007-y PMC11116322

[243] Haney MJ, Klyachko NL, Zhao Y, Gupta R, Plotnikova EG, He Z, *et al*. Exosomes as Drug Delivery Vehicles for Parkinson’s Disease Therapy. J Control Release. 2015;207:18–30. 10.1016/j.jconrel.2015.03.033. 25836593 10.1016/j.jconrel.2015.03.033 PMC4430381

[244] Palakurthi SS, Shah B, Kapre S, Charbe N, Immanuel S, Pasham S, *et al*. A comprehensive review of challenges and advances in exosome-based drug delivery systems. Nanoscale Adv. 2024;6:5803–26. 10.1039/D4NA00501E. 10.1039/d4na00501e PMC11523810 39484149

[245] Lamichhane TN, Jeyaram A, Patel DB, Parajuli B, Livingston NK, Arumugasaamy N, *et al*. Oncogene Knockdown via Active Loading of Small RNAs into Extracellular Vesicles by Sonication. Cell Mol Bioeng. 2016;9:315–24. 10.1007/s12195-016-0457-4. 27800035 10.1007/s12195-016-0457-4 PMC5084850

[246] Haney MJ, Zhao Y, Jin YS, Li SM, Bago JR, Klyachko NL, *et al*. Macrophage-Derived Extracellular Vesicles as Drug Delivery Systems for Triple Negative Breast Cancer (TNBC) Therapy. J Neuroimmune Pharmacol. 2020;15:487–500. 10.1007/s11481-019-09884-9. 31722094 10.1007/s11481-019-09884-9

[247] Kim MS, Haney MJ, Zhao Y, Yuan D, Deygen I, Klyachko NL, *et al*. Engineering macrophage-derived exosomes for targeted paclitaxel delivery to pulmonary metastases: in vitro and in vivo evaluations. Nanomedicine. 2018;14:195–204. 10.1016/j.nano.2017.09.011. 28982587 10.1016/j.nano.2017.09.011

[248] Yu M, Gai C, Li Z, Ding D, Zheng J, Zhang W, *et al*. Targeted exosome-encapsulated erastin induced ferroptosis in triple negative breast cancer cells. Cancer Sci. 2019;110:3173–82. 10.1111/cas.14181. 31464035 10.1111/cas.14181 PMC6778638

[249] Uslu D, Abas B, Demi̇rbolat G, Cevik O. Effect of platelet exosomes loaded with doxorubicin as a targeted therapy on triple-negative breast cancer cells. Mol Diversity [Internet]. 2024 [cited 2025 Oct 17];28. 10.1007/s11030-022-10591-6. 10.1007/s11030-022-10591-6 36576666

[250] Aqil F, Munagala R, Jeyabalan J, Agrawal AK, Kyakulaga A-H, Wilcher SA, *et al*. Milk exosomes - Natural nanoparticles for siRNA delivery. Cancer Lett. 2019;449:186–95. 10.1016/j.canlet.2019.02.011. 30771430 10.1016/j.canlet.2019.02.011

[251] Pomatto MAC, Bussolati B, D’Antico S, Ghiotto S, Tetta C, Brizzi MF, *et al*. Improved Loading of Plasma-Derived Extracellular Vesicles to Encapsulate Antitumor miRNAs. Mol Ther Methods Clin Dev. 2019;13:133–44. 10.1016/j.omtm.2019.01.001. 30788382 10.1016/j.omtm.2019.01.001 PMC6370572

[252] Goh WJ, Lee CK, Zou S, Woon EC, Czarny B, Pastorin G. Doxorubicin-loaded cell-derived nanovesicles: an alternative targeted approach for anti-tumor therapy. Int J Nanomedicine. 2017;12:2759–67. 10.2147/IJN.S131786. 28435256 10.2147/IJN.S131786 PMC5388236

[253] Wang T, Larcher LM, Ma L, Veedu RN. Systematic screening of commonly used commercial transfection reagents towards efficient transfection of single-stranded oligonucleotides. Molecules. 2018;23:2564. 10.3390/molecules23102564. 30297632 10.3390/molecules23102564 PMC6222501

[254] Yang T, Fogarty B, LaForge B, Aziz S, Pham T, Lai L, *et al*. Delivery of Small Interfering RNA to Inhibit Vascular Endothelial Growth Factor in Zebrafish Using Natural Brain Endothelia Cell-Secreted Exosome Nanovesicles for the Treatment of Brain Cancer. AAPS J. 2017;19:475–86. 10.1208/s12248-016-0015-y. 27882487 10.1208/s12248-016-0015-y

[255] Podolak I, Galanty A, Sobolewska D. Saponins as cytotoxic agents: a review. Phytochem Rev. 2010;9:425–74. 10.1007/s11101-010-9183-z. 20835386 10.1007/s11101-010-9183-z PMC2928447

[256] Gharavi AT, Irian S, Niknejad A, Parang K, Salimi M. Harnessing exosomes as a platform for drug delivery in breast cancer: A systematic review for in vivo and in vitro studies. Molecular Therapy Oncology [Internet]. Elsevier; 2024 [cited 2025 Oct 17];32. 10.1016/j.omton.2024.200800. 10.1016/j.omton.2024.200800 PMC11067457 38706989

[257] Xu G, Jin J, Fu Z, Wang G, Lei X, Xu J, *et al*. Extracellular vesicle-based drug overview: research landscape, quality control and nonclinical evaluation strategies. Signal Transduct Target Ther. 2025;10:255. 10.1038/s41392-025-02312-w. 40804047 10.1038/s41392-025-02312-w PMC12350758

[258] Jang YJ, Cha BS, Kim D, Lee ES, Kim S, Han J, *et al*. Extracellular Vesicles, as Drug-Delivery Vehicles, Improve the Biological Activities of Astaxanthin. Antioxidants (Basel). 2023;12:473. 10.3390/antiox12020473. 36830031 10.3390/antiox12020473 PMC9952194

[259] Ashique S, Anand K. Radiolabelled extracellular vesicles as imaging modalities for precise targeted drug delivery. Pharmaceutics. 2023;15:1426. 10.3390/pharmaceutics15051426. 37242668 10.3390/pharmaceutics15051426 PMC10220982

[260] Kanchanapally R, Deshmukh SK, Chavva SR, Tyagi N, Srivastava SK, Patel GK, *et al*. Drug-loaded exosomal preparations from different cell types exhibit distinctive loading capability, yield, and antitumor efficacies: a comparative analysis. Int J Nanomedicine. 2019;14:531–41. 10.2147/ijn.s191313. 30666112 10.2147/IJN.S191313 PMC6333392

[261] Munagala R, Aqil F, Jeyabalan J, Gupta RC, Elsevier BV. Bovine milk-derived exosomes for drug delivery. Cancer Lett. 2016;371:48–61. 10.1016/j.canlet.2015.10.020. 26604130 10.1016/j.canlet.2015.10.020 PMC4706492

[262] Saari H, Lázaro-Ibáñez E, Viitala T, Vuorimaa-Laukkanen E, Siljander P, Yliperttula M, *et al*. Microvesicle- and exosome-mediated drug delivery enhances the cytotoxicity of paclitaxel in autologous prostate cancer cells. J Control Release. 2015;220:727–37. 10.1016/j.jconrel.2015.09.031. 26390807 10.1016/j.jconrel.2015.09.031

[263] Schindler C, Collinson A, Matthews C, Pointon A, Jenkinson L, Minter RR, *et al*. Exosomal delivery of doxorubicin enables rapid cell entry and enhanced in vitro potency. PLOS ONE. Public Libr Sci. (PLoS); 2019;14:e0214545. 10.1371/journal.pone.0214545. 10.1371/journal.pone.0214545 PMC6440694 30925190

[264] Sancho-Albero M, Encabo-Berzosa M, Beltrán-Visiedo M, Fernández-Messina L, Sebastián V, Sánchez-Madrid F, *et al*. Efficient encapsulation of theranostic nanoparticles in cell-derived exosomes: leveraging the exosomal biogenesis pathway to obtain hollow gold nanoparticle-hybrids. Nanoscale. 2019;11:18825–36. 10.1039/c9nr06183e. 10.1039/c9nr06183e 31595912

[265] Fuhrmann G, Serio A, Mazo M, Nair R, Stevens MM, Elsevier BV. Active loading into extracellular vesicles significantly improves the cellular uptake and photodynamic effect of porphyrins. J Control Release. 2015;205:35–44. 10.1016/j.jconrel.2014.11.029. 25483424 10.1016/j.jconrel.2014.11.029

[266] Chen C, Sun M, Wang J, Su L, Lin J, Yan X. Active cargo loading into extracellular vesicles: highlights the heterogeneous encapsulation behaviour. J Extracell Vesicles. 2021;10:e12163. 10.1002/jev2.12163. 34719860 10.1002/jev2.12163 PMC8558234

[267] Lennaárd AJ, Mamand DR, Wiklander RJ, Andaloussi SE, Wiklander OPB, Multidisciplinary Digital Publishing Institute. Optimised electroporation for loading of extracellular vesicles with doxorubicin. Pharmaceutics. 2021. 10.3390/pharmaceutics14010038. 35056933 10.3390/pharmaceutics14010038 PMC8780628

[268] Reshke R, Taylor JA, Savard A, Guo H, Rhym LH, Kowalski PS, *et al*. Reduction of the therapeutic dose of silencing RNA by packaging it in extracellular vesicles via a pre-microRNA backbone. Nat Biomed Eng. 2020;4:52–68. 10.1038/s41551-019-0502-4. 31937944 10.1038/s41551-019-0502-4

[269] Kim G, Kim M, Lee Y, Byun JW, Hwang DW, Lee M. Systemic delivery of microRNA-21 antisense oligonucleotides to the brain using T7-peptide decorated exosomes. J Control Release. 2020;317:273–81. 10.1016/j.jconrel.2019.11.009. 31730913 10.1016/j.jconrel.2019.11.009

[270] Yang Z, Shi J, Xie J, Wang Y, Sun J, Liu T, *et al*. Large-scale generation of functional mRNA-encapsulating exosomes via cellular nanoporation. Nat Biomed Eng. 2020;4:69–83. 10.1038/s41551-019-0485-1. 31844155 10.1038/s41551-019-0485-1 PMC7080209

[271] Gul R, Bashir H, Sarfraz M, Shaikh AJ, Bin Jardan YA, Hussain Z, *et al*. Human plasma derived exosomes: Impact of active and passive drug loading approaches on drug delivery. Saudi Pharmaceutical Journal. 2024;32:102096. 10.1016/j.jsps.2024.102096. 38757071 10.1016/j.jsps.2024.102096 PMC11097067

[272] Thakur A, Sidu RK, Zou H, Alam MK, Yang M, Lee Y. Inhibition of Glioma Cells’ Proliferation by Doxorubicin-Loaded Exosomes via Microfluidics. Int J Nanomedicine. 2020;15:8331–43. 10.2147/IJN.S263956. 33149579 10.2147/IJN.S263956 PMC7605152

[273] Liu A, Yang G, Liu Y, Liu T. Research progress in membrane fusion-based hybrid exosomes for drug delivery systems. Front Bioeng Biotechnol. 2022;10:939441. 10.3389/fbioe.2022.939441. 36051588 10.3389/fbioe.2022.939441 PMC9424752

[274] Guarro M, van Veen S, Borrós S, Albertazzi L, Lecina M, Fornaguera C. Quantitative and qualitative comparison of mRNA loading techniques into extracellular vesicles. Biomed Pharmacother. 2025;193:118813. 10.1016/j.biopha.2025.118813. 41297422 10.1016/j.biopha.2025.118813

[275] Bernal-Chávez SA, Romero-Montero A, Hernández-Parra H, Peña-Corona SI, Del Prado-Audelo ML, Alcalá-Alcalá S, *et al*. Enhancing chemical and physical stability of pharmaceuticals using freeze-thaw method: challenges and opportunities for process optimization through quality by design approach. J Biol Eng. 2023;17:35. 10.1186/s13036-023-00353-9. 37221599 10.1186/s13036-023-00353-9 PMC10207779

[276] Chen Z, Xiong M, Tian J, Song D, Duan S, Zhang L. Encapsulation and assessment of therapeutic cargo in engineered exosomes: a systematic review. J Nanobiotechnol. 2024;22:18. 10.1186/s12951-023-02259-6. 10.1186/s12951-023-02259-6 PMC10765779 38172932

[277] Sato YT, Umezaki K, Sawada S, Mukai S, Sasaki Y, Harada N, *et al.* Engineering hybrid exosomes by membrane fusion with liposomes. Sci Rep. 2016;6:21933. 10.1038/srep21933. 26911358 10.1038/srep21933 PMC4766490

[278] Zuppone S, Zarovni N, Noguchi K, Loria F, Morasso C, Lõhmus A, *et al*. Novel loading protocol combines highly efficient encapsulation of exogenous therapeutic toxin with preservation of extracellular vesicles properties, uptake and cargo activity. Discov Nano. 2024;19:76. 10.1186/s11671-024-04022-8. 38691254 10.1186/s11671-024-04022-8 PMC11063024

[279] Piunti C, Micheli S, Giancaterino S, Fusco P, Boi C, Cimetta E, *et al.* Microfluidic loading of verteporfin into extracellular vesicles for neuroblastoma therapy. Lab Chip. 2025;25:1718–27. 10.1039/D4LC01103A. 40007431 10.1039/d4lc01103a PMC11862876

[280] Mukerjee N, Maitra S, Kaur M, Rekha MM, Soothwal P, Arora I, *et al*. Click chemistry-based modified exosomes: Towards enhancing precision in cancer theranostics. Chem Eng J. 2025;512:160915. 10.1016/j.cej.2025.160915.

[281] Lewis ND, Sia CL, Kirwin K, Haupt S, Mahimkar G, Zi T, *et al*. Exosome Surface Display of IL12 Results in Tumor-Retained Pharmacology with Superior Potency and Limited Systemic Exposure Compared with Recombinant IL12. Mol Cancer Ther. 2021;20:523–34. 10.1158/1535-7163.MCT-20-0484. 33443094 10.1158/1535-7163.MCT-20-0484

[282] Dooley K, McConnell RE, Xu K, Lewis ND, Haupt S, Youniss MR, *et al*. A versatile platform for generating engineered extracellular vesicles with defined therapeutic properties. Mol Ther. 2021;29:1729–43. 10.1016/j.ymthe.2021.01.020. 33484965 10.1016/j.ymthe.2021.01.020 PMC8116569

[283] Tran NH, Nguyen DD, Nguyen NM, Tran C, Nguyen Thi NT, Ho DT, *et al*. Dual-targeting exosomes for improved drug delivery in breast cancer. Nanomedicine (Lond). 2023;18:599–611. 10.2217/nnm-2022-0328. 37194929 10.2217/nnm-2022-0328

[284] Saha M, Ghosh SS. Targeting tumor microenvironment of metastatic triple negative breast cancer cells via exosomes derived from non-invasive breast cancer cells for multi-drug resistance inhibition and enhancing drug susceptibility. J Drug Deliv Sci Technol. 2023;89:105028. 10.1016/j.jddst.2023.105028.

[285] Hu J, Yang S, Zhu J, Chai J, Luan J, Wang Y. An engineered-exosome disguised multifunctional nanoplatform for enhanced chemo-photothermal therapy of triple-negative breast cancer. Mater Des. 2025;251:113633. 10.1016/j.matdes.2025.113633.

[286] Verma N, Tamang R, Mehata AK, Setia A, Koch B, Muthu MS. Exosomes fused liposomes: formulation, stability studies and theranostic evaluation for breast cancer applications. Int J Pharm. 2025;682:125907. 10.1016/j.ijpharm.2025.125907. 40582518 10.1016/j.ijpharm.2025.125907

[287] Xu M, Bai L, Sun M, Yan X, Xiong Y, Wang Y, *et al*. ROS-responsive biomimetic nanocomplexes of liposomes and macrophage-derived exosomes for combination breast cancer therapy. Int J Nanomedicine. 2025;Volume 20:5161–80. 10.2147/ijn.s514375. 10.2147/IJN.S514375 PMC12036690 40297405

[288] Nguyen Cao TG, Kang JH, Kim W, Lim J, Kang SJ, You JY, *et al*. Engineered extracellular vesicle-based sonotheranostics for dual stimuli-sensitive drug release and photoacoustic imaging-guided chemo-sonodynamic cancer therapy. Theranostics Ivyspring International Publisher. 2022;12:1247–66. 10.7150/thno.65516. 10.7150/thno.65516 PMC8771566 35154485

[289] St-Denis-Bissonnette F, Khoury R, Mediratta K, El-Sahli S, Wang L, Lavoie JR, *et al*. Applications of extracellular vesicles in triple-negative breast cancer. Cancers. 2022;14:451. 10.3390/cancers14020451. 35053616 10.3390/cancers14020451 PMC8773485

[290] Liu Q, Dai G, Wu Y, Zhang M, Yang M, Wang X, *et al*. iRGD-modified exosomes-delivered BCL6 siRNA inhibit the progression of diffuse large B-cell lymphoma. Front Oncol. 2022;12:822805. 10.3389/fonc.2022.822805. 35982974 10.3389/fonc.2022.822805 PMC9378967

[291] Dutta S, Warshall C, Bandyopadhyay C, Dutta D, Chandran B, Public Library of Science (PLoS). Interactions between exosomes from breast cancer cells and primary mammary epithelial cells leads to generation of reactive oxygen species which induce DNA damage response, stabilization of p53 and autophagy in epithelial cells. PLoS One. 2014;9:e97580. 10.1371/journal.pone.0097580. 24831807 10.1371/journal.pone.0097580 PMC4022578

[292] Shafiei M, Ansari MNM, Razak SIA, Khan MUA, MDPI AG. A comprehensive review on the applications of exosomes and liposomes in regenerative medicine and tissue engineering. Polymers. 2021;13:2529. 10.3390/polym13152529. 34372132 10.3390/polym13152529 PMC8347192

[293] Wang Y, Ji Q, Yan C, Ji P, Nature Publishing Group. Biomimetic intelligent nanoplatform with cascade amplification effect for tumor synergy therapy. Sci Rep. 2024;14:31067. 10.1038/s41598-024-82291-0. 39730928 10.1038/s41598-024-82291-0 PMC11680698

[294] Tenchov R, Sasso JM, Wang X, Liaw W-S, Chen C-A, Zhou QA, *et al*. Exosomes─nature’s lipid nanoparticles, a rising star in drug delivery and diagnostics. ACS Nano. 2022;16:17802–46. 10.1021/acsnano.2c08774. 36354238 10.1021/acsnano.2c08774 PMC9706680

[295] Abdel-Bar HM, Tandiono S, Liam-Or R, Cheung CCL, Hassuneh OWM, Lyu Q, *et al*. Optimizing exosome lipid hybrid nanoparticles for enhanced siRNA delivery and improved therapeutic anticancer efficacy *in vivo*. ACS Nano. 2025;19:42658–74. 10.1021/acsnano.5c16991. 10.1021/acsnano.5c16991 PMC12774390 41378821

[296] Son G, Song J, Park JC, Kim HN, Kim H. Fusogenic lipid nanoparticles for rapid delivery of large therapeutic molecules to exosomes. Nat Commun. 2025;16:4799. 10.1038/s41467-025-59489-5. 40410169 10.1038/s41467-025-59489-5 PMC12102247

[297] Wang X, Munson MJ, Friis K, Marzeda A, Silva AM, Kohl F, *et al.* Hybrid extracellular vesicles for efficient loading and functional delivery of mRNA. J Extracell Vesicles. 2025;14:e70201. 10.1002/jev2.70201. 41392576 10.1002/jev2.70201 PMC12703132

[298] Sun W, Huang S, Sun Z, Zhao Q, Li J, Wen B, *et al*. Exosome platform with three-in-one functionality of mRNA therapy, immune checkpoint blockade, and mild photothermal therapy for the treatment of triple-negative breast cancer. ACS Appl Mater Interfaces. 2025;17:10328–41. 10.1021/acsami.4c17804. 10.1021/acsami.4c17804 39905637

[299] Hench IB, Hench J, Tolnay M. Liquid biopsy in clinical management of breast, lung, and colorectal cancer. Front Med (Lausanne). 2018;5:9. 10.3389/fmed.2018.00009. 29441349 10.3389/fmed.2018.00009 PMC5797586

[300] Loric S, Denis JA, Desbene C, Sabbah M, Conti M. Extracellular vesicles in breast cancer: from biology and function to clinical diagnosis and therapeutic management. Int J Mol Sci. 2023;24:7208. 10.3390/ijms24087208. 37108371 10.3390/ijms24087208 PMC10139222

[301] Rashed H, Bayraktar E, Helal K, Abd-Ellah MF, Amero P, Chavez-Reyes A, *et al*. Exosomes: from garbage bins to promising therapeutic targets. Int J Mol Sci. 2017;18:538. 10.3390/ijms18030538. 28257101 10.3390/ijms18030538 PMC5372554

[302] Nowak M, Górczyńska J, Kołodzińska K, Rubin J, Choromańska A. Extracellular vesicles as drug transporters. Int J Mol Sci. 2023;24:10267. 10.3390/ijms241210267. 37373411 10.3390/ijms241210267 PMC10299356

[303] Boukouris S, Mathivanan S. Exosomes in bodily fluids are a highly stable resource of disease biomarkers. Proteomics Clin Appl. 2015;9:358–67. 10.1002/prca.201400114. 25684126 10.1002/prca.201400114 PMC5502131

[304] Lee J-E, Moon P-G, Cho Y-E, Kim Y-B, Kim I-S, Park H, *et al*. Identification of EDIL3 on extracellular vesicles involved in breast cancer cell invasion. J Proteomics. 2016;131:17–28. 10.1016/j.jprot.2015.10.005. 26463135 10.1016/j.jprot.2015.10.005

[305] Moon P-G, Lee J-E, Cho Y-E, Lee SJ, Chae YS, Jung JH, *et al*. Fibronectin on circulating extracellular vesicles as a liquid biopsy to detect breast cancer. Oncotarget. 2016;7:40189–99. 10.18632/oncotarget.9561. 27250024 10.18632/oncotarget.9561 PMC5130002

[306] Acuña RA, Varas-Godoy M, Berthoud VM, Alfaro IE, Retamal MA. Connexin-46 contained in extracellular vesicles enhance malignancy features in breast cancer cells. Biomolecules. 2020;10:676. 10.3390/biom10050676. 32353936 10.3390/biom10050676 PMC7277863

[307] Etayash H, McGee AR, Kaur K, Thundat T, The Royal Society of Chemistry. Nanomechanical sandwich assay for multiple cancer biomarkers in breast cancer cell-derived exosomes. Nanoscale. 2016;8:15137–41. 10.1039/C6NR03478K. 27492928 10.1039/c6nr03478k

[308] Kibria G, Ramos EK, Lee KE, Bedoyan S, Huang S, Samaeekia R, *et al*. A rapid, automated surface protein profiling of single circulating exosomes in human blood. Sci Rep. 2016;6:36502. 10.1038/srep36502. 27819324 10.1038/srep36502 PMC5098148

[309] Attar FA, Irani S, Oloomi M, Bolhassani A, Geranpayeh L, Atyabi F. Doxorubicin loaded exosomes inhibit cancer-associated fibroblasts growth: in vitro and in vivo study. Cancer Cell Int. 2025;25:72. 10.1186/s12935-025-03689-y. 40016747 10.1186/s12935-025-03689-y PMC11869484

[310] Kumar DN, Chaudhuri A, Dehari D, Shekher A, Gupta SC, Majumdar S, *et al*. Combination therapy comprising paclitaxel and 5-fluorouracil by using folic acid functionalized bovine milk exosomes improves the therapeutic efficacy against breast cancer. Life (Basel). 2022;12:1143. 10.3390/life12081143. 36013322 10.3390/life12081143 PMC9410314

[311] Wang X, Li D, Li G, Chen J, Yang Y, Bian L, *et al*. Enhanced therapeutic potential of hybrid exosomes loaded with paclitaxel for cancer therapy. Int J Mol Sci. 2024;25:3645. 10.3390/ijms25073645. 38612457 10.3390/ijms25073645 PMC11012016

[312] Ferreira D, Santos-Pereira C, Costa M, Afonso J, Yang S, Hensel J, *et al*. Exosomes modified with anti-MEK1 siRNA lead to an effective silencing of triple negative breast cancer cells. Biomaterials Advances. 2023;154:213643. 10.1016/j.bioadv.2023.213643. 37778291 10.1016/j.bioadv.2023.213643

[313] Mohan S, Khamjan NA, Abdelwahab SI, Taha MME, Moshi JM, Alshahrani AF, *et al*. Clinical frontiers of exosome research: a comprehensive analysis of human trials in diagnostics, therapeutics, and regenerative medicine. J Pharmacol Pharmacother. 2025(2). 10.1177/0976500X251361201.

[314] Rodríguez-Martínez A, de Miguel-Pérez D, Ortega FG, García-Puche JL, Robles-Fernández I, Exposito J, *et al*. Exosomal miRNA profile as complementary tool in the diagnostic and prediction of treatment response in localized breast cancer under neoadjuvant chemotherapy. Breast Cancer Res. 2019;21:21. 10.1186/s13058-019-1109-0. 30728048 10.1186/s13058-019-1109-0 PMC6366103

[315] Weitsman G, Barber PR, Nguyen LK, Lawler K, Patel G, Woodman N, *et al*. HER2-HER3 dimer quantification by FLIM-FRET predicts breast cancer metastatic relapse independently of HER2 IHC status. Oncotarget. 2016;7:51012–26. 10.18632/oncotarget.9963. 10.18632/oncotarget.9963 PMC5239455 27618787

[316] Tripathy A, Corkos P, Blouw B, Montgomery DA, Moore M, Hedrick MH, *et al*. Longitudinal CSF tumor cell enumeration and mutational analysis as a driver for leptomeningeal disease management. Cancers. 2025;17:825. 10.3390/cancers17050825. 10.3390/cancers17050825 PMC11899081 40075672

[317] Lin Y, Zhang Q, Chen H, Liu S, Peng K, Wang X, *et al*. Multi-cancer early detection based on serum surface-enhanced Raman spectroscopy with deep learning: a large-scale case–control study. BMC Med. 2025;23:97. 10.1186/s12916-025-03887-5. 39984977 10.1186/s12916-025-03887-5 PMC11846373

[318] Eubanks SE. A Pilot Study of Tumor-Derived Exosomes as Diagnostic and Prognostic Markers in Breast Cancer Patients Receiving Neoadjuvant Chemotherapy [Internet]. clinicaltrials.gov; 2017 July. Report No.: NCT01344109. https://clinicaltrials.gov/study/NCT01344109. Accessed 30 Oct 2025

[319] JI-YEON. Development of a Prognostic and Predictive Biomarker for Locally Advanced Breast Cancer Patients Treated With Neoadjuvant Chemotherapy Using Exosome [Internet]. clinicaltrials.gov; 2023 July. Report No.: NCT05955521. https://clinicaltrials.gov/study/NCT05955521. Accessed 30 Oct 2025

[320] Centre Oscar Lambret. Feasibility of Exosome Analysis in Cerebrospinal Fluid During the Diagnostic Workup of Metastatic Meningitis From Breast Cancer [Internet]. clinicaltrials.gov; 2025 Sept. Report No.: NCT05286684. https://clinicaltrials.gov/study/NCT05286684. Accessed 30 Oct 2025

[321] Centre Oscar Lambret. Omic Technologies to Track Resistance to Palbociclib in Metastatic Breast Cancer (OMERIC): A Cohort Study [Internet]. clinicaltrials.gov; 2024 Dec. Report No.: NCT04653740. https://clinicaltrials.gov/study/NCT04653740. Accessed 30 Oct 2025

[322] Poitiers University Hospital. Interest of Circulating Tumor DNA in Digestive and Gynecologic/Breast Cancer [Internet]. clinicaltrials.gov; 2024 Feb. Report No.: NCT04530890. https://clinicaltrials.gov/study/NCT04530890. Accessed 30 Oct 2025

[323] Centre Oscar Lambret. Benefit of Analyzing Exosomes in the Cerebrospinal Fluid During the Medical Care of Breast Cancer Patients With Suspicion of Leptomeningeal Metastasis. [Internet]. clinicaltrials.gov; 2025 Sept. Report No.: NCT03974204. https://clinicaltrials.gov/study/NCT03974204. Accessed 30 Oct 2025

[324] Connolly E. Effects of MK-3475 (Pembrolizumab) on the Breast Tumor Microenvironment in Triple Negative Breast Cancer With and Without Intra-operative RT: a Window of Opportunity Study [Internet]. clinicaltrials.gov; 2024 June. Report No.: NCT02977468. https://clinicaltrials.gov/study/NCT02977468. Accessed 30 Oct 2025

[325] Im Y-H. Genetic Characteristics of Metastatic Breast Cancer Patients [Internet]. clinicaltrials.gov; 2020 Feb. Report No.: NCT04258735. https://clinicaltrials.gov/study/NCT04258735. Accessed 30 Oct 2025

[326] Choi H, Choi Y, Yim HY, Mirzaaghasi A, Yoo J-K, Choi C. Biodistribution of exosomes and engineering strategies for targeted delivery of therapeutic exosomes. Tissue Eng Regen Med. 2021;18:499–511. 10.1007/s13770-021-00361-0. 34260047 10.1007/s13770-021-00361-0 PMC8325750

[327] Nicolini A, Ferrari P, Biava PM. Exosomes and cell communication: from tumour-derived exosomes and their role in tumour progression to the use of exosomal cargo for cancer treatment. Cancers (Basel). 2021;13:822. 10.3390/cancers13040822. 33669294 10.3390/cancers13040822 PMC7920050

[328] Lee KWA, Chan LKW, Hung LC, Phoebe LKW, Park Y, Yi K-H. Clinical Applications of Exosomes: A Critical Review. Int J Mol Sci. 2024;25:7794. 10.3390/ijms25147794. 39063033 10.3390/ijms25147794 PMC11277529

[329] Zhang Y, Lan M, Chen Y. Minimal information for studies of extracellular vesicles (MISEV): ten-year evolution (2014–2023). Pharmaceutics. 2024;16:1394. 10.3390/pharmaceutics16111394. 39598518 10.3390/pharmaceutics16111394 PMC11597804

